# Preclinical extracellular matrix-based treatment strategies for myocardial infarction: a systematic review and meta-analysis

**DOI:** 10.1038/s43856-025-00812-y

**Published:** 2025-03-30

**Authors:** Atze van der Pol, Marijn C. Peters, Ignasi Jorba, Anke M. Smits, Niels P. van der Kaaij, Marie-Jose Goumans, Kimberley E. Wever, Carlijn V. C. Bouten

**Affiliations:** 1 Soft Tissue Engineering and Mechanobiology, Department of Biomedical Engineering, Eindhoven University of Technology, Eindhoven, The Netherlands; 2 Institute for Complex Molecular Systems, Eindhoven University of Technology, Eindhoven, The Netherlands; 3 Department of Cardiothoracic Surgery, Regenerative Medicine Centre, University Medical Center Utrecht, Utrecht, The Netherlands; 4 Unitat de Biofísica i Bioenginyeria, Facultat de Medicina i Ciències de la Salut, Universitat de Barcelona, 08036 Barcelona, Spain; 5 Department of Cell and Chemical Biology, Leiden University Medical Center, Leiden, The Netherlands; 6 Department of Anesthesiology, Pain and Palliative Medicine, Radboud University Medical Center, Nijmegen, Gelderland The Netherlands

**Keywords:** Cardiac regeneration, Heart failure

## Abstract

**Background:**

Administrating extracellular matrix (ECM) to restore cardiac function post-myocardial infarction (MI) shows promise, however study variability obscures its true impact. We therefore conducted a systematic review and meta-analysis of preclinical studies to assess the effects of ECM treatments on cardiac function and tissue homeostasis post-MI.

**Methods:**

We searched PubMed and SCOPUS from inception to June 28, 2024, for animal studies describing ECM treatment post-MI (pre-registered on PROSPERO, CRD42022368400). Random effects meta-analyses compared ECM treatment to controls regarding left ventricular ejection fraction (LVEF), fractional shortening, infarct size, stroke volume, and left ventricular wall thickness. Subgroup analyses examined the influence of sex, species, ECM source, and administration method. Funnel plots and Egger’s regression assessed publication bias.

**Results:**

We identify 88 articles which meet our inclusion criteria. These studies describe the use of rats (51%), mice (38%), and pigs (11%). 44% of studies use males, 34% females, 5% both sexes, and 17% did not report sex. Most studies employ permanent MI models (85%) over ischemia reperfusion models (15%), and deliver ECM via intramyocardial injection (59%), cardiac patch (39%), cardiac sleeve (1%), or osmotic pump (1%). Our meta-analysis demonstrates that ECM treatment significantly improves LVEF (MD: 10.9%, 95% CI: [8.7%;13.0%]; *p* = 8.057e-24), fractional shortening (MD: 8.2%, 95% CI: [5.6%; 10.9%]; *p* = 1.751e-09), stroke volume (SMD 0.6, 95% CI: [0.2;1.0], *p* = 0.004), left ventricular wall thickening (SMD 1.2, 95% CI: [0.9; 1.5], *p* = 1.321e-17), while reducing infarct size (-11.7%, 95% CI: [-14.7%;-8.6%], *p* = 3.699e-14). We find no significant differences between the various subgroups and no indication of publication bias.

**Conclusions:**

ECM-based treatments significantly enhance cardiac function and tissue homeostasis in preclinical post-MI models, supporting further research toward clinical translation.

## Plain language summary

A heart attack leads to loss of cells that control heartbeat and the formation of unhealthy scar tissue that can cause heart failure. Traditional treatment strategies focusing on replacing cell loss show limited improvement in heart function. Recent approaches to restore the balance in heart tissue by supplying a healthy support structure, known as the extracellular matrix, show promising results. However, differences in study design make it hard to determine effectiveness across groups. By analysing studies in animal models thoroughly, we find that treatments involved in supplying healthy support structure significantly improve heart function in preclinical models by improving the heart’s ability to pump blood and reduce scar tissue formation, regardless of species, sex, or the source of the extracellular matrix. These findings provide a strong foundation for future research focused on improving the support matrix of the heart to restore heart function.

## Abstract (web summary)

van der Pol et al. present a systematic review and meta-analysis of the literature on the pre-clinical efficacy of extracellular matrix-based treatment strategies for myocardial infarction. Authors show improvements following extracellular matrix-based treatment strategies irrespective of species, sex or extracellular matrix source.

## Introduction

Myocardial infarction (MI) remains one of the leading causes of cardiovascular death worldwide^1,2^. An MI results in the acute loss of cardiomyocytes and the formation of a fibrous scar^3,4^. This process of remodelling is a direct result of the migration of cardiac fibroblasts within the site of injury^5^. These cardiac fibroblasts start degrading the damaged microenvironment (i.e., the extracellular matrix), through the active expression of matrix metalloproteinase, while concurrently expressing and depositing a highly fibrotic extracellular matrix (ECM), comprised primarily of collagen^6,7^. This highly fibrotic scar tissue, combined with the lack of functioning cardiomyocytes, eventually results in a diminished cardiac muscle function and heart failure (HF).

Over the past decades, and with the discovery of stem cells and their pluripotent potential, much effort has been placed on cardiac regenerative therapies to treat MI through stem cell-based approaches [i.e. embryonic stem cells (ESCs), induced pluripotent stem cells (iPSCs), bone marrow-derived stem cells, and most recently cardiac stem cells (CSCs)]^8,9^. The early significant improvements observed in the pre-clinical setting quickly transitioned into early clinical trials^10^. However, to date, clinical studies utilizing cell-based strategies have resulted in limited cardiac functional improvement^11–16^. Besides tackling MI from a cellular perspective, recent advances in developmental biology and tissue regeneration have characterized the ECM as possessing an equally, if not greater, role in the restoration of damaged tissues^17^.

Although the ECM has historically been considered a biomechanical entity, whose main function is to provide structural support, the ability of the ECM to control cellular behaviour is now being increasingly recognized^3,18,19^. As a matter of fact, ECM homeostasis is crucial for keeping balance of many essential cellular processes, including, but not limited to, spatiotemporal development of tissues/organs in both prenatal and postnatal life, and restoring homeostasis following tissue damages and injuries^20,21^. Under pathological conditions, as with MI, cardiac fibroblasts produce excessive ECM with aberrant organization, which is considered the main obstacle to normal tissue regeneration^22^. Therefore, restoring the ECM homeostasis could hold the key to restoring the heart following an MI.

Unlike cell-based approaches, which rapidly transitioned from pre-clinical to clinical trials, ECM-based strategies to restore ECM homeostasis and thereby repair MI-induced injury, have remained mainly in the realm of basic and pre-clinical research^23,24^. However, these noncellular ECM-based strategies, if proven to be effective, would possess great advantages over their cellular counterparts, including reduced immunogenicity and donor variability^17^. To date, a variety of pre-clinical studies have been conducted utilizing a broad range of ECM-based treatment strategies; including variations in ECM source [single ECM components (i.e. collagen, fibrin, hyaluronic acid, agrin, or fibronectin) or decellularized whole organ ECM (dECM)], animal model (mouse, rat or pig), type of myocardial injury (MI or ischemia/reperfusion injury), method for administration (cardiac patches, intramyocardial injections or osmotic pumps), and timing of the treatment (directly, hours or weeks after MI). As such, it is very difficult to ascertain whether ECM-based strategies truly induce improvement of cardiac function and tissue homeostasis post-MI. To that end, and to help future transition into the clinical setting, we performed a systematic review and meta-analysis on the pre-clinical efficacy of ECM-based treatment strategies to treat MI.

We identify 88 out of the 2866 unique articles that meet our inclusion criteria covering a variety of animal models (mice, rats, and pigs), sexes, myocardial injury models (myocardial infarction or ischemia reperfusion), and ECM delivery methods (intramyocardial injection, cardiac patch, cardiac sleeve, or osmotic pump). Our meta-analysis demonstrates that ECM treatment significantly improves left ventricular ejection fraction (MD: 10.9%, 95% CI: [8.7%;13.0%]; *p* = 8.057e-24), fractional shortening (MD: 8.2%, 95% CI: [5.6%; 10.9%]; *p* = 1.751e-09), stroke volume (SMD 0.6, 95% CI: [0.2;1.0], *p* = 0.004), left ventricular wall thickening (SMD 1.2, 95% CI: [0.9; 1.5], *p* = 1.321e-17), while reducing infarct size (−11.7%, 95% CI: [-14.7%;-8.6%], *p* = 3.699e-14). These effects are irrespective of species, sex or ECM source. Combined, these results establish a foundation for future research directions targeting tissue homeostasis as a means to repair the injured myocardium.

## Methods

The review protocol for this meta-analysis was preregistered on PROSPERO on the 20th of December 2022 (CRD42022368400) and reported according to the PRISMA 2020 guidelines^25^. The protocol was written before performing the search, but submitted to PROSPERO afterwards to allow us to confirm that the number of records retrieved was feasible to screen whilst achieving sufficient sensitivity.

### Adjustments to the review protocol

An amendment was made to the original study protocol on the 16th of May 2023, regarding the timing of treatment categorizations. Initially, we had stated that we would categorize the timing of treatment as early (within 1 hour post myocardial infarction), middle (within 2 h post myocardial infarction), and late (treatment taking place any time after 2 h post myocardial infarction). However, following discussion with our clinical collaborators, we opted to change to more clinically relevant intervention time points, namely: early (within first day), middle (within first week), and late (treatment taking place after one week following myocardial infarction).

### Search strategy and article selection

An initial PubMed and Scopus search was performed on the 6^th^ of July 2022, and updated on the 28^th^ of June 2024. Both PubMed and Scopus databases were searched to identify all original articles related to ECM related strategies in animals for cardiac regeneration following myocardial infarction. We used keywords for heart injury, repair, ECM and animal models, without time or language restriction (Syntax see Supplementary file 1–4). The results of the search were uploaded into the Rayyan citation software^26^. For both the title and abstract screening, as well as the full-text screening, references were allocated to three independent reviewers (IJ, MP, and AP), ensuring that each reference was screened by at least two independent reviewers. At both stages of the screening process, eligibility for inclusion was assessed according to the inclusion criteria (PROSPERO - CRD42022368400). Discrepancies were resolved by consensus decision.

### Data extraction

Data was extracted by one reviewer (AP). After data extraction, two independent reviewers (IJ and MP) verified the data entries.

A set of 30 study characteristics were extracted from all eligible studies, comprising study information, animal characteristics, myocardial injury model, ECM treatment characteristics, follow-up time and functional outcome methodology (Supplementary Data 1). Next, we moved on to extract data on the primary outcome: left ventricular ejection fraction (LVEF) and animal survival. Finally, we extracted data related to our secondary outcomes: infarct size, fractional shortening, stroke volume, wall thickness, heart weight, and heart rate. If necessary, data were estimated from graphics. Accordingly, standard deviations were determined or recalculated from standard errors.

### Analysis of the quality of reporting and internal validity

The risk of bias was assessed for all studies, which were included in the systematic review after the full-text screening. The internal validity of the included studies was assessed in duplicate by three independent reviewers (IJ, MP, and AP), ensuring each study was assessed by at least two independent reviewers, using SYRCLE’s risk of bias tool^27^. Disagreements between reviewers were resolved through discussion.

To prevent statistical bias and underestimation or overestimation of the experimental data, it is essential to properly blind and randomize the experimental procedure^28^. However, in most cases both aspects fail to be properly reported in pre-clinical animal studies^29^. We used SYRCLE’s risk of bias tool to determine the risk of bias in all included studies^27^. Initially, we assessed the reporting of quality indicators, namely the reporting of any randomization, any blinding, sample size calculation, conflict of interest statement, and pre-registration of study protocol. Each component was scored with a “Yes” or “No,” indicating whether authors provided the relevant information. Next, we scored the studies based on SYRCLE’s risk of bias tool. The presence of bias was scored as “high”, indicating high risk of bias, “low”, indicating low risk of bias, or “unclear,” in case information required to address potential bias was not reported. For the assessment of potential publication bias, we used funnel plots and Egger’s regression analysis^30,31^.

### Statistics and reproducibility

We performed a random effects meta-analysis. For LVEF, infarct size, and fractional shortening, we pooled individual comparisons by calculating the mean difference (MD) with 95% confidence intervals (CIs) between the ECM-treated animals and control groups. For stroke volume and wall thickness, we used standardized mean differences (SMD) because the units of measurements reported differed between studies, and the magnitudes of these values will differ greatly between species^32^. In case multiple experimental groups were present next to one control group within one study, the number of animals in the control group was divided equally by the number of experimental groups^33^. Data are shown as a mean value (MD or SMD) with 95% confidence interval (95% CI), in forest plots and reported in the text as effect size [95%CI].

Because of the exploratory nature of animal studies, we anticipated high between-study heterogeneity for all outcomes and investigated potential sources of heterogeneity through subgroup analysis using (stratified) meta-regression. The predetermined variables for subgroup analysis were species, sex, ECM source, dose of ECM, method of ECM administration, timing to ECM treatment, type of disease model (ischemia/reperfusion injury – IR, or permanent myocardial infarction model – MI), method for the assessment of functional parameters, and timing to outcome measurements (Fig. 2b–h). We considered a meta-regression analysis to be significant at *p* ≤ 0.05. Subgroup analyses were only performed if two or more of the strata contained at least 10 studies, to ensure sufficient power and validity for the analysis. The *p*-values for overall regression analyses of moderators are indicated in the text and Table 1.

Heterogeneity was estimated by using the I^2^ and Chi^2^ statistic; values of 0–25, 25–50, 50–75 and over 75% were considered low, moderate, high, and very high inconsistency, respectively. All analyses were conducted with R 4.2.3. For the meta-analysis and meta-regression itself, we used the metafor 4.2-0 package^34^. To come to an overall conclusion regarding our confidence in the evidence base, we have adapted the GRADE for animals framework according to Hooijmans et al.^35^.

### Reporting summary

Further information on research design is available in the Nature Portfolio Reporting Summary linked to this article.

## Results

### Literature search, screening and study characteristics

**Fig. 1:**
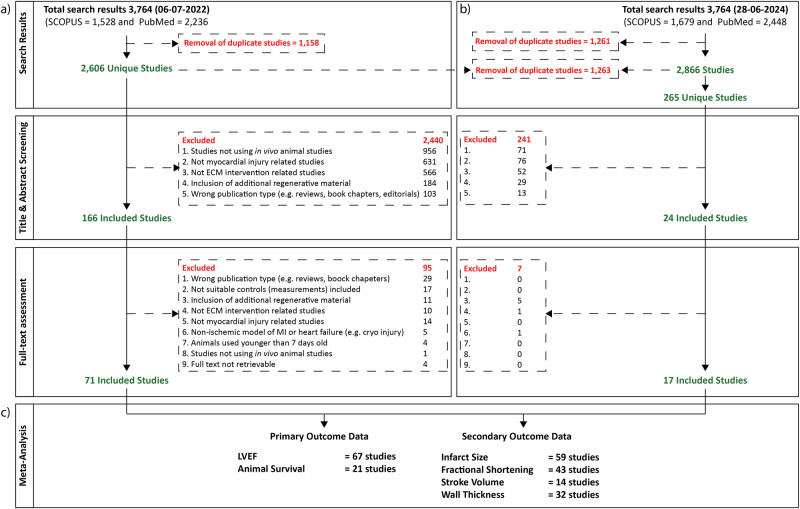
PRISMA flow chart of study selection process. **a** The systematic search in SCOPUS and PubMed of the 6^th^ of July 2022, yielded 2606 unique publications. After title and abstract screening, and full-text assessment 2535 articles were excluded based on the exclusion criteria, and 71 were included. **b** The systematic search in SCOPUS and PubMed of the 28^th^ of June 2024, yielded 265 new unique publications. After title and abstract screening, and full-test screening, an additional 17 studies were included. Data from 88 articles (see Supplementary Reference List) were extracted and included in downstream meta-analysis. **c** Meta-analyses were performed for the effect of ECM treatment on our primary outcomes (Left Ventricular Ejection Fraction – LVEF, 67 studies; Animal Survival, 21 studies) and secondary outcomes (Infarct size, 59 studies; Fractional Shortening, 43 studies; Stroke Volume, 14 studies; Wall Thickness, 32 studies).

Using our bibliographic search strategy (Supplementary Files 3 and 4), in our initial search (6^th^ of July 2022) we identified 1528 articles on SCOPUS and 2236 on PubMed. After removing duplicate entries, we identified 2606 unique articles. After title-abstract screening, 166 articles were selected for full-text screening, of which 71 were finally eligible for meta-analysis (Fig. 1a and Supplementary References). With our search update on the 28^th^ of June 2024, we identified a total of 1679 articles on SCOPUS and 2448 PubMed (Supplementary Files 1 and 2). After the removal of duplicate entries, 2866 articles remained, of which 265 were new compared to our initial search on the 6^th^ of July 2022. After title-abstract screening and full-text screening 17 additional studies were included, making a total of 88 studies eligible for meta-analysis (Fig. 1b, c and Supplementary Reference). Of note, several studies performed more than one experiment, e.g., using different animal/disease models, various doses of the administered ECM, or various compositions of the administered ECM treatment.

**Fig. 2:**
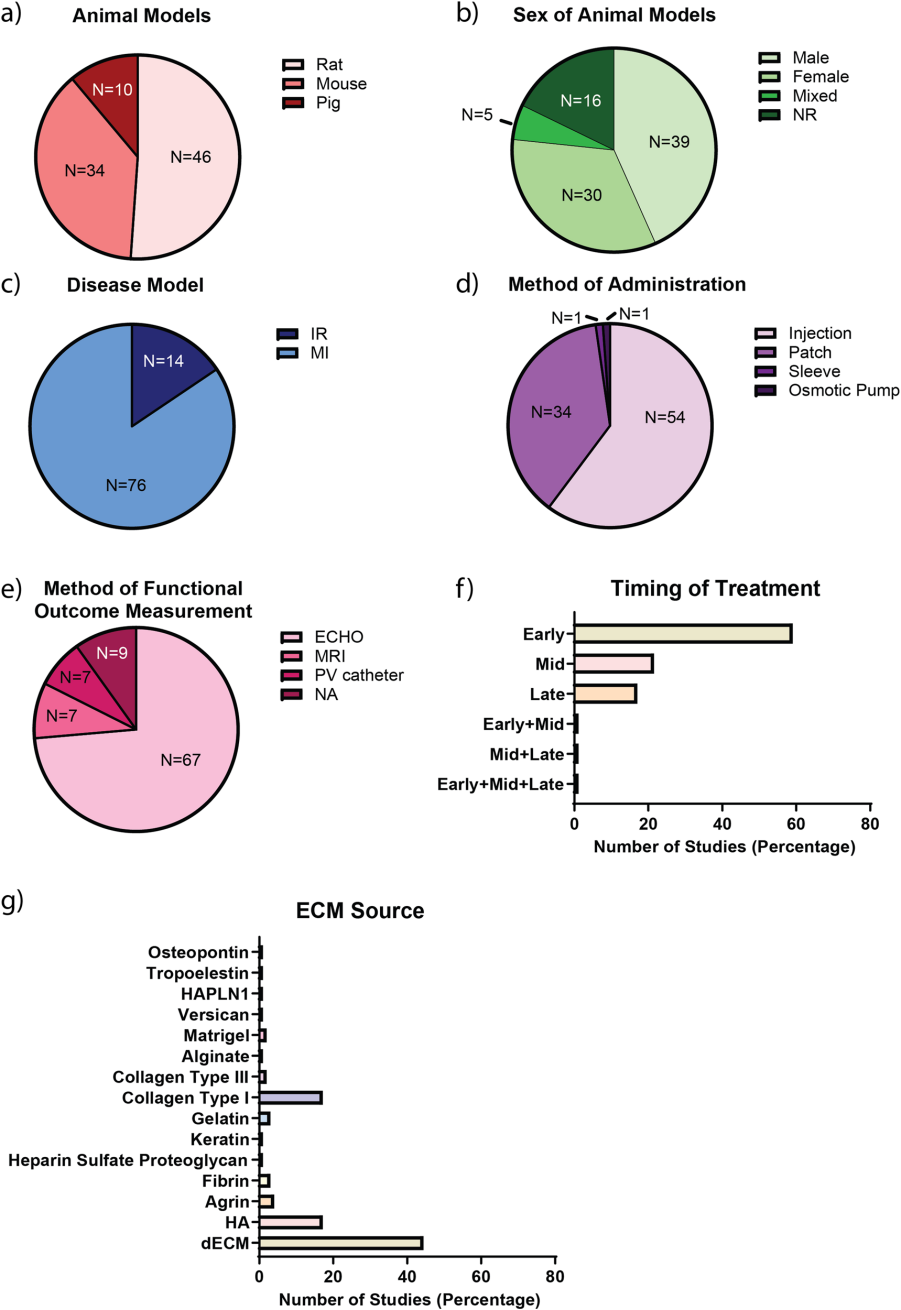
Study characteristics of all included studies. Percent distribution of (**a**) animal models, (**b**) sex of animal models, (**c**) disease models, (**d**) method of administration, (**e**) method of functional outcome measurement, (**f**) timing of treatment, and (**g**) ECM source. For timing of treatment “Early” denotes treatment administration within the 1st day following implementation of infarction, “Mid” treatment within 7 days, and “late” treatment administration after 7 days. N number represents number of studies. ECM extracellular matrix, NR not reported, IR ischemia reperfusion, MI myocardial infarction, ECHO echocardiography, MRI magnetic resonance imaging, PV pressure-volume, NA not applicable, HAPLN1 Hyaluronan And Proteoglycan Link Protein 1, HA hyaluronic acid, dECM decellularized extracellular matrix.

The included studies describe the use of rat (51%), mice (38%), or pig (11%) models (Fig. 2a). Of the 88 studies, 44% used males and 34% females, 5% both sexes and 17% failed to report on sex (Fig. 2b). Studies favored permanent MI models (85%) over ischemia reperfusion models (15%) (Fig. 2c), with ECM being delivered by means of intramyocardial injection (59%), cardiac patch (39%), cardiac sleeve (1%) or osmotic pump (1%) (Fig. 2d). Studies performed cardiac functional measurements, either by echocardiography (75%), MRI (8%), or PV catheter (8%), and 9% did not report how function was measured (Fig. 2e). Interestingly, 59% of the studies administered the ECM-based treatment within the first day post-MI (Fig. 2f), and the most common source of ECM-based treatment was dECM (Fig. 2g). All included studies and study characteristics are listed in Supplementary Data 1.

### Quality of the included studies

**Fig. 3:**
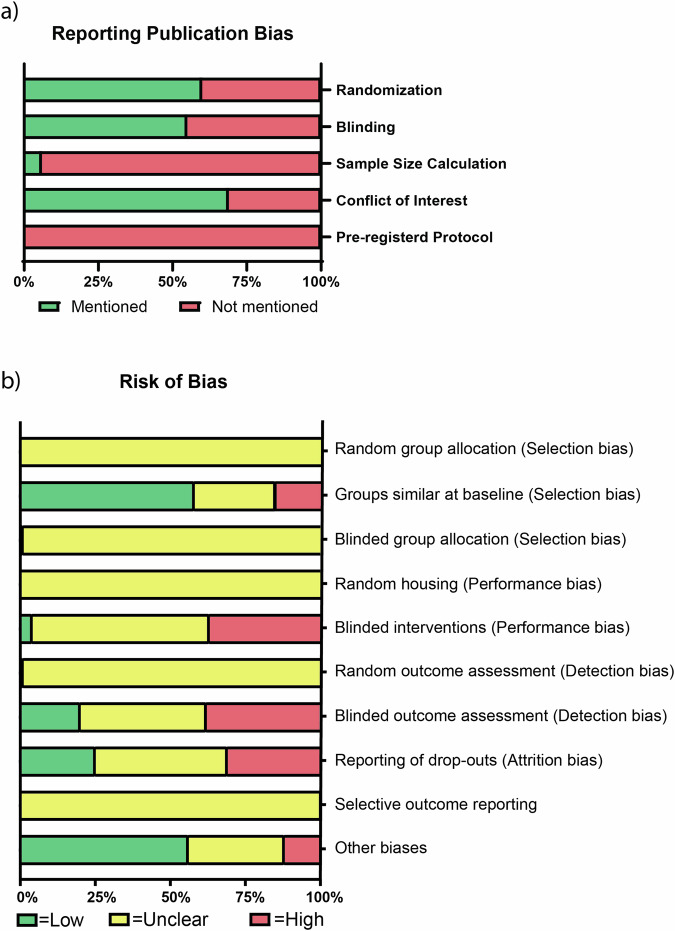
Reporting of key Quality indicators and Risk of Bias. **a** Reporting of five quality indicators in the 88 included studies. 60% of studies reported the use of any form of randomization, 55% reported the use of blinding, and 6% reported a sample size calculation. In addition, 69% of the studies reported a conflict of interest statement, while not a single study reported using a pre-registered study protocol. **b** Using SYRCLE’s risk of bias tool, the risk of selection, performance, detection, attrition and other biases was assessed for the 88 studies included in this systematic review. The proportion of studies, expressed as a percentage, which have a low, unclear, or high risk of bias within the various bias categories.

To determine the quality of the included studies, we first assessed the reporting of any randomization, any blinding, sample size calculation, conflict of interest statement, and pre-registration of study protocol (Fig. 3 and Supplementary Data 2). 60% of studies reported the use of any form of randomization, 55% reported on any form of blinding, and only five studies (6%) reported a sample size calculation. In addition, 69% of the studies reported a conflict-of-interest statement, while not a single study mentioned the pre-registration of the study protocol. Although the majority of authors mentioned applying randomization or blinding in their experiments, only a handful adequately specified the methodology used (Fig. 3a). As such, most studies were assessed to have an unclear risk of bias (Fig. 3b and Supplementary Data 2). Of note, independent of reported blinding, we characterized studies employing patch-based delivery as having a high risk of bias regarding blinding of intervention and outcome assessment as the patch material is visible on the heart regardless of blinding efforts. Additionally, we found that 43% of all studies possessed high attrition bias due to the lack of reporting animal drop-outs and survival throughout the experiments.

### Publication bias of the included studies

To determine whether ECM treatment has a beneficial effect on cardiac functionality post-MI, we performed a random-effects model meta-analysis on our primary outcomes (left ventricular ejection fraction and animal survival) and secondary outcomes (fractional shortening, stroke volume, infarct size, and wall thickness) (Fig. 1). We did not identify a risk of publication bias using visual inspection of the funnel plot and Egger’s regression test for our primary and secondary outcomes, with the exception of wall thickness, which had a significant risk of small-study effects (Supplementary Fig. 1).

### ECM-based treatment improves left ventricular ejection fraction post-MI

**Fig. 4:**
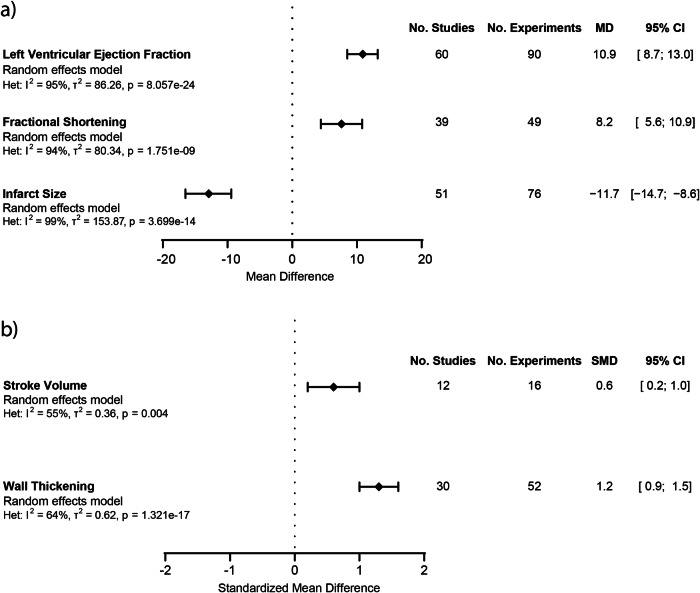
Meta-analysis showing the effect of ECM treatment compared to control. **a** The effect of ECM treatment on left ventricular ejection fraction, fractional shortening, and infarct size, expressed as pooled mean difference (MD) between ECM treatment groups and controls. **b** The effect of ECM treatment on stroke volume and wall thickening, expressed as pooled standardized difference (SMD) between ECM treatment groups compared to controls. Error bars represent the 95% confidence interval (CI). Data were pooled using a random effects model. No. Studies is the number of included studies that described the outcome data and were included in the meta-analysis. No. Experiments is the number of specific experiments described within the studies of which outcome data were included in the meta-analysis.

**Fig. 5:**
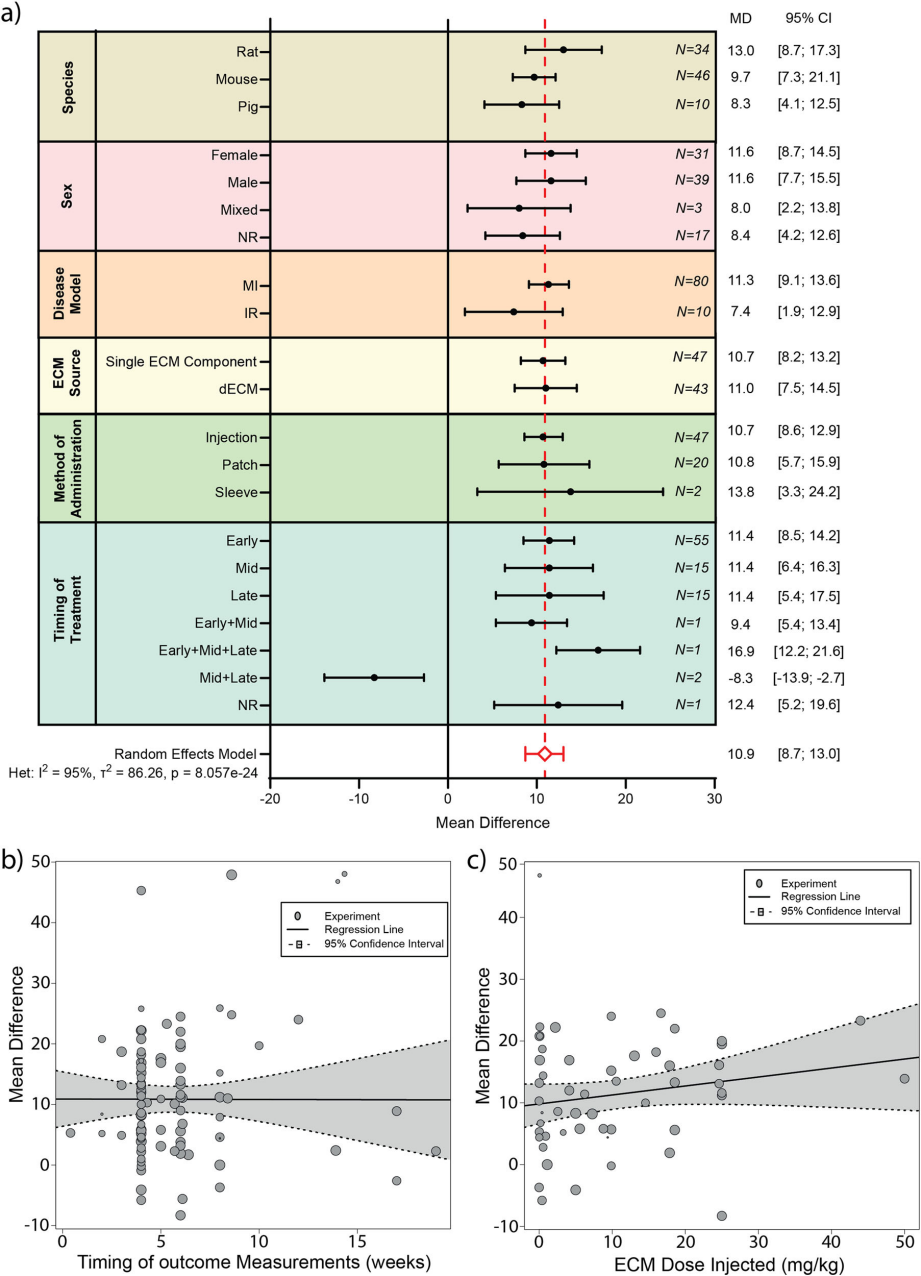
Subgroup analysis of various influencing factors on LVEF following ECM treatment. **a** Subgroup analysis showing pooled estimates (black symbols) per stratum for the variables species, sex, disease model, ECM source, method of ECM administration and timing of ECM treatment [Early (< 1day post-MI), Mid ( = 1-7days post-MI), Late (> 7days post-MI)]. The overall pooled mean difference (MD) from a random effects model, is shown by a red dotted line and a diamond (respectively). Error bars represent 95% confidence interval (CI). N represents the number of experiments within each subgroup. **b** Meta-regression timing of outcome measurement and **(c**) ECM dose. NR not reported, MI myocardial infarction, IR ischemia reperfusion, ECM extracellular matrix, dECM decellularized extracellular matrix.

To determine the impact of ECM-based treatment post-MI to preserve cardiac function, we assessed left ventricular ejection fraction (LVEF), the golden standard for evaluating cardiac function, throughout the included studies^2^. Of the 88 studies that were included, 67 reported on LVEF. Seven of these were excluded from the meta-analysis since the reported data was incomplete^36–42^. We observed an improvement in LVEF of 10.9% [8.7%;13.0%] at follow-up after ECM treatment (*N* = 745 animals), when compared to the control treatment group (*N* = 547 animals), with significant heterogeneity (*p* = 8.057e-24) and a high inconsistency (I^2^ = 95%) (Fig. 4a and Supplementary Fig. 2). We performed subgroup analysis on various factors to assess their effect on LVEF (Fig. 5 and Table 1). Interestingly, none of the assessed factors were shown to explain the heterogeneity observed in the LVEF meta-analysis.

**Table 1:** Summary of Subgroup analysis

	Outcome											
Variable	LVEF		Animal Survival		Fractional Shortening		Stroke Volume		Infarct Size		Wall Thickening	
	R^2^	*p* value	R^2^	*p* value	R^2^	*p* value	R^2^	*p* value	R^2^	*p* value	R^2^	*p* value
Species	1.52%	0.30	–	–	12.08%	0.03	–	–	3.49%	0.13	0.00%	0.42
Sex	0.00%	0.68	–	–	0.00%	0.58	–	–	6.99%	0.06	0.00%	0.46
ECM Source	3.88%	0.95	–	–	0.37%	0.30	–	–	0.00%	0.72	0.00%	0.49
ECM Administration	0.00%	0.91	–	–	3.78%	0.12	–	–	4.11%	0.05	6.52%	0.04
Disease Model	–	–	–	–	–	–	–	–	–	–	0.60%	0.09
Timing of ECM treatment	0.00%	0.59	–	–	–	–	–	–	0.00%	0.86	2.20%	0.21
Method for functional assessment	–	–	–	–	–	–	–	–	–	–	–	–
ECM Dose	3.04%	0.16	–	–	0.00%	0.76	–	–	0.00%	0.44	1.40%	0.14
Timing to outcome measurements	0.00%	0.99	–	–	5.17%	0.09	–	–	0.00%	0.77	0.00%	0.96

R^2^ = amount of heterogeneity accounted for.

– = missing data and/or subgroup group sizes <10.

*ECM* extracellular matrix, *LVEF* left ventricular ejection fraction, *CI* confidence interval.

### ECM-based treatment does not seem to influence animal survival post-MI

As our second primary outcome, we investigated the effect of ECM treatment on animal survival. Interestingly, of the 88 studies included, only 14 reported on animal survival. Of these 14, only 6 studies reported incidence of animal death, while all remaining studies did not report dropout of animals (in either both the ECM treated and control, or in one of these groups). Making it impossible to perform a meta-analysis on the risk ratio for animal survival. Cumulatively, out of the total animals treated with ECM (*N* = 176), 6.8% died, while out of the control-treated animals (*N* = 151), 8.6% were reported to have died (Supplementary Data 4).

### ECM-based treatment improves fractional shortening post-MI

Another commonly used parameter to assess cardiac function is fractional shortening (FS). We, therefore, performed a meta-analysis on the effect of ECM treatment post-MI on FS. Of the 88 studies included, 43 reported on FS. Four additional studies were excluded from the meta-analysis due to incomplete data reporting^37,43–45^. ECM treatment (*N* = 379 animals) led to an improvement of 8.2% [5.6%; 10.9%] in FS, when compared to the control treatment (*N* = 329 animals), with significant heterogeneity (*p* = 1.751e-09) and very high inconsistency (I^2^ = 94%) (Fig. 4a and Supplementary Fig. 3).

**Fig. 6:**
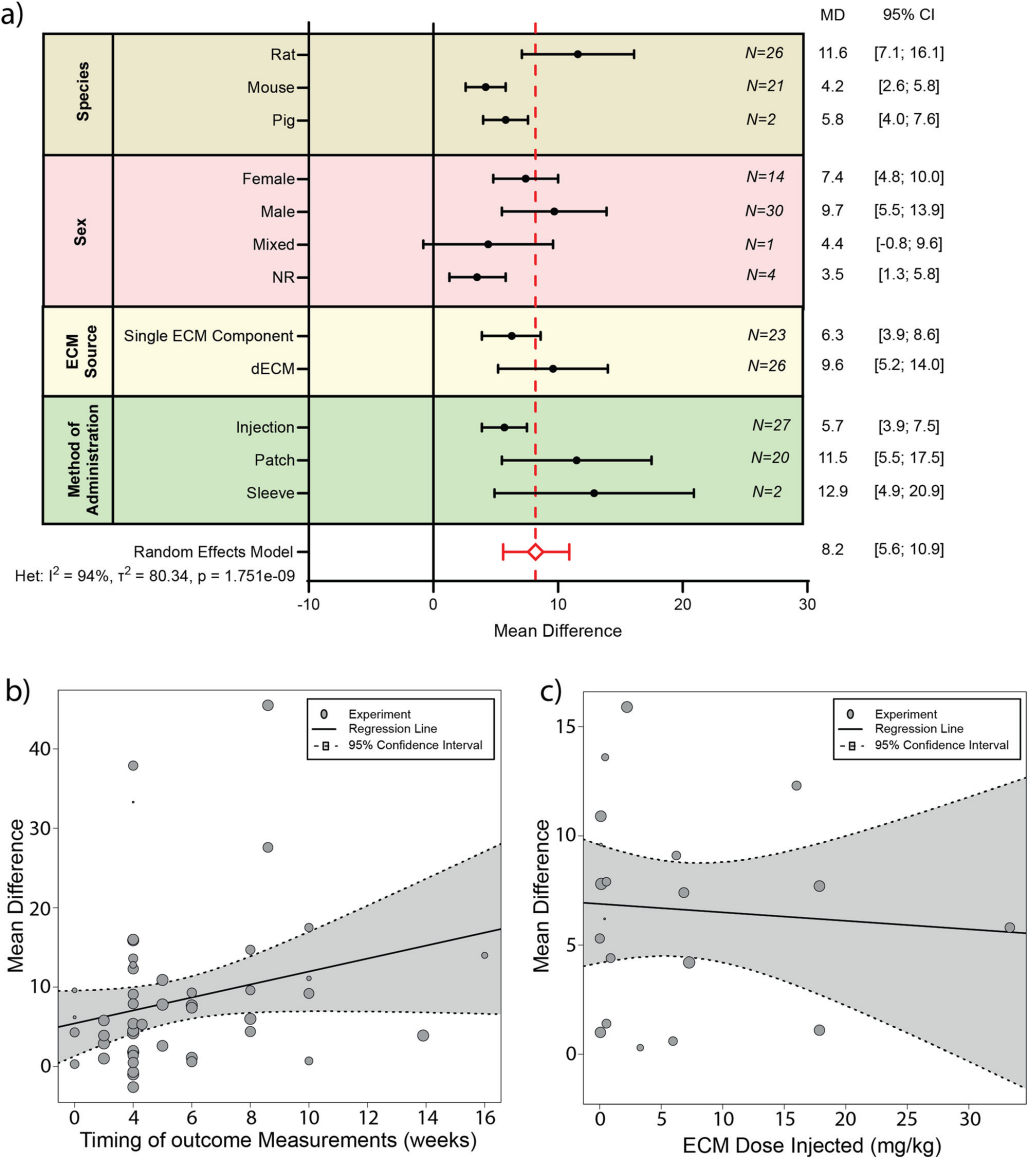
Subgroup analysis of various influencing factors on Fractional Shortening following ECM treatment. **a** Subgroup analysis showing pooled estimates (black symbols) per stratum for the variables species, sex, ECM source, method of ECM administration. The overall pooled mean difference (MD) from a random effects model, is shown by a red dotted line and a diamond (respectively). Error bars represent 95% confidence interval (CI). N value represents the number of experiments within each subgroup. **b** Meta-regression for timing of outcome measurement. **c** Meta-regression for ECM dose. NR not reported, ECM extracellular matrix, dECM decellularized extracellular matrix.

To determine whether specific factors could have an influence on the observed effect, we performed various subgroup analyses (Fig. 6 and Table 1). Regression analysis on the factor species demonstrated a significant effect (*p* = 0.03), with rat studies showing a larger improvement in FS as compared to mice (MD 11.6% [7.1%;16.1%] versus 4.2% [2.6%;5,8%], respectively). Besides species, none of the other assessed factors were shown to explain the heterogeneity observed in the FS meta-analysis.

### ECM-based treatment improves stroke volume post-MI

To further characterize the effect of ECM treatment post-MI on cardiac functionality, we performed a meta-analysis on the effect of ECM treatment on stroke volume. Only 12 studies of the 88 included reported on left ventricular stroke volume. Since stroke volume is not reported in the same unit throughout the studies, and since the magnitudes of these values and standard errors differed greatly across species, we performed a meta-analysis utilizing the SMD. We found a significant beneficial effect on stroke volume following the administration of ECM (*N* = 132 animals), when compared to control treatments (*N* = 93 animals), in MI models (SMD 0.6, [0.2;1.0]), with significant heterogeneity (*p* = 0.004) and high inconsistency (I^2^ = 63%) (Fig. 4b and Supplementary Fig. 4). Since only 10 studies reported on stroke volume, we were not able to perform any subgroup analysis to test for the effects of different influencing factors.

### ECM-based treatment reduces infarct size post-MI

Besides characterizing the functional effects of ECM treatment post-MI, we were also interested in determining what the effects were on the tissue level. Infarct size, as a surrogate of myocardial injury, was therefore assessed as a measure for tissue homeostasis post-MI^46^. Within the included studies, 60 reported outcome data on infarct size. Nine additional studies were excluded due to incomplete infarct size data reporting^36,37,43,47–52^. Of the remaining 51 studies we performed a meta-analysis to assess the effect of ECM treatment post-MI on infarct size. The performed random-effects meta-analysis demonstrated that treatment with ECM (*N* = 504 animals) following MI, led to a reduction in infarct size of 11.7% [−14.7%; −8.6%], when compared to the control treatment (*N* = 362 animals), with significant heterogeneity (*p* = 3.699e-14) and very high inconsistency (I^2^ = 99%) (Fig. 4a and Supplementary Fig. 5).

**Fig. 7:**
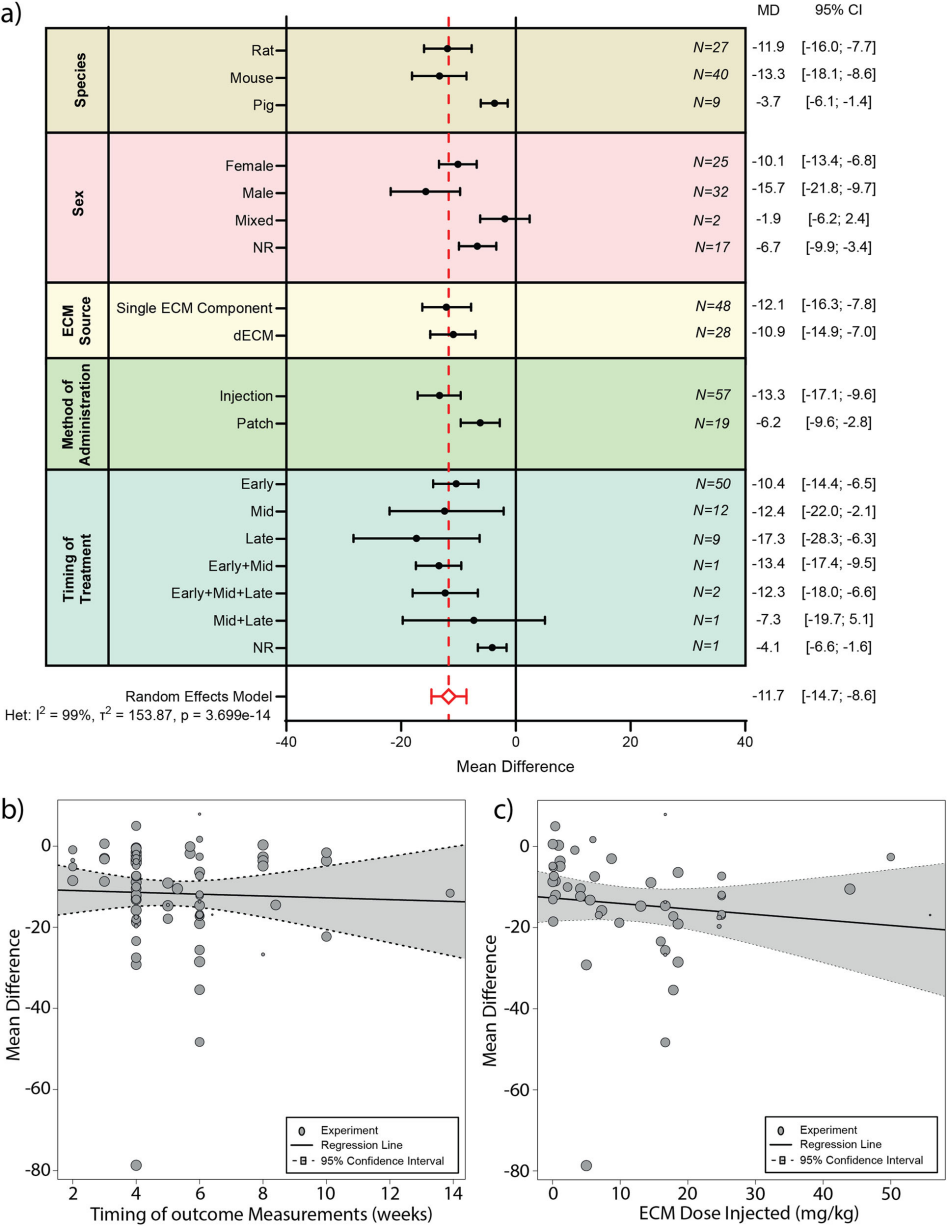
Subgroup analysis of various influencing factors on Infarct Size following ECM treatment. **a** Subgroup analysis showing pooled estimates (black symbols) per stratum for the variables species, sex, ECM source, method of ECM administration and timing of ECM treatment [Early (< 1day post-MI), Mid (= 1–7days post-MI), Late (>7days post-MI)]. The overall pooled mean difference (MD) from a random effects model, is shown by a red dotted line and a diamond (respectively). Error bars represent 95% confidence interval (CI). N represents the number of experiments within each subgroup. Meta-regression for timing of outcome measurement (**b**) and ECM dose **(c**). NR not reported, ECM extracellular matrix, dECM decellularized extracellular matrix.

To determine whether specific factors could have an influence on the observed effect, we performed subgroup analysis (Fig. 7 and Table 1). We found that studies that administered ECM treatment by means of an intramyocardial injection had more than twice the reduction in infarct size, when compared to studies with ECM administered in the form of a patch (MD −13.3% [−17.1%; −9.6%] VS MD −6.2, [−9.6%; −2.8%], respectively, *p* = 0.05). Besides ECM administration, none of the other assessed factors were shown to explain the heterogeneity observed in the infarct size meta-analysis.

### ECM-based treatment increases wall thickening post-MI

Post-MI remodeling is exemplified by left ventricular wall thinning and ventricular dilatation^53^. We thus assessed ECM treatment effects on left ventricular wall thickness. Of the included 88 studies, 32 studies reported on wall thickness. Two studies were excluded due to incomplete wall thickness reporting^37,54^. Similar to stroke volume, the magnitudes of the wall thickness values and standard errors differ greatly across species and studies (i.e., mm, µm, %, mm2). We, therefore, performed a meta-analysis utilizing the SMD on the remaining 30 studies to determine the effect of ECM treatment on LV wall thickness following MI. The meta-analysis described a beneficial effect towards increasing LV wall thickness following the administration of ECM (*N* = 414 animals) compared to control treatment (*N* = 344 animals) (SMD 1.2, [0.9; 1.5], with significant heterogeneity (*p* = 1.32e−17) and high inconsistency (I^2^) of 64% (Fig. 4b and Supplementary Fig. 6).

Our subgroup analysis indicates that the mode of ECM administration modified the efficacy of ECM treatment on left ventricular wall thickness (regression analysis *p* = 0.04). Administrating ECM by means of an intramyocardial injection appeared to perform better than the patch treatment strategy (SMD 1.4 [1.1; 1.8] and SMD 0.8 [0.4; 1.2], respectively). No additional factors were found to explain the heterogeneity observed in the wall thickness meta-analysis.

**Fig. 8:**
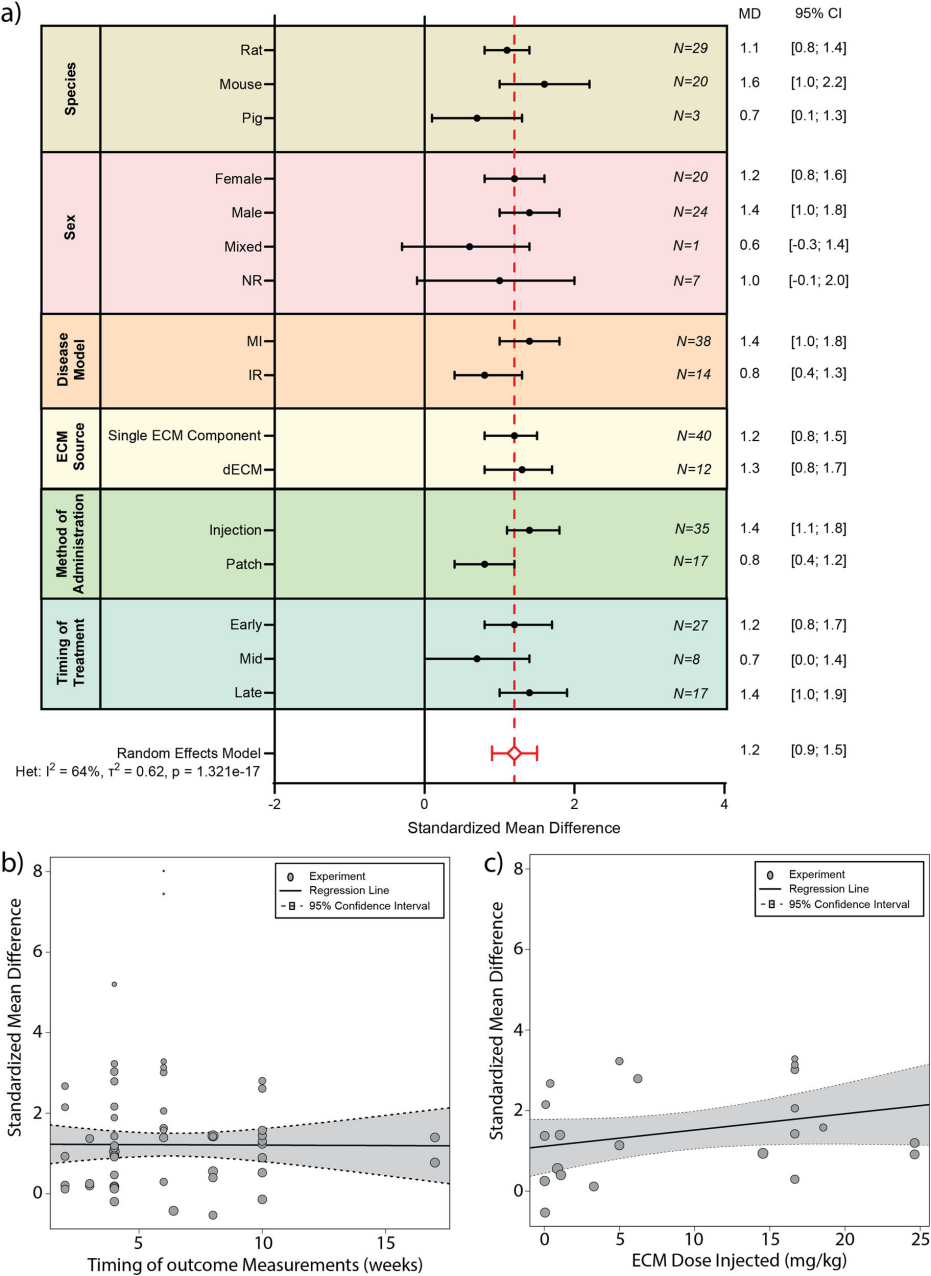
Subgroup analysis of various influencing factors on Wall Thickening following ECM treatment. **a** Subgroup analysis showing pooled estimates (black symbols) per stratum for the variables species, sex, disease model, ECM source, method of ECM administration, and timing of ECM treatment [Early (<1day post-MI), Mid (= 1-7days post-MI), Late (>7days post-MI)]. The overall pooled standardized mean difference (SMD) from a random effects model, is shown by a red dotted line and a diamond (respectively). Error bars represent 95% confidence interval (CI). N represents the number of experiments within each subgroup. Meta-regression for timing of outcome measurement (**b**) and ECM dose (**c**). NR not reported, MI myocardial infarction, IR ischemia reperfusion, ECM extracellular matrix, dECM decellularized extracellular matrix.

### Decellularized ECM is the most commonly used source for ECM-based treatment strategies

**Fig. 9:**
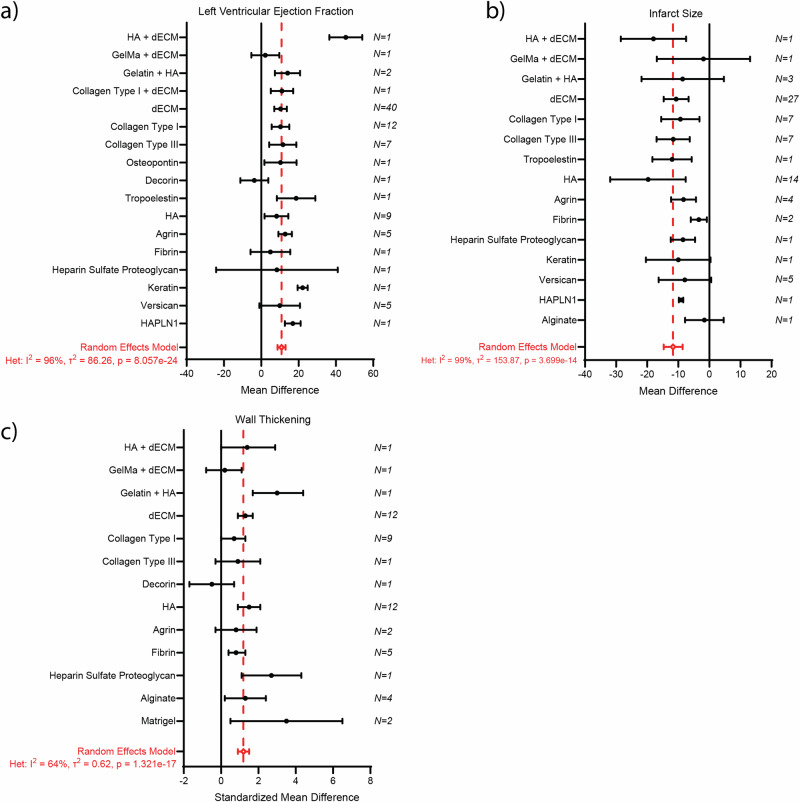
Subgroup analysis for ECM source (individual components). Subgroup analysis for individual ECM components effect on left ventricular ejection fraction (**a**) Infarct size (**b**) and wall thickness (**c**). For each ECM component, black symbols represent the mean difference (MD) or standard mean difference (SMD). The overall pooled MD or SMD from a random effects model is shown by red dotted line and diamond (respectively). Error bars represent the 95% confidence interval (CI). N is the number of independent comparisons (studies) within each subgroup. ECM extracellular matrix, HA hyaluronic acid, dECM decullarized extracellular matrix, HAPLN1 Hyaluronan And Proteoglycan Link Protein 1.

Within all our outcome parameters, the subgroup analysis for ECM source did not identify a significant difference between dECM and single ECM components. Since we were interested in identifying whether there is an ideal ECM source to be used as a treatment strategy post-MI, we performed an additional subgroup analysis for all outcome measurements where we individually grouped all ECM components (with the exception of FS, due to insufficient data; Fig. 9). In total, 19 different ECM-based treatments (e.g., dECM, Collagen Type I, Collagen Type III, Agrin, Fibrin, HA, Versican) were used, with dECM being the most common. We observed no significant effect on the various ECM components for LVEF (0.30) or infarct size (*p* = 0.85). Interestingly, for wall thickening, a significant effect was observed (*p* = 0.04). This suggests a potential difference regarding the ECM component used on improvement on wall thickening.

## Discussion

Performing extensive meta-analysis of published data can massively benefit evidence-based decisions in healthcare^27^. By investigating the effect of ECM treatment, independent of study-specific methodology and animal model used, we report the relevance of ECM-based therapy to treat MI in the pre-clinical setting by determining pooled effects on several clinical outcome parameters (e.g., LVEF, FS, stroke volume, wall thickness and infarct size). Previously, systematic reviews on the effect of stem cell treatment on cardiac function in animal models post-MI, described an improvement in LVEF of 7.5-10.7% when compared to control treatment^46,55–57^. The effect of stem cell treatment on pre-clinical models has also been shown to reduce infarct size by 10.9%, increase fractional shortening (MD 6.0%), and left ventricular wall thickening (SMD 1.7) when compared to control treatment^55^. Our analysis of 88 published pre-clinical studies, suggest ECM-based therapies, have comparable, if not larger, beneficial effects (improvement of LVEF by 10.9%, fractional shortening by 8.2%, wall thickness by SMD 1.2, stroke volume by SMD 0.6, and reduce infarct size by 11.7%) compared to stem cell treatments. This could suggest that besides paracrine effects, as is the case with cell-based approaches^58,59^, ECM-based treatment strategies directly affect tissue homeostasis, resulting in an improvement in cardiac function. Recent systematic reviews have also aimed at characterizing the effects of therapies utilizing various hydrogels (i.e., synthetic hydrogels or ECM-based hydrogels) in combination with additional regenerative material (i.e. stem cells, cytokines, drugs, extracellular vesicles)^60,61^. Interestingly, the effect of combination therapies, especially combined stem cell and hydrogel-based therapies, either synthetic or ECM-derived, significantly improved LVEF by 8.0-16.5% and FS by 5.5-6.3% when compared to stem cell or hydrogel-based treatment strategies alone^60,61^. This suggests that tackling MI injury not only from a cellular aspect but also from an ECM level may prove to be the next step forward in cardiac regenerative therapies.

Regarding the external validity of studies in the field, we found that the effects of ECM therapy were highly reproducible across sex and various species in terms of direction and magnitude of effects, representing promising results for clinical application. However, to move towards ECM-based therapy to treat patients post-MI, other important external validity factors need to be taken into account: i) method of administration, ii) timing of the treatment, iii) ECM source, and iv) ECM dosage.

For the former, we show that intramyocardial injection of ECM-based therapy has higher therapeutic benefit in terms of cardiac function and scar size than the placement of patches. This would be advantageous for clinical translation because intramyocardial injection would require a much less invasive delivery approach compared to a thoracotomy for patch placement^62^. Interestingly, we did not find dependence on the timing of treatment (early = <1 day, mid = 1–7 days, and late = >7 days). When compared to cell-based therapies, it has been observed that the effect of stem cell treatment is more pronounced when applied after 7 days post-MI^56,63,64^. It has been suggested that due to a hostile microenvironment in the acute phase (>1 day) post-MI, cellular retention and survival is reduced, greatly affecting the efficacy of stem cell-based treatments^56^. Combined, this could suggest that, unlike cell-based treatment strategies, the timing of treatment initiation with ECM-based treatment strategies seems to have a larger therapeutic window, which could be a great benefit when thinking of clinical translation. This observation is supported by a recent first-in-man clinical trial of VentriGel (an ECM-based strategy derived from decellularized porcine myocardium) that used intramyocardial injections in patients, 60 days to 3 years post-MI, which showed increases in 6-min walk test distance and decreases in New York Heart Association functional class across the entire cohort of patients^65^.

Additionally, with exception of wall thickening, we did not observe any significant differences between dECM and the pooled single ECM components subgroup, suggesting both are equally effective in improving outcome when administered following an MI (when compared to control injection, i.e. saline or PBS). However, we must keep in mind that, besides dECM, which was the most commonly used form of ECM-based therapy, there were not enough studies looking at individual single ECM components (i.e. fibrin, agrin, or versican). Additionally, we could not identify a clear link between ECM dose and the outcome parameters. In terms of inconsistency, meta-analysis revealed high heterogeneity for all outcomes which our prespecified subgrouping variables could not (fully) explain; this decreases our confidence in the evidence.

Since we do not observe a difference between the various types of ECM and the dosage of ECM, this could also suggest that the beneficial effect of ECM-based treatment does not necessarily rely on downstream biochemical signalling or paracrine effects, resulting from ECM-cell interactions but rather on modulating the mechanical properties of the surrounding tissue. It is well established that following an MI, the heart undergoes a process of cardiac remodelling which results in the formation of an infarct, ventricular wall thickening, and eventually a diminished cardiac muscle functionality^8^. Although the infarcted tissue can preserve structural integrity of the post-MI heart, the increased collagen composition and stiffness, results in an inhospitable microenvironment for cardiac cells^66,67^. By supplying a “healthy” ECM, as is the case in the studies included in this systematic review, a hospitable micro environment is supplied, which could foster cell survival and therefore reduce infarct size and improve cardiac function. Our findings are supported by recent clinical studies aimed at testing the feasibility and safety of ECM-based strategies in the human cardiovascular setting^65,68,69^. Although, improvement in patient outcome was not a primary goal for these studies, they have demonstrated an improvement in cardiac function and patient well-being following ECM-based treatment^65,68,69^.

When assessing the reporting quality and internal validity of the included studies, akin to previous pre-clinical systematic reviews, we demonstrate that studies in the field of ECM-based therapy in the cardiovascular setting are incompletely reported and at unclear risk of several types of bias^70,71^. Half of the included studies mentioned randomization or blinding of their experiments. However, they fail to provide the details necessary to assess whether or not randomization/blinding was actually achieved and at which phase of the experiment. The small number of details provided on various aspects of experimental design resulted in most of the risk of bias items being assessed as unclear. Additionally, the included studies did not justify group sizes by means of, e.g., a sample size calculation, even though this is a key element in experimental design and is obligatory for approval by most animal ethics committees. Reporting the sample size calculation, including the expected effect size, prevents changing the primary and secondary outcomes based on the study results, reducing the risk of bias due to selective outcome reporting^72^. The quality of a study is further brought into doubt when both sample size calculations and animal survival and drop-outs are not appropriately reported, as is the case with the majority of our included studies, severely hampering the reproducibility and impact of the results presented by the studies.

The Grading of Recommendations, Assessment, Development, and Evaluation (GRADE) is the most widely used framework to rate the certainty in the evidence and strength of health care recommendations^35,73^. To summarize our confidence in the evidence discussed in this systematic review, we applied the draft pre-clinical GRADE approach to all outcomes (Table 2)^35^. Based on the risk of bias, inconsistency, indirectness, imprecision, and risk of publication bias, we can conclude that data related to the LVEF, FS, and infarct size has a moderate quality of evidence. This suggests that further research is likely to have an important impact on our confidence in the estimate of effect and could possibly change the estimate^73^. For wall thickness, we conclude the data to have a low quality of evidence, suggesting additional research will have an important impact on our confidence in the estimate of the effect and is likely to change the estimate^73^. Finally, based on the draft pre-clinical GRADE framework, our data regarding stroke volume has a very low quality of evidence, suggesting that the observed effect is very uncertain^73^. Taken together, we cannot yet formulate a clear recommendation for future clinical trials. However, the results generated throughout this systematic review can be used to stimulate future research directions geared towards the standardization of pre-clinical studies focusing on unravelling the role of ECM-based treatment strategies post-MI.

**Table 2:** Summary of Findings

Outcomes	No. of studies (animals)	Pooled effect [95% CI]	Risk of Bias	Inconsistency	Indirectness	Imprecision	Publication Bias	Certainty of the evidence (GRADE)
**LVEF**	60 studies (1292 animals)	MD 10.9% [8.7%; 13.0%]	Downgrade	Downgrade	No Effect	No Effect	No Effect	⊕⊕⊕○ Moderate^a^
**FS**	38 studies (708 animals)	MD 8.2% [5.6%; 10.9%]	Downgrade	Downgrade	No Effect	No Effect	No Effect	⊕⊕⊕○ Moderate^a^
**Stroke Volume**	12 studies (225 animals)	SMD 0.6 [0.2; 1.0]	Downgrade	Downgrade	No Effect	Downgrade	No Effect	⊕○○○ Very Low^b^
**Infarct Size**	52 studies (866 animals)	MD -11.7% [-14.7%; -8.6%]	Downgrade	Downgrade	No Effect	No Effect	No Effect	⊕⊕⊕○ Moderate^a^
**Wall Thickness**	30 studies (758 animals)	SMD 1.2 [0.9; 1.5]	Downgrade	Downgrade	No Effect	No Effect	Downgrade	⊕⊕○○ Low^c^

^a^Moderate quality of evidence is due to unknown/unclear risk of bias and serious inconsistency.

^b^Low quality of evidence is due to unknown/unclear risk of bias, serious inconsistency and imprecision.

^c^Low quality of evidence is due to unknown/unclear risk of bias, serious inconsistency and publication bias.

*LVEF* left ventricular ejection fraction, *FS* fractional shortening, *MD* mean difference, *SMD* standardized mean difference, *CI* confidence interval.

Taken together we propose future research to focus on dissecting the exact composition of the dECM used in these ECM-based therapies to help identify specific ECM components which could be key to promote cardiac improvement on the tissue and function level post-MI^8,74^, while also invest in longer studies to characterize the long-term effects of the ECM therapy for myocardial infarction. Besides improving our understanding of ECM-based treatment strategies, the pre-clinical studies should also start to adhere to similar standards of reporting as clinical studies, to ensure better reporting, reproducibility, and certainty in the evidence. To date, this has not been the case, and the quality of reporting animal studies has lagged behind that of human randomized controlled trials. The correct reporting of human randomized controlled trials has only started to be enforced since the introduction of the CONSORT (Consolidated Standards of Reporting Trials) statement in 1996, by improving trail design, accounting of subjects, and rigour of data analysis^75–78^. By contrast, guidelines for conduct and scientific reporting of animal studies were introduced in 2010, with the ARRIVE (Animal Research: Reporting In Vivo Experiments) guidelines^75,79^. Since then, numerous journals have started to adhere to the ARRIVE guidelines. However, to further improve the overall reliability and unbiased nature of pre-clinical animal research, as with human randomized controlled trials, we would like to stress the need for preregistration of study protocols. Although still in its infancy, enforcing pre-registration of animal study protocols will greatly improve transparency and standardization, while also reducing duplication and publication bias across pre-clinical studies^80^.

## Supplementary information

Supplementary Information

Description of Additional Supplementary Files

Supplementary Data 1-8

reporting summary

## Data Availability

The authors declare that the data supporting the findings of this study are available within the paper and its supplementary information files. The source data for Fig. 1 – PRISMA flow chart, can be found in Supplementary File 1–4 and Supplementary References. The source data for Fig. 2 – study characteristics, can be found in Supplementary Data 1. The source data for Fig. 3 – reporting and risk of bias can be found in Supplementary Data 2. The source data for Fig. 4 – pooled meta-analysis, can be found in Supplementary Data 3 and 5–8. The source data for Fig. 5 – LVEF subgroup analysis and Supplementary Fig. 2 – LVEF forest plot, can be found in Supplementary Data 3. The source data for Fig. 6 – fractional shortening subgroup analysis and Supplementary Fig. 3 – fractional shortening forest plot can be found in Supplementary Data 5. The source data for Supplementary Fig. 4 – stroke volume forest plot, can be found in Supplementary Data 6. The source data for Fig. 7 – infarct size subgroup analysis and Supplementary Fig. 5 – infarct size forest plot, can be found in Supplementary Data 7. The source data for Fig. 8 – wall thickening subgroup analysis and Supplementary Fig. 6 can be found in Supplementary Data 8. The source data for Fig. 9 – ECM source subgroup analysis, can be found in Supplementary Data 3, 7, and 8. Source data for Table 1 can be found in Supplementary Data 3, 5, and 7–8. Source data for Table 2 can be found in Supplementary Data 1–8.
