# Supplementary material for: Preclinical extracellular matrix-based treatment strategies for myocardial infarction: a systematic review and meta-analysis
Source: Commun Med (Lond). 2025 Mar 30;5:95. doi: 10.1038/s43856-025-00812-y (PMC11955565; doi:10.1038/s43856-025-00812-y)
Supplement: Supplementary file 1 — Supplementary Information [file 43856_2025_812_MOESM1_ESM.pdf]

Supplementary Information

Preclinical extracellular matrix-based treatment strategies for myocardial infarction: a systematic review and meta-analysis

Atze van der Pol[1,2*], Marijn C. Peters[1,5], Ignasi Jorba[1,2,3], Anke M. Smits[4], Niels P. van der Kaaij[5], Marie-Jose Goumans[4], Kimberley E. Wever[6], Carlijn V.C. Bouten[1,2*]

1. Soft Tissue Engineering and Mechanobiology, Department of Biomedical Engineering, Eindhoven University of Technology, Eindhoven, The Netherlands
2. Institute for Complex Molecular Systems, Eindhoven University of Technology, Eindhoven, The Netherlands
3. Unitat de Biofísica i Bioenginyeria, Facultat de Medicina i Ciències de la Salut, Universitat de Barcelona, 08036 Barcelona, Spain
4. Department of Cell and Chemical Biology, Leiden University Medical Center, Leiden, The Netherlands
5. Department of Cardiothoracic Surgery, Regenerative Medicine Centre, University Medical Center Utrecht, Utrecht, The Netherlands
6. Department of Anesthesiology, Pain and Palliative Medicine, Radboud University Medical Center, Nijmegen, Gelderland, The Netherlands

***Corresponding author: Atze van der Pol (a.v.d.pol1@tue.nl) and Carlijn Bouten (C.V.C.Bouten@tue.nl)**

Supplementary Figures

Supplementary Figure 1.

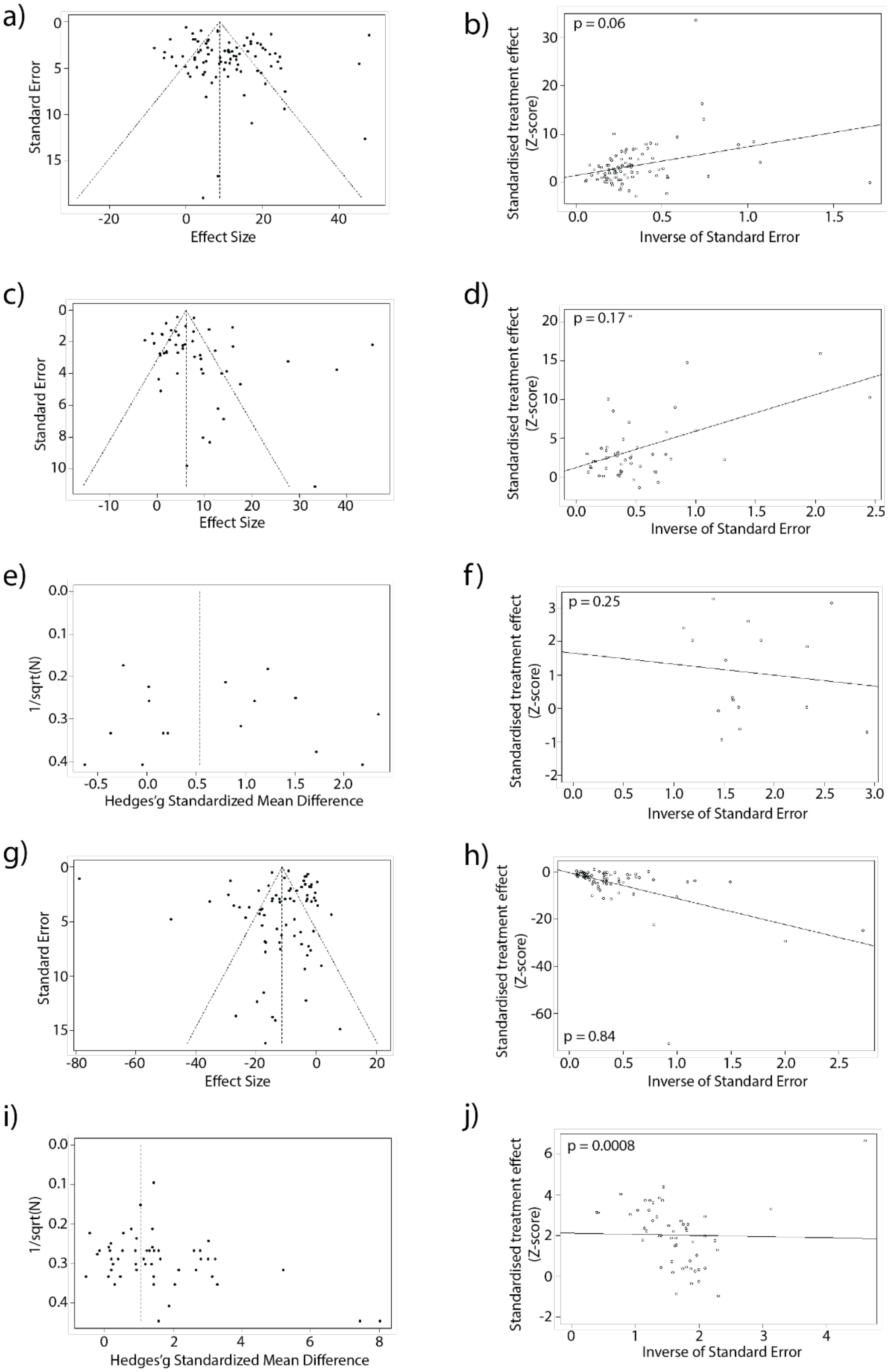

Publication Bias assessment for all outcomes included in meta-analysis . a-b) Funnel plot **(a)** and Egger's regression **(b)** for left ventricular ejection fraction (LVEF). **c-d).** Funnel

plot **(c)** and Egger's regression **(d)** for fractional shortening. **e-f)** Funnel plot of the Hedges'g Standardized Mean Difference **(e)** and Egger's regression **(f)** for stroke volume. **g-h)** Funnel plot **(g)** and Egger's regression **(h)** for infarct size. **i-j)** Funnel plot using the Hedges'g Standardized Mean Difference **(i)** and Egger's regression **(j)** for wall thickening. For funnels plots, asymmetry around the pooled mean difference or standardized mean difference (dotted lines) indicates a risk of publication bias. For Egger's regression, a p-value below 0.05 indicates a risk of publication bias.

Supplementary Figure 2.

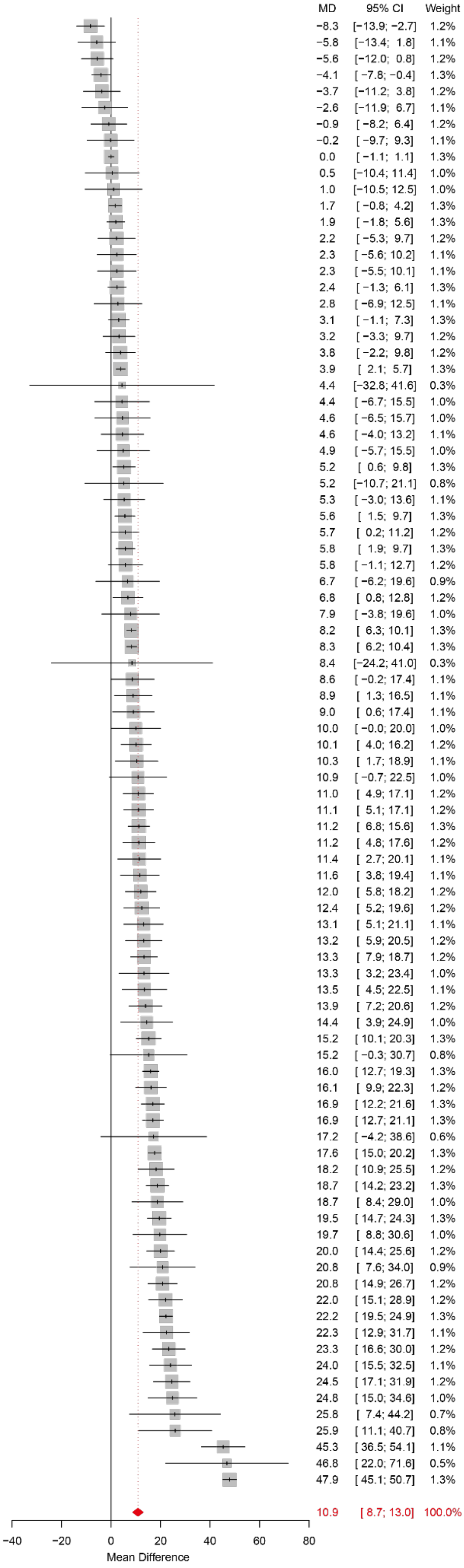

Study	MD	95% CI	Weight
Pupkaite J, 2020 Exp. 3	−8.3	[−13.9; −2.7]	1.2%
Feng J, 2024 Exp. 5	−5.8	[−13.4; 1.8]	1.1%
Neuta P, 2015 Exp. 2	−5.6	[−12.0; 0.8]	1.2%
Wang N, 2019 Exp. 1	−4.1	[−7.8; −0.4]	1.3%
Mohindra P, 2023	−3.7	[−11.2; 3.8]	1.2%
Svystonyuk D, 2020 Exp. 2	−2.6	[−11.9; 6.7]	1.1%
Hao Y, 2022	−0.9	[−8.2; 6.4]	1.2%
Blackburn N, 2015 Exp. 3	−0.2	[−9.7; 9.3]	1.1%
Chang M, 2016	0.0	[−1.1; 1.1]	1.3%
Nair RS, 2023	0.5	[−10.4; 11.4]	1.0%
Wang Q, 2016	1.0	[−10.5; 12.5]	1.0%
Cortes−Morichetti M, 2007	1.7	[−0.8; 4.2]	1.3%
Wang Z, 2019 Exp. 2	1.9	[−1.8; 5.6]	1.3%
Gao L, 2017	2.2	[−5.3; 9.7]	1.2%
Mewhort H, 2014	2.3	[−5.6; 10.2]	1.1%
Perea−Gil I, 2018 Exp. 1	2.3	[−5.5; 10.1]	1.1%
Jiang Y, 2023	2.4	[−1.3; 6.1]	1.3%
Yang X, 2021 Exp. 1	2.8	[−6.9; 12.5]	1.1%
Hosoyama K, 2018	3.1	[−1.1; 7.3]	1.3%
Wassenaar J, 2016	3.2	[−3.3; 9.7]	1.2%
Mewhort H, 2016	3.8	[−2.2; 9.8]	1.2%
Wang Y, 2023	3.9	[2.1; 5.7]	1.3%
Lee J, 2018	4.4	[−32.8; 41.6]	0.3%
Feng J, 2024 Exp. 1	4.4	[−6.7; 15.5]	1.0%
Vasanthan V, 2022	4.6	[−6.5; 15.7]	1.0%
Spang MT, 2022 Exp. 2	4.6	[−4.0; 13.2]	1.1%
Tang J, 2017	4.9	[−5.7; 15.5]	1.0%
Gálvez−Montón C, 2015	5.2	[0.6; 9.8]	1.3%
Zhang Y, 2019 Exp. 2	5.2	[−10.7; 21.1]	0.8%
Baehr A, 2020 Exp. 3	5.3	[−3.0; 13.6]	1.1%
Chen W, 2016 Exp. 3	5.6	[1.5; 9.7]	1.3%
Blackburn N, 2015 Exp. 2	5.7	[0.2; 11.2]	1.2%
Chen C, 2013	5.8	[1.9; 9.7]	1.3%
Spang MT, 2022 Exp. 1	5.8	[−1.1; 12.7]	1.2%
Feng J, 2024 Exp. 4	6.7	[−6.2; 19.6]	0.9%
Neuta P, 2015 Exp. 1	6.8	[0.8; 12.8]	1.2%
Yanbo X, 2020	7.9	[−3.8; 19.6]	1.0%
Guan G, 2021	8.2	[6.3; 10.1]	1.3%
Wang N, 2019 Exp. 2	8.3	[6.2; 10.4]	1.3%
Shi J, 2019	8.4	[−24.2; 41.0]	0.3%
Vasanthan V, 2023	8.6	[−0.2; 17.4]	1.1%
Svystonyuk D, 2020 Exp. 1	8.9	[1.3; 16.5]	1.1%
Aubin H, 2023 Exp. 1	9.0	[0.6; 17.4]	1.1%
MacArthur J, 2013	10.0	[−0.0; 20.0]	1.0%
Perea−Gil I, 2018 Exp. 2	10.1	[4.0; 16.2]	1.2%
Rotem I, 2022	10.3	[1.7; 18.9]	1.1%
Francisco J, 2020	10.9	[−0.7; 22.5]	1.0%
Kashiyama N, 2022	11.0	[4.9; 17.1]	1.2%
Neuta P, 2015 Exp. 3	11.1	[5.1; 17.1]	1.2%
Duc Vu T, 2015	11.2	[6.8; 15.6]	1.3%
Pupkaite J, 2020 Exp. 2	11.2	[4.8; 17.6]	1.2%
Ahmadi A, 2014	11.4	[2.7; 20.1]	1.1%
Pupkaite J, 2020 Exp. 4	11.6	[3.8; 19.4]	1.1%
Baehr A, 2020 Exp. 1	12.0	[5.8; 18.2]	1.2%
Zhang L, 2024	12.4	[5.2; 19.6]	1.2%
McLaughlin S, 2019 Exp. 2	13.1	[5.1; 21.1]	1.1%
Bassat E, 2017	13.2	[5.9; 20.5]	1.2%
Chen W, 2016 Exp. 1	13.3	[7.9; 18.7]	1.2%
Toeg H, 2013	13.3	[3.2; 23.4]	1.0%
Gaffey A, 2015	13.5	[4.5; 22.5]	1.1%
Pupkaite J, 2020 Exp. 6	13.9	[7.2; 20.6]	1.2%
Yang X, 2021 Exp. 2	14.4	[3.9; 24.9]	1.0%
Blackburn N, 2015 Exp. 1	15.2	[10.1; 20.3]	1.3%
Hayam R, 2022 Exp. 2	15.2	[−0.3; 30.7]	0.8%
Wang Z, 2019 Exp. 1	16.0	[12.7; 19.3]	1.3%
McLaughlin S, 2019 Exp. 1	16.1	[9.9; 22.3]	1.2%
Baehr A, 2020 Exp. 2	16.9	[12.2; 21.6]	1.3%
Hao D, 2024	16.9	[12.7; 21.1]	1.3%
Lee J, 2024 Exp. 2	17.2	[−4.2; 38.6]	0.6%
Qiao L, 2019	17.6	[15.0; 20.2]	1.3%
Abdalla S, 2013	18.2	[10.9; 25.5]	1.2%
Huang k, 2020	18.7	[14.2; 23.2]	1.3%
Hume RD, 2023	18.7	[8.4; 29.0]	1.0%
Pupkaite J, 2020 Exp. 5	19.5	[14.7; 24.3]	1.3%
Aubin H, 2023 Exp. 2	19.7	[8.8; 30.6]	1.0%
Pupkaite J, 2020 Exp. 1	20.0	[14.4; 25.6]	1.2%
Zhang Y, 2019 Exp. 1	20.8	[7.6; 34.0]	0.9%
Feng J, 2024 Exp. 2	20.8	[14.9; 26.7]	1.2%
Chen W, 2016 Exp. 2	22.0	[15.1; 28.9]	1.2%
Shen D, 2011	22.2	[19.5; 24.9]	1.3%
Feng J, 2024 Exp. 3	22.3	[12.9; 31.7]	1.1%
Henry J, 2019	23.3	[16.6; 30.0]	1.2%
Blackburn N, 2015 Exp. 4	24.0	[15.5; 32.5]	1.1%
Yoon S, 2009	24.5	[17.1; 31.9]	1.2%
Sarig U, 2016 Exp. 2	24.8	[15.0; 34.6]	1.0%
Serpooshan V, 2013	25.8	[7.4; 44.2]	0.7%
Hayam R, 2022 Exp. 1	25.9	[11.1; 40.7]	0.8%
Lee J, 2024 Exp. 1	45.3	[36.5; 54.1]	1.1%
Seif−Naraghi S, 2013	46.8	[22.0; 71.6]	0.5%
Sarig U, 2016 Exp. 1	47.9	[45.1; 50.7]	1.3%
Random effects model	10.9	[8.7; 13.0]	100.0%

Het: $I^2 = 95\%$, $\tau^2 = 86.26$, $p = 8.057e-24$

Forest plot showing the effect of ECM treatment on Left Ventricular Ejection Fraction (LVEF). The effect of ECM treatment, versus control, on LVEF, expressed as mean difference (MD). MDs per individual experiment (row) are presented with a 95% confidence interval (CI). Left favouring the control and right favouring the ECM treatment. Grey boxes represent the study's weight in the pooled effect estimate. The pooled MD using a random effects model is represented by a red dotted line and a diamond, with the width of the diamond representing the 95% CI.

Supplementary Figure 3.

Study	MD	95% CI	Weight
Wang N, 2019 Exp. 1	−2.6	[−6.3; 1.1]	2.2%
Simpson D, 2007	−1.0	[−3.9; 1.9]	2.3%
Hao Y, 2022	−0.7	[−4.8; 3.4]	2.2%
Zhang Y, 2019 Exp. 2	0.3	[−8.2; 8.8]	1.9%
Wang Q, 2016	0.5	[−4.9; 5.9]	2.1%
Okada M, 2010 Exp. 2	0.6	[−5.0; 6.2]	2.1%
Yu J, 2009 Exp. 1	0.7	[−9.3; 10.7]	1.8%
Ravi S, 2012	1.0	[−1.9; 3.9]	2.3%
Wang Z, 2019 Exp. 2	1.1	[−1.9; 4.1]	2.3%
Yang X, 2021 Exp. 1	1.4	[−3.9; 6.7]	2.1%
Wang Y, 2023	1.8	[0.2; 3.4]	2.3%
Nair RS, 2023	1.9	[−3.2; 7.0]	2.1%
Gao L, 2017	2.0	[−3.2; 7.2]	2.1%
Hosoyama K, 2018	2.6	[−1.1; 6.3]	2.2%
Huang k, 2020	2.9	[0.4; 5.4]	2.3%
Tang J, 2017	3.9	[−0.4; 8.2]	2.2%
Jiang Y, 2023	3.9	[1.3; 6.5]	2.3%
Guan G, 2021	4.2	[3.4; 5.0]	2.3%
Kofidis T, 2005 Exp.1	4.3	[−3.5; 12.1]	1.9%
Spang MT, 2022 Exp. 2	4.4	[−0.8; 9.6]	2.1%
Kofidis T, 2005 Exp. 2	4.5	[1.4; 7.6]	2.3%
Rotem I, 2022	5.3	[0.6; 10.0]	2.2%
Wang N, 2019 Exp. 2	5.4	[1.1; 9.7]	2.2%
Bassat E, 2017	5.8	[1.6; 10.0]	2.2%
Duc Vu T, 2015	6.0	[4.0; 8.0]	2.3%
Shi J, 2019	6.2	[−13.0; 25.4]	1.1%
Okada M, 2010 Exp. 1	7.4	[3.6; 11.2]	2.2%
Wang Z, 2019 Exp. 1	7.7	[5.1; 10.3]	2.3%
Qiao L, 2019	7.8	[6.8; 8.8]	2.3%
Yang X, 2021 Exp. 2	7.9	[2.2; 13.6]	2.1%
Ahmadi A, 2014	9.1	[3.5; 14.7]	2.1%
D'Amore A, 2016	9.2	[3.2; 15.2]	2.1%
Aubin H, 2023 Exp. 1	9.3	[2.0; 16.6]	2.0%
Zhang Y, 2019 Exp. 1	9.6	[−6.2; 25.4]	1.3%
Hayam R, 2022 Exp. 2	9.6	[1.8; 17.4]	1.9%
Hao D, 2024	10.9	[8.5; 13.3]	2.3%
Yu J, 2009 Exp. 2	11.1	[−5.2; 27.4]	1.3%
Abdalla S, 2013	12.3	[7.3; 17.3]	2.2%
Lee J, 2024 Exp. 2	12.8	[0.7; 24.9]	1.6%
Hume RD, 2023	13.6	[5.8; 21.4]	1.9%
Chen C, 2008	14.0	[0.5; 27.5]	1.5%
Hayam R, 2022 Exp. 1	14.7	[7.2; 22.2]	2.0%
Shen D, 2011	15.9	[13.8; 18.0]	2.3%
Frederick J, 2010	16.0	[11.5; 20.5]	2.2%
Aubin H, 2023 Exp. 2	17.5	[8.3; 26.7]	1.8%
Sarig U, 2016 Exp. 2	27.6	[21.3; 33.9]	2.1%
Serpooshan V, 2013	33.3	[11.5; 55.1]	0.9%
Lee J, 2024 Exp. 1	37.9	[30.5; 45.3]	2.0%
Sarig U, 2016 Exp. 1	45.5	[41.2; 49.8]	2.2%
Random effects model	8.2	[5.6; 10.9]	100.0%

Het: $I^2 = 94\%$, $\tau^2 = 80.34$, $p = 1.751e{-}09$

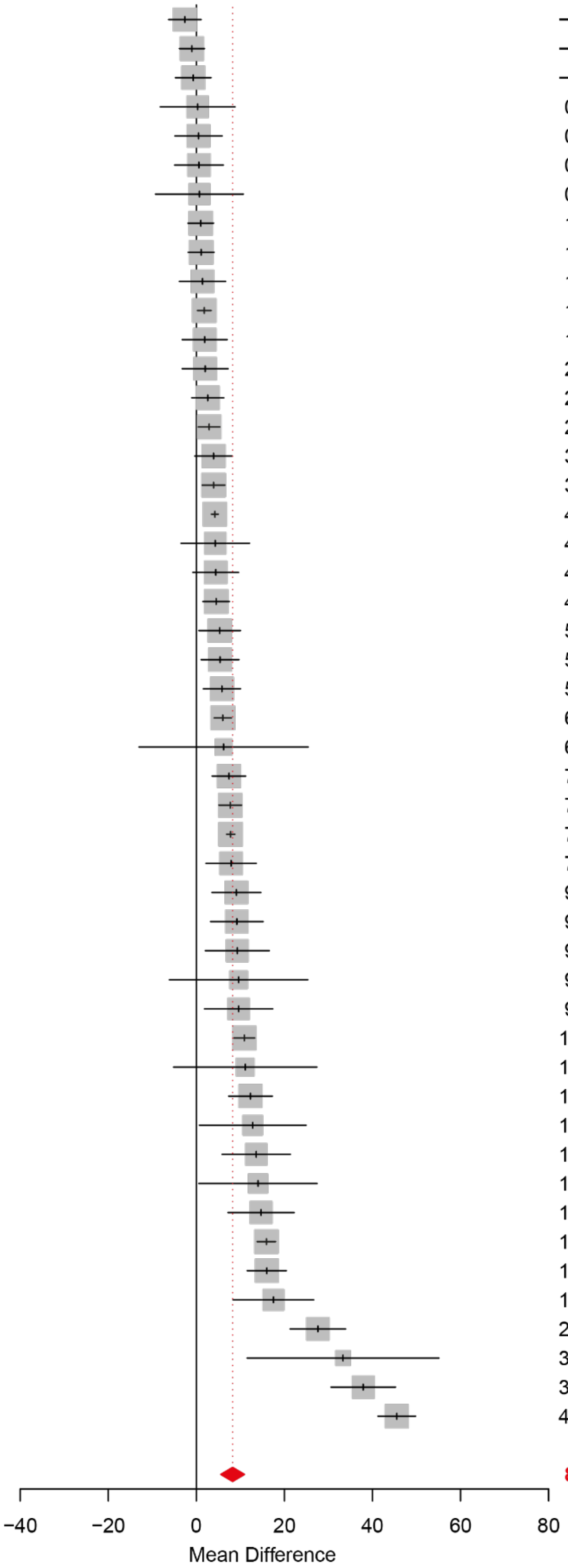

Forest plot showing the effect of ECM treatment on Fractional Shortening. The effect of ECM treatment, versus control, on fractional shortening, expressed as mean difference (MD). MDs per individual experiment (row) are presented with a 95% confidence interval

(CI). Left favouring the control and right favouring the ECM treatment. Grey boxes represent the study’s weight in the pooled effect estimate. The pooled MD using a random effects model is represented by a red dotted line and a diamond, with the width of the diamond representing the 95% CI.

Supplementary Figure 4.

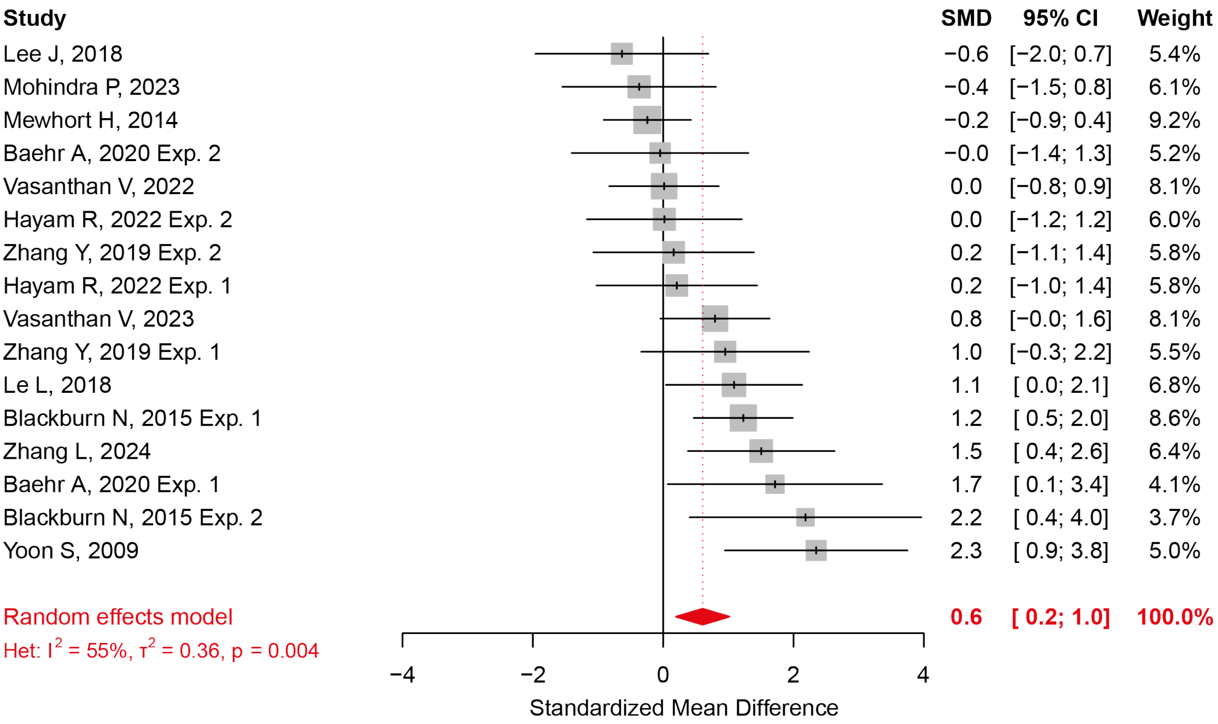

Forest plot showing the effect of ECM treatment on Stroke Volume. The effect of ECM treatment, versus control, on stroke volume, expressed as standardized mean difference (SMD). SMDs per individual experiment (row) are presented with a 95% confidence interval (CI). Left favouring the control and right favouring the ECM treatment. Grey boxes represent the study's weight in the pooled effect estimate. The pooled SMD using a random effects model is represented by a red dotted line and a diamond, with the width of the diamond representing the 95% CI.

Supplementary Figure 5.

Study	MD	95% CI	Weight
Wang N, 2019 Exp. 2	-78.7	[-80.8; -76.6]	1.5%
Yoon S, 2014 Exp. 4	-48.3	[-57.7; -38.9]	1.3%
Wang Z, 2019 Exp. 1	-35.4	[-41.6; -29.2]	1.4%
Wang N, 2019 Exp. 1	-29.2	[-34.2; -24.2]	1.5%
Chen W, 2016 Exp. 2	-28.5	[-31.0; -26.0]	1.5%
Toeg H, 2013	-27.5	[-34.5; -20.5]	1.4%
Yoon S, 2014 Exp. 5	-26.7	[-53.4; 0.0]	0.7%
Yoon S, 2014 Exp. 1	-25.6	[-32.8; -18.4]	1.4%
Abdalla S, 2013	-23.4	[-32.5; -14.3]	1.4%
D'Amore A, 2016	-22.3	[-29.6; -15.0]	1.4%
McLaughlin S, 2019 exp. 1	-19.7	[-43.8; 4.4]	0.8%
Chen W, 2016 Exp. 1	-19.1	[-23.4; -14.8]	1.5%
Blackburn N, 2015	-18.8	[-27.2; -10.4]	1.4%
Feng J, 2024 Exp. 3	-18.5	[-26.2; -10.8]	1.4%
Lee J, 2024 Exp. 1	-18.0	[-28.5; -7.5]	1.3%
Francisco J, 2020	-17.9	[-25.4; -10.4]	1.4%
McLaughlin S, 2019 exp. 2	-17.5	[-40.1; 5.1]	0.8%
Wang Z, 2019 Exp. 2	-17.2	[-25.8; -8.6]	1.4%
Pupkaite J, 2020 Exp. 5	-17.0	[-30.3; -3.7]	1.2%
Lebedeva A, 2020	-16.9	[-48.5; 14.7]	0.6%
Okada M, 2010 Exp. 1	-16.9	[-32.1; -1.7]	1.1%
Pupkaite J, 2020 Exp. 1	-16.7	[-30.3; -3.1]	1.2%
Guan G, 2021	-15.8	[-19.1; -12.5]	1.5%
Qiao L, 2019	-14.7	[-15.7; -13.7]	1.5%
Yoon S, 2009	-14.6	[-20.7; -8.5]	1.4%
Hosoyama K, 2018	-14.5	[-41.4; 12.4]	0.7%
Kashiyama N, 2022	-14.5	[-20.2; -8.8]	1.5%
Yoon S, 2014 Exp. 2	-13.7	[-41.2; 13.8]	0.7%
Chen C, 2013	-13.2	[-18.3; -8.1]	1.5%
Feng J, 2024 Exp. 2	-13.1	[-24.3; -1.9]	1.3%
Baehr A, 2020 Exp. 2	-12.3	[-18.0; -6.6]	1.5%
Pupkaite J, 2020 Ex.p 4	-12.2	[-25.7; 1.3]	1.2%
Hume RD, 2023	-12.0	[-18.3; -5.7]	1.4%
Pupkaite J, 2020 Exp. 2	-11.9	[-26.7; 2.9]	1.1%
Jiang Y, 2023	-11.6	[-23.8; 0.6]	1.2%
Henry J, 2019	-10.5	[-12.5; -8.5]	1.5%
Baehr A, 2020 Exp. 1	-10.4	[-16.1; -4.7]	1.5%
Shen D, 2011	-10.0	[-20.4; 0.4]	1.3%
Hao D, 2024	-9.1	[-9.8; -8.4]	1.5%
MacArthur J, 2013	-8.9	[-13.2; -4.6]	1.5%
Bassat E, 2017	-8.7	[-14.3; -3.1]	1.5%
Shi J, 2019	-8.5	[-12.4; -4.6]	1.5%
Ahmadi A, 2014	-7.4	[-13.5; -1.3]	1.4%
Pupkaite J, 2020 Exp. 3	-7.3	[-19.7; 5.1]	1.2%
Feng J, 2024 Exp. 4	-7.3	[-16.7; 2.1]	1.3%
Chen W, 2016 Exp. 3	-6.4	[-10.7; -2.1]	1.5%
Zhang Y, 2019 Exp. 1	-5.1	[-16.8; 6.6]	1.3%
Chang M, 2016	-4.9	[-8.5; -1.3]	1.5%
Feng J, 2024 Exp. 1	-4.8	[-18.6; 9.0]	1.2%
Zhang L, 2024	-4.1	[-6.6; -1.6]	1.5%
Frederick J, 2010	-3.8	[-5.6; -2.0]	1.5%
Lee J, 2024 Exp. 2	-3.8	[-22.1; 14.5]	1.0%
Chen C, 2014	-3.6	[-6.8; -0.4]	1.5%
Yu J, 2009 Exp. 1	-3.6	[-6.6; -0.6]	1.5%
Shepherd B, 2007	-3.5	[-27.4; 20.4]	0.8%
Huang k, 2020	-3.2	[-9.3; 2.9]	1.4%
Wang Y, 2023	-3.2	[-4.9; -1.5]	1.5%
Gaffey A, 2015	-3.0	[-8.7; 2.7]	1.5%
Tang J, 2017	-3.0	[-8.1; 2.1]	1.5%
Wang Q, 2016	-2.9	[-18.8; 13.0]	1.1%
Duc Vu T, 2015	-2.7	[-4.0; -1.4]	1.5%
Pupkaite J, 2020 Exp. 6	-2.6	[-16.8; 11.6]	1.1%
Galvez-Monton C, 2015	-2.3	[-5.8; 1.2]	1.5%
Gao L, 2017	-1.9	[-16.9; 13.1]	1.1%
Perea-Gil I, 2018 Exp. 2	-1.8	[-5.4; 1.8]	1.5%
Yu J, 2009 Exp. 2	-1.6	[-7.8; 4.6]	1.4%
Gaetani R, 2015	-1.5	[-4.7; 1.7]	1.5%
Zhang Y, 2019 Exp. 2	-0.9	[-12.4; 10.6]	1.3%
Nair RS, 2023	-0.6	[-6.2; 5.0]	1.5%
Hao Y, 2022	-0.4	[-5.1; 4.3]	1.5%
Perea-Gil I, 2018 Exp. 1	-0.1	[-6.1; 5.9]	1.5%
Spang MT, 2022 Exp. 2	0.3	[-2.4; 3.0]	1.5%
Ravi S, 2012	0.6	[-6.4; 7.6]	1.4%
Okada M, 2010 Exp. 2	1.7	[-16.0; 19.4]	1.0%
Feng J, 2024 Exp. 5	5.0	[-3.5; 13.5]	1.4%
Yoon S, 2014 Exp. 3	7.9	[-21.1; 36.9]	0.6%
Random effects model	-11.7	[-14.7; -8.6]	100.0%

Het: $I^2 = 99\%$, $\tau^2 = 153.87$, $p = 3.699e-14$

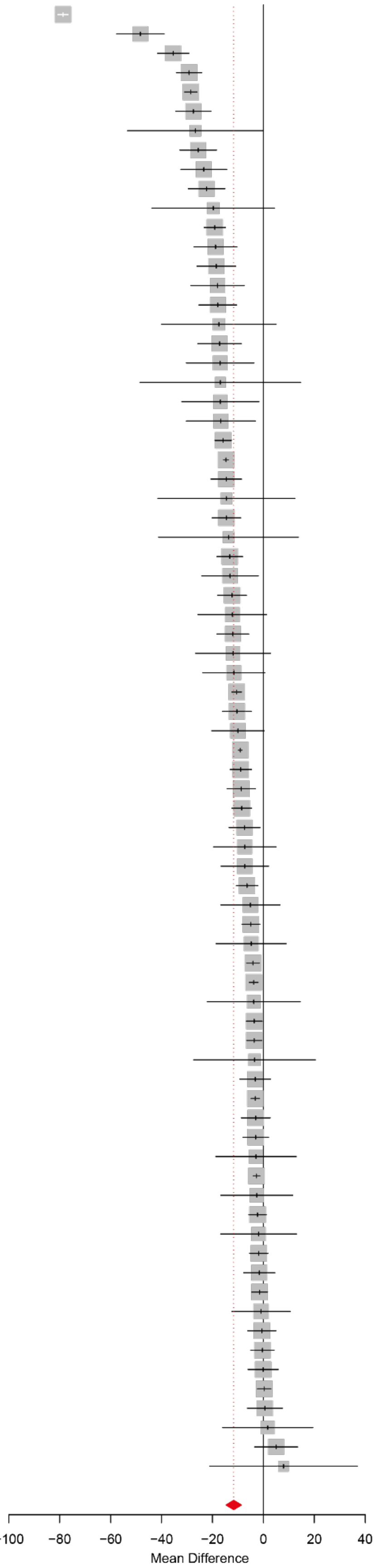

Forest plot showing the effect of ECM treatment on Infarct Size. The effect of ECM treatment, versus control, on infarct size, expressed as mean difference (MD). MDs per individual experiment (row) are presented with a 95% confidence interval (CI). Left favouring the control and right favouring the ECM treatment. Grey boxes represent the study's weight in the pooled effect estimate. The pooled MD using a random effects model is represented by a red dotted line and a diamond, with the width of the diamond representing the 95% CI.

Supplementary Figure 6.

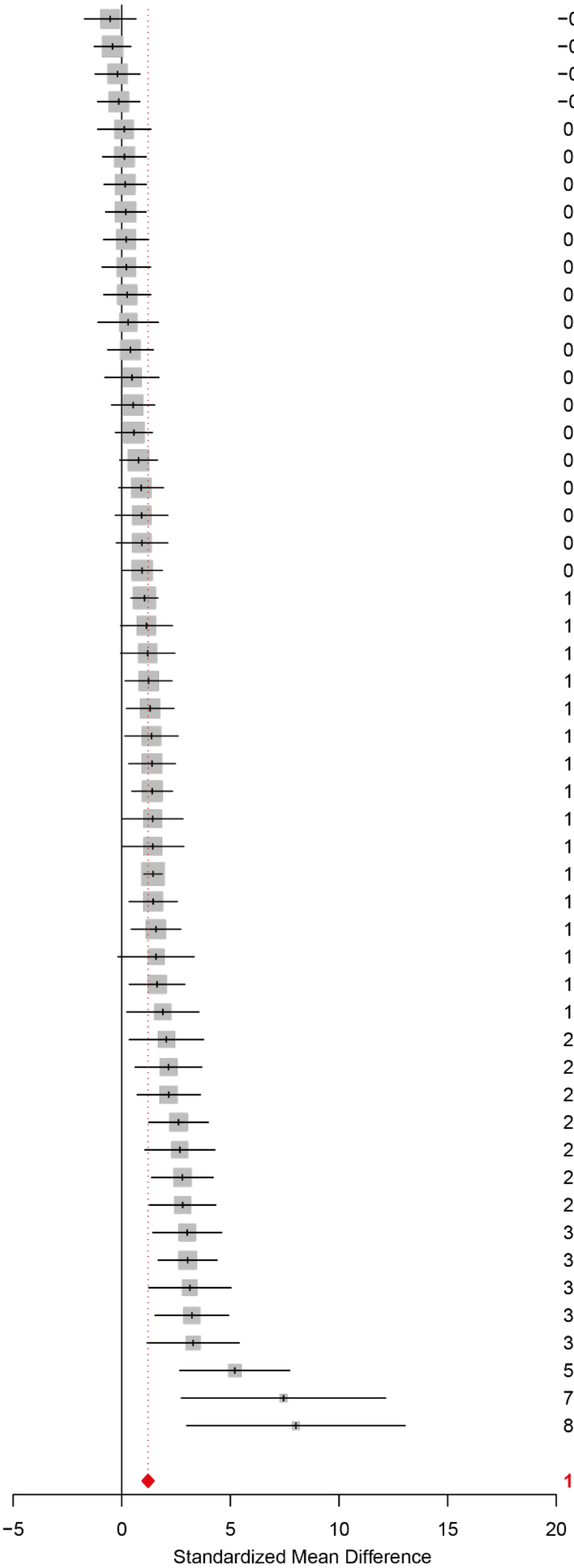

Study	SMD	95% CI	Weight
Mohindra P, 2023	−0.5	[−1.7; 0.7]	2.0%
Cortes−Morichetti M, 2007	−0.4	[−1.3; 0.4]	2.5%
Xiang Z, 2006 Exp. 2	−0.2	[−1.2; 0.8]	2.2%
Yu J, 2009 Exp. 8	−0.1	[−1.1; 0.8]	2.3%
Zhang Y, 2019 Exp. 2	0.1	[−1.1; 1.3]	2.0%
Xiang Z, 2006 Exp. 1	0.1	[−0.9; 1.1]	2.3%
Gao L, 2017	0.2	[−0.8; 1.1]	2.3%
Wang Q, 2016	0.2	[−0.8; 1.1]	2.4%
Tang J, 2017	0.2	[−0.8; 1.2]	2.2%
Kofidis T, 2005a Exp. 1	0.2	[−0.9; 1.3]	2.1%
Bassat E, 2017 Exp. 1	0.3	[−0.8; 1.3]	2.2%
Yoon S, 2014 Exp. 3	0.3	[−1.1; 1.7]	1.8%
Chang M, 2016	0.4	[−0.7; 1.5]	2.2%
Lee J, 2024 Exp. 2	0.5	[−0.8; 1.7]	2.0%
Yu J, 2009 Exp. 4	0.5	[−0.5; 1.5]	2.3%
Spang MT, 2022 Exp. 2	0.6	[−0.3; 1.4]	2.5%
Svystonyuk D, 2020 Exp. 2	0.8	[−0.1; 1.6]	2.5%
Yu J, 2009 Exp. 1	0.9	[−0.1; 1.9]	2.2%
McLaughlin S, 2019 Exp. 2	0.9	[−0.3; 2.1]	2.0%
Kofidis T, 2005a Exp. 2	0.9	[−0.3; 2.1]	2.0%
MacArthur J, 2013	0.9	[0.0; 1.9]	2.4%
Frederick J, 2010	1.0	[0.4; 1.7]	2.8%
Wang N, 2019 Exp. 1	1.1	[−0.1; 2.3]	2.0%
McLaughlin S, 2019 Exp. 1	1.2	[−0.1; 2.4]	2.0%
Yu J, 2009 Exp. 2	1.2	[0.2; 2.3]	2.2%
Yu J, 2009 Exp. 6	1.3	[0.2; 2.4]	2.2%
Bassat E, 2017 Exp. 2	1.4	[0.1; 2.6]	2.0%
Le L, 2018	1.4	[0.3; 2.5]	2.2%
Svystonyuk D, 2020 Exp. 1	1.4	[0.5; 2.3]	2.4%
Yoon S, 2014 Exp. 5	1.4	[0.0; 2.8]	1.8%
Lee J, 2024 Exp. 1	1.4	[0.0; 2.9]	1.8%
Yanbo X, 2020	1.4	[1.0; 1.9]	3.0%
Yu J, 2009 Exp. 3	1.4	[0.3; 2.6]	2.1%
Yu J, 2009 Exp. 7	1.6	[0.4; 2.7]	2.1%
Chen W, 2016 Exp. 3	1.6	[−0.2; 3.3]	1.4%
Mewhort H, 2016	1.6	[0.3; 2.9]	1.9%
Wang Y, 2023	1.9	[0.2; 3.6]	1.5%
Yoon S, 2014 Exp. 2	2.1	[0.3; 3.8]	1.5%
Zhang Y, 2019 Exp. 1	2.1	[0.6; 3.7]	1.6%
Kofidis T, 2005b Exp. 2	2.2	[0.7; 3.6]	1.7%
Yu J, 2009 Exp. 5	2.6	[1.2; 4.0]	1.8%
Shi J, 2019	2.7	[1.1; 4.3]	1.5%
Ahmadi A, 2014	2.8	[1.4; 4.2]	1.8%
D'Amore A, 2016	2.8	[1.3; 4.3]	1.6%
Yoon S, 2009	3.0	[1.4; 4.6]	1.6%
Gaetani R, 2015	3.0	[1.7; 4.4]	1.8%
Yoon S, 2014 Exp. 4	3.1	[1.2; 5.0]	1.3%
Wang N, 2019 Exp. 2	3.2	[1.5; 4.9]	1.5%
Yoon S, 2014 Exp. 1	3.3	[1.2; 5.4]	1.1%
Kofidis T, 2005b Exp. 1	5.2	[2.7; 7.7]	0.9%
Chen W, 2016 Exp. 1	7.4	[2.7; 12.2]	0.3%
Chen W, 2016 Exp. 2	8.0	[3.0; 13.0]	0.3%
**Random effects model**	**1.2**	**[0.9; 1.5]**	**100.0%**

Het: I^2 = 64%, τ^2 = 0.62, p = 1.321e-17

Forest plot showing the effect of ECM treatment on Wall Thickening. The effect of ECM treatment, versus control, on wall thickening, expressed as standardized mean difference

(SMD). SMDs per individual experiment (row) are presented with a 95% confidence interval (CI). Left favouring the control and right favouring the ECM treatment. Grey boxes represent the study's weight in the pooled effect estimate. The pooled MD using a random effects model is represented by a red dotted line and a diamond, with the width of the diamond representing the 95% CI.

Supplementary Files

Supplementary File 1 – full search strings for Pubmed 28-06-2024

Updated search 28-06-2024

PubMed	28-06-2024	2,448 hits without reviews
#1	Heart injury<br><br>Hits: 1,883,525	((“myocardial ischemia”[Mesh] OR “coronary occlusion”[Mesh] OR “heart transplantation”[Mesh] OR “heart failure”[Mesh] OR “heart”[Mesh] OR “myocardium”[Mesh] OR “cardiac fibrosis”[tiab] OR heart[tiab] OR hearts[tiab] OR cardiac[tiab] OR myocardial[tiab] OR myocardium[tiab] OR myocardia[tiab] OR myocard[tiab]) OR ((“ischemia”[Mesh:noexp] OR “infarction”[Mesh:noexp] OR “warm ischemia”[Mesh] OR “cold ischemia”[Mesh] OR “reperfusion injury”[Mesh] OR “transplantation”[Mesh:noexp] OR “graft survival”[Mesh] OR “graft rejection”[Mesh] OR “transplants”[Mesh] OR ischemia[tiab] OR ischaemia[tiab] OR ischemic[tiab] OR ischaemic[tiab] OR infarct[tiab] OR infarcts[tiab] OR infarction[tiab] OR infarctions[tiab] OR infarcted[tiab] OR “reperfusion injury”[tiab] OR “reperfusion injuries”[tiab] OR I/R[tiab] OR IRI[tiab] OR occlusion[tiab] OR failure[tiab] OR decompensation[tiab] OR insufficiency[tiab] OR incompetence[tiab] OR overload[tiab] OR transplantation[tiab] OR transplantations[tiab] OR transplant[tiab] OR transplants[tiab] OR graft[tiab] OR grafts[tiab]) AND (heart[tiab] OR hearts[tiab] OR cardiac[tiab] OR myocardial[tiab] OR myocardium[tiab] OR myocardia[tiab] OR myocard[tiab]))) NOT ("Heart Valves"[MeSH:noexp] OR "Aortic Valve"[MeSH] OR "Chordae Tendineae"[MeSH] OR "Mitral Valve"[MeSH] OR "Pulmonary Valve"[MeSH] OR "Tricuspid Valve"[MeSH] OR "Heart Valves"[tiab] OR "Heart Valve"[tiab] OR "Aortic Valves"[tiab] OR "Aortic Valve"[tiab] OR "Chordae Tendineae"[tiab] OR "Mitral Valves"[tiab] OR "Mitral Valve"[tiab] OR "Pulmonary Valves"[tiab] OR "Pulmonary Valve"[tiab] OR "Tricuspid Valves"[tiab] OR "Tricuspid Valve"[tiab] OR "Cardiac Valves"[tiab] OR "Cardiac Valve"[tiab])
#2	Repair<br><br>Hits: 830,210	"Regeneration"[MeSH:noexp] OR "Wound Healing"[Mesh:noexp] OR "Regenerative Medicine"[Mesh] OR “Tissue Scaffolds”[MeSH] OR “Tissue Engineering”[MeSH] OR regeneration[tiab] OR regenerations[tiab] OR regenerated[tiab] OR regenerative[tiab] OR “wound healing”[tiab] OR “wound healings”[tiab] OR renewal[tiab] OR repair[tiab] OR “Tissue Engineering”[tiab] OR “Tissue Scaffolds”[tiab] OR “Tissue Scaffold”[tiab]
#2	ECM<br><br>Hits: 701,802	"Extracellular Matrix"[Mesh:noexp] OR "Decellularized Extracellular Matrix"[Mesh] OR "Extracellular Matrix Proteins"[Mesh] OR “Glycosaminoglycans”[Mesh] OR “Chondroitin”[Mesh] OR “Chondroitin Sulfates”[Mesh] OR “Dermatan Sulfate”[Mesh] OR “Heparin”[Mesh] OR “Heparin, Low-Molecular-Weight ”[Mesh] OR “Heparinoids”[Mesh] OR “Heparitin Sulfate”[Mesh] OR “Heparan Sulfate Proteoglycans”[Mesh] OR “Hyaluronic Acid”[Mesh] OR “Keratan Sulfate”[Mesh] OR “Dalteparin”[Mesh] OR “Enoxaparin”[Mesh] OR “Nadroparin”[Mesh] OR “Tinzaparin”[Mesh] OR “Biocompatible Materials”[Mesh] OR “Extracellular Matrix Protein”[tiab] OR “Extracellular Matrix Proteins”[tiab] OR “Extracellular Matrix”[tiab] OR “Extracellular Matrices”[tiab] OR “ECM”[tiab] OR “ECMs”[tiab] OR “Decellularized Extracellular Matrix”[tiab] OR “Decellularized Extracellular Matrices”[tiab] OR “Decellularized ECM”[tiab] OR “Decellularized ECMs”[tiab] OR “Glycosaminoglycans”[tiab] OR “Glycosaminoglycan”[tiab] OR “Chondroitin”[tiab] OR “Chondroitin Sulfates”[tiab] OR “Chondroitin Sulfate”[tiab] OR “Dermatan Sulfates”[tiab] OR “Dermatan Sulfate”[tiab] OR “Heparin”[tiab] OR “Dalteparin”[tiab] OR “Enoxaparin”[tiab] OR “Nadroparin”[tiab] OR “Tinzaparin”[tiab] OR “Heparinoids”[tiab] OR “Heparitin Sulfate”[tiab] OR “Heparan Sulfate Proteoglycans”[tiab] OR “Hyaluronic Acid”[tiab] OR “Keratan Sulfate”[tiab] OR “Biocompatible Materials”[tiab] OR Proteoglycans[tiab] OR Proteoglycan[tiab] OR “Activated-Leukocyte Cell Adhesion Molecule”[tiab] OR “ADAMTS Proteins”[tiab] OR “ADAMTS1 Protein”[tiab] OR “ADAMTS13 Protein”[tiab] OR “ADAMTS4 Protein”[tiab] OR “ADAMTS5 Protein”[tiab] OR “ADAMTS7 Protein”[tiab] OR “ADAMTS9 Protein”[tiab] OR “Aggrecan”[tiab] OR “Aggrecans”[tiab] OR “Cartilage Oligomeric Matrix Protein”[tiab] OR “Cartilage Oligomeric Matrix Proteins”[tiab] OR “CCN Intercellular Signaling Protein”[tiab] OR “CCN Intercellular Signaling Proteins”[tiab] OR “Connective Tissue Growth Factor”[tiab] OR “Connective Tissue Growth

		Factors”[tiab] OR “Cysteine-Rich Protein 61”[tiab] OR “Nephroblastoma Overexpressed Protein”[tiab] OR “Collagen”[tiab] OR “Collagens”[tiab] OR “Fibrillar Collagens”[tiab] OR “Non-Fibrillar Collagens”[tiab] OR “Procollagen”[tiab] OR “Tropocollagen”[tiab] OR “Elastin”[tiab] OR “Tropoelastin”[tiab] OR “Fibrillins”[tiab] OR “Fibrillin-1”[tiab] OR “Fibrillin-2”[tiab] OR “Fibronectins”[tiab] OR “Integrin-Binding Sialoprotein”[tiab] OR “Laminin”[tiab] OR “Latent TGF-beta Binding Proteins”[tiab] OR “Matrilin Proteins”[tiab] OR “Netrins”[tiab] OR “Netrin-1”[tiab] OR “Osteopontin”[tiab] OR “Reelin Protein”[tiab] OR “Small Leucine-Rich Proteoglycans”[tiab] OR “Biglycan”[tiab] OR “Decorin”[tiab] OR “Fibromodulin”[tiab] OR “Lumican”[tiab] OR “Tenascin”[tiab] OR “Versicans”[tiab] OR “Vitronectin”[tiab] OR “Chondroitin Sulfate Proteoglycans”[tiab] OR “Aggrecans”[tiab] OR “Versicans”[tiab] OR “Versican”[tiab] OR “Small Leucine-Rich Proteoglycans”[tiab] OR “Biglycan”[tiab] OR “Decorin”[tiab] OR “Fibromodulin”[tiab] OR “Lumican”[tiab] OR “Hyaluronan Receptors”[tiab] OR “Hyaluronan Receptor”[tiab] OR “Glypicans”[tiab] OR “Syndecans”[tiab] OR “Syndecan”[tiab] OR “Syndecan-1”[tiab] OR “Syndecan-2”[tiab] OR “Syndecan-3”[tiab] OR “Syndecan-4”[tiab] OR “Syndecan1”[tiab] OR “Syndecan2”[tiab] OR “Syndecan3”[tiab] OR “Syndecan4”[tiab] OR “Glypican”[tiab] OR “Glypican-5”[tiab] OR “Glypican5”[tiab] OR “Glypican-3”[tiab] OR “Glypican3”[tiab] OR “Glypican-4”[tiab] OR “Glypican4”[tiab] OR “Glypican-1”[tiab] OR “Glypican1”[tiab] OR “Glypican-2”[tiab] OR “Glypican2”[tiab] OR “Biglycan”[tiab] OR “Decorin”[tiab] OR “Fibromodulin”[tiab] OR “Lumican”[tiab] OR “Fibrillar Collagen”[tiab] OR “Collagen Type I”[tiab] OR “Collagen Type II”[tiab] OR “Collagen Type III”[tiab] OR “Collagen Type V”[tiab] OR “Collagen Type XI”[tiab] OR “Non-Fibrillar Collagen”[tiab] OR “Collagen Type IV”[tiab] OR “Collagen Type VI”[tiab] OR “Collagen Type VII”[tiab] OR “Collagen Type VIII”[tiab] OR “Collagen Type X”[tiab] OR “Collagen Type XIII”[tiab] OR “Collagen Type XVIII +”[tiab] OR “Fibril-Associated Collagens”[tiab] OR “Collagen Type IX”[tiab] OR “Collagen Type XII”[tiab] OR “Endostatins”[tiab] OR “Endostatin”[tiab] OR “Fibrillin”[tiab] OR “Fibronectins”[tiab] OR “Fibronectin”[tiab]
#4	Animal models<br><br>Hits: 8,119,974	(animal experimentation[MeSH] OR models, animal[MeSH] OR Animals[Mesh:noexp] OR animal population groups [MeSH] OR chordata[MeSH Terms:noexp] OR vertebrates[MeSH Terms:noexp] OR amphibians[MeSH] OR birds[MeSH] OR fishes[MeSH] OR reptiles[MeSH] OR mammals[MeSH Terms:noexp] OR primates[MeSH Terms:noexp] OR eutheria[MeSH Terms:noexp] OR artiodactyla[MeSH] OR carnivore[MeSH] OR cephalopoda[MeSH] OR cetacea[MeSH] OR chiroptera[MeSH] OR elephants[MeSH] OR hyraxes[MeSH] OR insectivora[MeSH] OR lagomorpha[MeSH] OR marsupialia[MeSH] OR monotremata[MeSH] OR perissodactyla[MeSH] OR Proboscidea Mammal[MeSH Terms:noexp] OR rodentia[MeSH] OR scandentia[MeSH] OR sirenia[MeSH] OR cingulata[MeSH] OR haplorhini[MeSH Terms:noexp] OR strepsirhini[MeSH] OR platyrrhini[MeSH] OR tarsii[MeSH] OR catarrhini[MeSH Terms:noexp] OR cercopithecidae[MeSH] OR hylobatidae[MeSH] OR hominidae[MeSH Terms:noexp] OR gorilla gorilla[MeSH] OR pan paniscus[MeSH] OR pan troglodytes[MeSH] OR pongo[MeSH]) OR ((rat[tiab] OR rats[tiab] OR animal[tiab] OR animals[tiab] OR mice[tiab] OR in vivo[tiab] OR mouse[tiab] OR rabbit[tiab] OR rabbits[tiab] OR murine[tiab] OR pig[tiab] OR pigs[tiab] OR dog[tiab] OR dogs[tiab] OR bovine[tiab] OR fish[tiab] OR vertebrate[tiab] OR vertebrates[tiab] OR cat[tiab] OR cats[tiab] OR rodent[tiab] OR rodents[tiab] OR mammal[tiab] OR mammals[tiab] OR chicken[tiab] OR chickens[tiab] OR monkey[tiab] OR monkeys[tiab] OR sheep[tiab] OR canine[tiab] OR canines[tiab] OR porcine[tiab] OR cattle[tiab] OR bird[tiab] OR birds[tiab] OR hamster[tiab] OR hamsters[tiab] OR primate[tiab] OR primates[tiab] OR cow[tiab] OR cows[tiab] OR chick[tiab] OR horse[tiab] OR horses[tiab] OR avian[tiab] OR avians[tiab] OR calf[tiab] OR swine[tiab] OR swines[tiab] OR xenopus[tiab] OR turkeys[tiab] OR bear[tiab] OR bears[tiab] OR frog[tiab] OR frogs[tiab] OR zebrafish[tiab] OR goat[tiab] OR goats[tiab] OR equine[tiab] OR calves[tiab] OR poultry[tiab] OR macaque[tiab] OR macaques[tiab] OR mole[tiab] OR moles[tiab] OR ovine[tiab] OR lamb[tiab] OR lambs[tiab] OR fishes[tiab] OR diptera[tiab] OR amphibian[tiab] OR amphibians[tiab] OR snake[tiab] OR snakes[tiab] OR ruminant[tiab] OR ruminants[tiab] OR hen[tiab] OR hens[tiab] OR piglet[tiab] OR piglets[tiab] OR feline[tiab] OR felines[tiab] OR simian[tiab] OR simians[tiab] OR laevis[tiab] OR trout[tiab] OR trouts[tiab] OR teleost[tiab] OR teleosts[tiab] OR salmon[tiab] OR salmons[tiab] OR seal[tiab] OR seals[tiab] OR bull[tiab] OR bulls[tiab] OR ewe[tiab] OR ewes[tiab] OR hedgehog[tiab] OR hedgehogs[tiab] OR macaca[tiab] OR macacas[tiab] OR proteus[tiab] OR pigeon[tiab] OR pigeons[tiab] OR bat[tiab] OR bats[tiab] OR duck[tiab] OR ducks[tiab] OR chimpanzee[tiab] OR chimpanzees[tiab] OR baboon[tiab] OR baboons[tiab] OR deer[tiab] OR rana[tiab] OR ranas[tiab] OR carp[tiab] OR carps[tiab] OR heifer[tiab] OR swallow[tiab] OR swallows[tiab] OR lizard[tiab] OR lizards[tiab] OR canis[tiab] OR sow[tiab] OR sows[tiab] OR cynomolgus[tiab] OR quail[tiab] OR quails[tiab] OR reptile[tiab] OR reptiles[tiab] OR turtle[tiab] OR turtles[tiab] OR buffalo[tiab] OR gerbil[tiab] OR gerbils[tiab] OR boar[tiab] OR boars[tiab] OR squirrel[tiab] OR squirrels[tiab] OR oncorhynchus[tiab] OR mus[tiab] OR toad[tiab] OR toads[tiab] OR fowl[tiab] OR fowls[tiab] OR rerio[tiab] OR danio[tiab] OR ara[tiab] OR aras[tiab] OR musculus[tiab] OR tadpole[tiab] OR tadpoles[tiab] OR mulatta[tiab] OR salmo[tiab] OR ram[tiab] OR eagle[tiab] OR eagles[tiab] OR ferret[tiab] OR ferrets[tiab] OR goldfish[tiab] OR catfish[tiab] OR whale[tiab] OR whales[tiab] OR fox[tiab] OR foxes[tiab] OR ape[tiab] OR apes[tiab] OR elephant[tiab] OR elephants[tiab] OR bos[tiab] OR marmoset[tiab] OR marmosets[tiab] OR cod[tiab] OR cods[tiab] OR shark[tiab] OR sharks[tiab] OR wolf[tiab] OR eel[tiab] OR eels[tiab] OR auratus[tiab] OR rattus[tiab] OR zebra[tiab] OR zebras[tiab] OR tilapia[tiab] OR tilapias[tiab] OR gilt[tiab] OR camel[tiab] OR camels[tiab] OR squid[tiab] OR gallus[tiab] OR marsupial[tiab] OR marsupials[tiab] OR vole[tiab] OR voles[tiab] OR fascicularis[tiab] OR ovis[tiab] OR salmonid[tiab] OR salmonids[tiab] OR tiger[tiab] OR tigers[tiab] OR dolphin[tiab] OR dolphins[tiab] OR robin[tiab] OR robins[tiab] OR carpio[tiab] OR opossum[tiab] OR opossums[tiab] OR cyprinus[tiab] OR salamander[tiab] OR salamanders[tiab] OR felis[tiab] OR mink[tiab] OR minks[tiab] OR swan[tiab] OR swans[tiab] OR norvegicus[tiab] OR bufo[tiab] OR torpedo[tiab] OR bass[tiab] OR lamprey[tiab] OR lampreys[tiab] OR sus[tiab] OR python[tiab] OR pythons[tiab] OR tetrapod[tiab] OR tetrapods[tiab] OR shrew[tiab] OR shrews[tiab] OR lion[tiab] OR lions[tiab] OR hog[tiab] OR hogs[tiab] OR songbird[tiab] OR songbirds[tiab] OR oreochromis[tiab] OR starling[tiab] OR starlings[tiab] OR caprine[tiab] OR carassius[tiab] OR owl[tiab] OR owls[tiab] OR

		newt[tiab] OR newts[tiab] OR papio[tiab] OR scrofa[tiab] OR hare[tiab] OR hares[tiab] OR gorilla[tiab] OR gorillas[tiab] OR flounder[tiab] OR flounders[tiab] OR goose[tiab] OR herring[tiab] OR herrings[tiab] OR therian[tiab] OR buffaloes[tiab] OR canary[tiab] OR sparrow[tiab] OR sparrows[tiab] OR microtus[tiab] OR octopus[tiab] OR troglodytes[tiab] OR tuna[tiab] OR amphibia[tiab] OR chinchilla[tiab] OR chinchillas[tiab] OR ide[tiab] OR oryzias[tiab] OR cervus[tiab] OR kangaroo[tiab] OR kangaroos[tiab] OR armadillo[tiab] OR armadillos[tiab] OR callithrix[tiab] OR pan troglodytes[tiab] OR saimiri[tiab] OR cichlid[tiab] OR cichlids[tiab] OR donkey[tiab] OR donkeys[tiab] OR bream[tiab] OR char[tiab] OR chars[tiab] OR finch[tiab] OR raccoon[tiab] OR raccoons[tiab] OR bothrops[tiab] OR anguilla[tiab] OR perch[tiab] OR cricetus[tiab] OR seabird[tiab] OR seabirds[tiab] OR buck[tiab] OR bucks[tiab] OR naja[tiab] OR coturnix[tiab] OR salmonids[tiab] OR geese[tiab] OR minnow[tiab] OR minnows[tiab] OR raptor[tiab] OR raptors[tiab] OR merione[tiab] OR meriones[tiab] OR rodentia[tiab] OR elaphus[tiab] OR amniote[tiab] OR amniotes[tiab] OR elasmobranch[tiab] OR emu[tiab] OR emus[tiab] OR peromyscus[tiab] OR hominid[tiab] OR hominids[tiab] OR bubalus[tiab] OR crotalus[tiab] OR gull[tiab] OR gulls[tiab] OR anas[tiab] OR anura[tiab] OR lemur[tiab] OR lemurs[tiab] OR crow[tiab] OR crows[tiab] OR camelus[tiab] OR gibbon[tiab] OR gibbons[tiab] OR waterfowl[tiab] OR parrot[tiab] OR parrots[tiab] OR eels[tiab] OR cob[tiab] OR stickleback[tiab] OR sticklebacks[tiab] OR columba[tiab] OR mesocricetus[tiab] OR ambystoma[tiab] OR raven[tiab] OR ravens[tiab] OR gadus[tiab] OR penguin[tiab] OR penguins[tiab] OR orangutan[tiab] OR orangutans[tiab] OR sturgeon[tiab] OR sturgeons[tiab] OR cuniculus[tiab] OR aves[tiab] OR virginianus[tiab] OR cephalopod[tiab] OR cephalopods[tiab] OR cebus[tiab] OR sparus[tiab] OR tortoise[tiab] OR tortoises[tiab] OR guttata[tiab] OR morhua[tiab] OR unguiculatus[tiab] OR dogfish[tiab] OR vulpes[tiab] OR mallard[tiab] OR mallards[tiab] OR apodemus[tiab] OR alligator[tiab] OR alligators[tiab] OR oryctolagus[tiab] OR llama[tiab] OR llamas[tiab] OR reindeer[tiab] OR mustela[tiab] OR duckling[tiab] OR ducklings[tiab] OR wolves[tiab] OR sander[tiab] OR amazona[tiab] OR zebu[tiab] OR badger[tiab] OR badgers[tiab] OR dove[tiab] OR doves[tiab] OR ictalurus[tiab] OR capra[tiab] OR capras[tiab] OR equus[tiab] OR camelid[tiab] OR camelids[tiab] OR poecilia[tiab] OR mule[tiab] OR mules[tiab] OR perciformes[tiab] OR salvelinus[tiab] OR labrax[tiab] OR cyprinidae[tiab] OR ariidae[tiab] OR crocodile[tiab] OR crocodiles[tiab] OR fundulus[tiab] OR dicentrarchus[tiab] OR clarias[tiab] OR cercopithecus[tiab] OR chiroptera[tiab] OR alpaca[tiab] OR alpacas[tiab] OR pike[tiab] OR pikes[tiab] OR paralichthys[tiab] OR puma[tiab] OR pumas[tiab] OR didelphis[tiab] OR pisces[tiab] OR macropus[tiab] OR triturus[tiab] OR bison[tiab] OR bisons[tiab] OR epinephelus[tiab] OR gasterosteus[tiab] OR panthera[tiab] OR acipenser[tiab] OR mackerel[tiab] OR mackerels[tiab] OR tamarin[tiab] OR tamarins[tiab] OR ostrich[tiab] OR anolis[tiab] OR vervet[tiab] OR vervets[tiab] OR wallaby[tiab] OR glareolus[tiab] OR beaver[tiab] OR beavers[tiab] OR dromedary[tiab] OR catus[tiab] OR killifish[tiab] OR pimephales[tiab] OR promelas[tiab] OR aotus[tiab] OR phoca[tiab] OR panda[tiab] OR pandas[tiab] OR porpoise[tiab] OR porpoises[tiab] OR myotis[tiab] OR yak[tiab] OR yaks[tiab] OR agkistrodon[tiab] OR vipera[tiab] OR otter[tiab] OR otters[tiab] OR turbot[tiab] OR turbots[tiab] OR squamate[tiab] OR carnivora[tiab] OR mullet[tiab] OR mullets[tiab] OR hawk[tiab] OR hawks[tiab] OR taeniopygia[tiab] OR seahorse[tiab] OR seahorses[tiab] OR poecilia reticulata[tiab] OR falcon[tiab] OR falcons[tiab] OR prosimian[tiab] OR prosimians[tiab] OR parus[tiab] OR perca[tiab] OR fingerling[tiab] OR fingerlings[tiab] OR antelope[tiab] OR antelopes[tiab] OR tupaia[tiab] OR passeriformes[tiab] OR sepia[tiab] OR saguinus[tiab] OR coyote[tiab] OR coyotes[tiab] OR pongo[tiab] OR meleagris[tiab] OR reptilia[tiab] OR lepus[tiab] OR psittacine[tiab] OR hagfish[tiab] OR warbler[tiab] OR warblers[tiab] OR russell's viper[tiab] OR russell's vipers[tiab] OR smolt[tiab] OR smolts[tiab] OR budgerigar[tiab] OR sardine[tiab] OR sardines[tiab] OR cavia[tiab] OR cavias[tiab] OR hyla[tiab] OR pleurodeles[tiab] OR siluriformes[tiab] OR great tit[tiab] OR great tits[tiab] OR guppy[tiab] OR bonobo[tiab] OR bonobos[tiab] OR rutilus[tiab] OR trichosurus[tiab] OR muridae[tiab] OR phodopus[tiab] OR channa[tiab] OR squalus[tiab] OR lynx[tiab] OR sturnus[tiab] OR petromyzon[tiab] OR vitulina[tiab] OR monodelphis[tiab] OR cuttlefish[tiab] OR adder[tiab] OR adders[tiab] OR lepomis[tiab] OR canaria[tiab] OR gambusia[tiab] OR guppies[tiab] OR xiphophorus[tiab] OR flatfish[tiab] OR koala[tiab] OR koalas[tiab] OR labeo[tiab] OR stingray[tiab] OR stingrays[tiab] OR chelonia[tiab] OR lampetra[tiab] OR spermophilus[tiab] OR crocodilian[tiab] OR passer domesticus[tiab] OR sciurus[tiab] OR artiodactyla[tiab] OR ranidae[tiab] OR corvus[tiab] OR necturus[tiab] OR platypus[tiab] OR canaries[tiab] OR bovid[tiab] OR lagopus[tiab] OR trimeresurus[tiab] OR gariepinus[tiab] OR marten[tiab] OR martens[tiab] OR drosophilidae[tiab] OR mugil[tiab] OR sunfish[tiab] OR porcellus[tiab] OR cypriniformes[tiab] OR alouatta[tiab] OR scophthalmus[tiab] OR anser[tiab] OR electrophorus[tiab] OR putorius[tiab] OR iguana[tiab] OR iguanas[tiab] OR lama[tiab] OR lamas[tiab] OR takifugu[tiab] OR circus[tiab] OR eptesicus[tiab] OR flycatcher[tiab] OR galago[tiab] OR galagos[tiab] OR trachemys[tiab] OR lungfish[tiab] OR characiformes[tiab] OR shorebird[tiab] OR shorebirds[tiab] OR giraffe[tiab] OR giraffes[tiab] OR micropterus[tiab] OR scyliorhinus[tiab] OR cichlidae[tiab] OR loligo[tiab] OR porcupine[tiab] OR porcupines[tiab] OR chub[tiab] OR chubs[tiab] OR solea[tiab] OR pleuronectes[tiab] OR hylidae[tiab] OR viperidae[tiab] OR echis[tiab] OR sorex[tiab] OR anchovy[tiab] OR lagomorph[tiab] OR ostriches[tiab] OR vulture[tiab] OR vultures[tiab] OR whitefish[tiab] OR araneus[tiab] OR jird[tiab] OR jirds[tiab] OR tern[tiab] OR esox[tiab] OR drake[tiab] OR drakes[tiab] OR elapidae[tiab] OR gallopavo[tiab] OR chordata[tiab] OR myodes[tiab] OR caretta[tiab] OR serinus[tiab] OR grouse[tiab] OR misgurnus[tiab] OR meles[tiab] OR blackbird[tiab] OR blackbirds[tiab] OR coregonus[tiab] OR bobwhite[tiab] OR bobwhites[tiab] OR heteropneustes[tiab] OR mammoth[tiab] OR mammoths[tiab] OR turdus[tiab] OR rhinella[tiab] OR ateles[tiab] OR characidae[tiab] OR clupea[tiab] OR bungarus [tiab] OR brill[tiab] OR struthio camelus[tiab] OR sloth[tiab] OR sloths[tiab] OR pteropus[tiab] OR sculpin[tiab] OR anthropoids[tiab] OR pollock[tiab] OR pollocks[tiab] OR morone[tiab] OR pan paniscus[tiab] OR litoria[tiab] OR chipmunk[tiab] OR chipmunks[tiab] OR balaenoptera[tiab] OR marmota[tiab] OR melopsittacus[tiab] OR hyrax[tiab] OR lemming[tiab] OR lemmings[tiab] OR halibut[tiab] OR hylobates[tiab] OR lates[tiab] OR caiman[tiab] OR caimans[tiab] OR sigmodon[tiab] OR stenella[tiab] OR barbel[tiab] OR barbels[tiab] OR sterna[tiab] OR parakeet[tiab] OR parakeets[tiab] OR phocoena[tiab] OR leptodactylus[tiab] OR canidae[tiab] OR buteo[tiab] OR harengus[tiab] OR gopher[tiab] OR gophers[tiab] OR marmot[tiab] OR marmots[tiab] OR gosling[tiab] OR goslings[tiab] OR platichthys[tiab] OR gar[tiab] OR gars[tiab] OR sebastes[tiab] OR marsupialia[tiab] OR notophthalmus[tiab] OR gazelle[tiab] OR gazelles[tiab] OR insectivora[tiab] OR paridae[tiab] OR felidae[tiab] OR russula[tiab] OR galliformes[tiab] OR bombina[tiab] OR colobus [tiab] OR echidna[tiab] OR echidnas[tiab] OR seabass[tiab] OR syncerus[tiab] OR plaice[tiab] OR blue tit[tiab] OR blue tits[tiab] OR pagrus[tiab] OR catfishes[tiab] OR cetacea[tiab] OR barbus[tiab] OR cygnus[tiab] OR ficedula[tiab] OR chamois[tiab] OR colubridae[tiab] OR perches[tiab] OR coelacanth[tiab] OR fitch[tiab] OR urodela[tiab] OR cynops[tiab] OR martes[tiab] OR halichoerus[tiab] OR aix[tiab] OR salmonidae[tiab] OR leuciscus[tiab] OR magpie[tiab] OR magpies[tiab] OR silurus[tiab] OR whiting[tiab] OR whitings[tiab] OR anseriformes[tiab] OR colinus[tiab] OR rhea[tiab] OR chlorocebus[tiab] OR octodon[tiab] OR acinonyx[tiab] OR mouflon[tiab] OR mouflons[tiab] OR ibex[tiab] OR tetraodon[tiab] OR bufonidae[tiab] OR equidae[tiab] OR jackal[tiab] OR cephalopoda[tiab] OR dendroaspis[tiab] OR glama[tiab] OR muskrat[tiab] OR muskrats[tiab] OR sable[tiab] OR sables[tiab] OR wildebeest[tiab] OR streptopelia[tiab] OR albifrons[tiab] OR vespertilionidae[tiab] OR woodpecker[tiab] OR woodpeckers[tiab] OR muntjac[tiab] OR muntjacs[tiab] OR archosaur[tiab] OR branta[tiab] OR cricetulus[tiab] OR megalobrama[tiab] OR poeciliidae[tiab] OR desmodus[tiab] OR snakehead[tiab] OR snakeheads[tiab] OR tench[tiab] OR teal[tiab] OR teals[tiab] OR bandicoot[tiab] OR bandicoots[tiab] OR apteronotus[tiab] OR phyllostomidae[tiab] OR crocidura[tiab] OR buzzard[tiab] OR buzzards[tiab] OR larimichthys[tiab] OR cercocebus[tiab] OR pipistrellus[tiab] OR erithacus[tiab] OR impala[tiab] OR impalas[tiab] OR rousettus[tiab] OR haddock[tiab] OR haddocks[tiab] OR tinca[tiab] OR ratite[tiab] OR calidris[tiab] OR cynoglossus[tiab] OR hypophthalmichthys[tiab] OR bullock[tiab] OR bullocks[tiab] OR dromedaries[tiab] OR alectoris[tiab] OR filly[tiab] OR

		salamandra[tiab] OR cingulata[tiab] OR bitis[tiab] OR grus[tiab] OR ammodytes[tiab] OR macaw[tiab] OR macaws[tiab] OR hypoleuca[tiab] OR sapajus[tiab] OR cyprinodontiformes[tiab] OR hippopotamus[tiab] OR pelophylax[tiab] OR capybara[tiab] OR capybaras[tiab] OR weasel[tiab] OR weasels[tiab] OR cairina[tiab] OR cynomys[tiab] OR lutra[tiab] OR cockatoo[tiab] OR cockatoos[tiab] OR lachesis[tiab] OR lagomorpha[tiab] OR rupicapra[tiab] OR daboia[tiab] OR orang utan[tiab] OR orang utans[tiab] OR platyrrhini[tiab] OR charadriiformes[tiab] OR micrurus[tiab] OR psittaciformes[tiab] OR spalax[tiab] OR loris[tiab] OR mustelidae[tiab] OR sylvilagus[tiab] OR vitticeps[tiab] OR cockatiel[tiab] OR mustelus[tiab] OR cottus[tiab] OR erythrocebus[tiab] OR dipodomys[tiab] OR platessa[tiab] OR callicebus[tiab] OR loricariidae[tiab] OR catostomus[tiab] OR cuneata[tiab] OR cyanistes[tiab] OR cyprinodon[tiab] OR sigmodontinae[tiab] OR elasmobranchii[tiab] OR trichechus[tiab] OR sauropsid[tiab] OR xenarthra[tiab] OR dormouse[tiab] OR perissodactyla[tiab] OR nautilus[tiab] OR cirrhinus[tiab] OR gulo[tiab] OR tragelaphus[tiab] OR merula[tiab] OR numida[tiab] OR sciaenidae[tiab] OR cerastes[tiab] OR sciuridae[tiab] OR gibbosus[tiab] OR octopuses[tiab] OR eland[tiab] OR elands[tiab] OR phyllomedusa[tiab] OR pogona[tiab] OR walrus[tiab] OR agamidae[tiab] OR leptodactylidae[tiab] OR ridibundus[tiab] OR leontopithecus[tiab] OR anteater[tiab] OR anteaters[tiab] OR pelodiscus[tiab] OR cebidae[tiab] OR columbianus[tiab] OR pelteobagrus fulvidraco[tiab] OR hominoidea[tiab] OR mandrillus[tiab] OR zonotrichia leucophrys[tiab] OR agama[tiab] OR gobiocypris[tiab] OR bearded dragon[tiab] OR bearded dragons[tiab] OR sarotherodon[tiab] OR talpa[tiab] OR discoglossus[tiab] OR hagfishes[tiab] OR sphenodon[tiab] OR gudgeon[tiab] OR amphiuma[tiab] OR aythya[tiab] OR tenrec[tiab] OR tenrec[tiab] OR hominidae[tiab] OR risoria[tiab] OR salamandridae[tiab] OR camelidae[tiab] OR columbiformes[tiab] OR latimeria[tiab] OR plover[tiab] OR plovers[tiab] OR afrotheria[tiab] OR falco sparverius[tiab] OR polecat[tiab] OR polecats[tiab] OR crotalinae[tiab] OR salvadora[tiab] OR tarsier[tiab] OR lucioperca[tiab] OR anchovies[tiab] OR lungfishes[tiab] OR terrapin[tiab] OR dromaius novaehollandiae[tiab] OR lateolabrax[tiab] OR eigenmannia[tiab] OR pelamis[tiab] OR theropithecus[tiab] OR murinae[tiab] OR gander[tiab] OR gymnotus[tiab] OR pseudacris[tiab] OR gymnophiona[tiab] OR gymnotiformes[tiab] OR laticauda[tiab] OR falconiformes[tiab] OR dugong[tiab] OR dugongs[tiab] OR pintail[tiab] OR pintails[tiab] OR rook[tiab] OR rooks[tiab] OR lasiurus[tiab] OR catshark[tiab] OR catsharks[tiab] OR micropogonias[tiab] OR red junglefowl[tiab] OR paddlefish[tiab] OR ophiophagus[tiab] OR hollandicus[tiab] OR nymphicus[tiab] OR pimelodidae[tiab] OR aepyceros[tiab] OR cobitidae[tiab] OR strigiformes[tiab] OR cobitis[tiab] OR dormice[tiab] OR alytes[tiab] OR calloselasma[tiab] OR guanaco[tiab] OR phasianidae[tiab] OR round goby[tiab] OR trichogaster[tiab] OR catarrhini[tiab] OR eelpout[tiab] OR eelpouts[tiab] OR galaxias[tiab] OR gaur[tiab] OR pungitius[tiab] OR suslik[tiab] OR susliks[tiab] OR flatfishes[tiab] OR percidae[tiab] OR caprinae[tiab] OR todarodes[tiab] OR osmerus[tiab] OR ameiurus[tiab] OR anthropoidea[tiab] OR castor canadensis[tiab] OR pouting[tiab] OR poutings[tiab] OR tetraodontiformes[tiab] OR arvicolinae[tiab] OR siamang[tiab] OR siamangs[tiab] OR castor fiber[tiab] OR nomascus[tiab] OR red knot[tiab] OR red knots[tiab] OR syngnathidae[tiab] OR iguanidae[tiab] OR eretmochelys[tiab] OR ursidae[tiab] OR callimico[tiab] OR columbidae[tiab] OR microhylidae[tiab] OR anaxyrus[tiab] OR menidia[tiab] OR pipistrelle[tiab] OR greylag[tiab] OR pipidae[tiab] OR scandentia[tiab] OR bowfin[tiab] OR bowfins[tiab] OR dendrobatidae[tiab] OR zenaida[tiab] OR bushbaby[tiab] OR harrier[tiab] OR harriers[tiab] OR macropodidae[tiab] OR pygerythrus[tiab] OR clupeidae[tiab] OR odorrana[tiab] OR corvidae[tiab] OR jerboa[tiab] OR jerboas[tiab] OR canutus[tiab] OR hylobatidae[tiab] OR clupeiformes[tiab] OR great cormorant[tiab] OR great cormorants[tiab] OR scorpaeniformes[tiab] OR chondrostean[tiab] OR garfish[tiab] OR proboscidea[tiab] OR psetta[tiab] OR diapsid[tiab] OR serotinus[tiab] OR tetrao[tiab] OR walruses[tiab] OR carcharhiniformes[tiab] OR leucoraja[tiab] OR pumpkinseed[tiab] OR dosidicus[tiab] OR acipenseriformes[tiab] OR daubentonii[tiab] OR emberizidae[tiab] OR gadiformes[tiab] OR hyraxes[tiab] OR stizostedion[tiab] OR wolverine[tiab] OR wolverines[tiab] OR lissotriton[tiab] OR acanthurus[tiab] OR centrarchidae[tiab] OR gloydius[tiab] OR laurasiatheria[tiab] OR limosa[tiab] OR psittacula[tiab] OR leporidae[tiab] OR proteidae[tiab] OR zander[tiab] OR zanders[tiab] OR arapaima[tiab] OR bagridae[tiab] OR cyprinodontidae[tiab] OR mithun[tiab] OR pandion[tiab] OR jackdaw[tiab] OR jackdaws[tiab] OR procyonidae[tiab] OR carus[tiab] OR jaculus[tiab] OR salmoniformes[tiab] OR common sole[tiab] OR common soles[tiab] OR protobothrops[tiab] OR calamita[tiab] OR brachyteles[tiab] OR trionyx[tiab] OR turdidae[tiab] OR boidae[tiab] OR luscinia[tiab] OR pugnax[tiab] OR euarchontoglires[tiab] OR saithe[tiab] OR saithes[tiab] OR symphalangus[tiab] OR aardvark[tiab] OR aardvarks[tiab] OR oystercatcher[tiab] OR oystercatchers[tiab] OR arius[tiab] OR corydoras[tiab] OR poacher[tiab] OR poachers[tiab] OR aurochs[tiab] OR cebuella[tiab] OR crecca[tiab] OR lemuridae[tiab] OR sirenia[tiab] OR lemmus[tiab] OR perdix[tiab] OR glires[tiab] OR lepidosaur[tiab] OR muskox[tiab] OR deinagkistrodon[tiab] OR pholidota[tiab] OR holocephali[tiab] OR cercopithecinae[tiab] OR clariidae[tiab] OR agapornis[tiab] OR doryteuthis[tiab] OR tyrannidae[tiab] OR dicroglossidae[tiab] OR godwit[tiab] OR godwits[tiab] OR monedula[tiab] OR pongidae[tiab] OR atheriniformes[tiab] OR colobinae[tiab] OR lophocebus[tiab] OR atelidae[tiab] OR cottidae[tiab] OR leucopsis[tiab] OR acanthuridae[tiab] OR didelphimorphia[tiab] OR elver[tiab] OR elvers[tiab] OR lapponica[tiab] OR dermoptera[tiab] OR “european hake”[tiab] OR “european hakes”[tiab] OR gerbillinae[tiab] OR banteng[tiab] OR hartebeest[tiab] OR hartebeests[tiab] OR hogget[tiab] OR haematopus[tiab] OR anguis fragilis[tiab] OR grey heron[tiab] OR grey herons[tiab] OR “blue whiting”[tiab] OR “blue whitings”[tiab] OR furnariidae[tiab] OR macrovipera[tiab] OR esocidae[tiab] OR lapwing[tiab] OR lapwings[tiab] OR mylopharyngodon[tiab] OR wallabia[tiab] OR beloniformes[tiab] OR potoroo[tiab] OR potoroos[tiab] OR athene noctua[tiab] OR pleuronectidae[tiab] OR bushbabies[tiab] OR muscicapidae[tiab] OR alligatoridae[tiab] OR fuligula[tiab] OR bush baby[tiab] OR guineafowl[tiab] OR spoonbill[tiab] OR spoonbills[tiab] OR viverridae[tiab] OR catostomidae[tiab] OR zebrafishes[tiab] OR ibexes[tiab] OR vendace[tiab] OR estrildidae[tiab] OR monotremata[tiab] OR sepiella[tiab] OR ambystomatidae[tiab] OR shelduck[tiab] OR shelducks[tiab] OR treeshrew[tiab] OR treeshrews[tiab] OR hoplobatrachus[tiab] OR pochard[tiab] OR hoolock[tiab] OR hoolocks[tiab] OR lynxes[tiab] OR antilope[tiab] OR antilopes[tiab] OR blackbuck[tiab] OR blackbucks[tiab] OR cricetinae[tiab] OR paramisgurnus[tiab] OR skylark[tiab] OR skylarks[tiab] OR soleidae[tiab] OR allobates[tiab] OR northern wheatear[tiab] OR northern wheatears[tiab] OR pitheciidae[tiab] OR takin[tiab] OR theria[tiab] OR vanellus[tiab] OR galaxiidae[tiab] OR lorisidae[tiab] OR ostralegus[tiab] OR palaeognathae[tiab] OR stone loach[tiab] OR alauda[tiab] OR callitrichinae[tiab] OR caniformia[tiab] OR duttaphrynus[tiab] OR ictaluridae[tiab] OR osteoglossiformes[tiab] OR poultries[tiab] OR curema[tiab] OR ruddy turnstone[tiab] OR ruddy turnstones[tiab] OR sheatfish[tiab] OR sunfishes[tiab] OR centropomidae[tiab] OR hemachatus[tiab] OR platalea[tiab] OR thamnophilidae[tiab] OR song thrush[tiab] OR atherinopsidae[tiab] OR siluridae[tiab] OR tadorna[tiab] OR chroicocephalus[tiab] OR ermine[tiab] OR ermines[tiab] OR gavialis[tiab] OR ruff[tiab] OR tupaiidae[tiab] OR diprotodontia[tiab] OR hyaenidae[tiab] OR antilopinae[tiab] OR crocodylidae[tiab] OR herpestidae[tiab] OR hippopotamidae[tiab] OR northern shoveler[tiab] OR round gobies[tiab] OR cheirogaleidae[tiab] OR indriidae[tiab] OR fundulidae[tiab] OR pythonidae[tiab] OR rhynchocephalia[tiab] OR anodorhynchus[tiab] OR red-backed shrike[tiab] OR red-backed shrikes[tiab] OR triakidae[tiab] OR phalangeridae[tiab] OR aoudad[tiab] OR boreoeutheria[tiab] OR eurasian jay[tiab] OR eurasian jays[tiab] OR feliformia[tiab] OR haplorhini[tiab] OR osteoglossidae[tiab] OR paenungulata[tiab] OR struthioniformes[tiab] OR ferina[tiab] OR sanderling[tiab] OR sanderlings[tiab] OR spheniscidae[tiab] OR cuttlefishes[tiab] OR cygnet[tiab] OR dasycneme[tiab] OR gadwall[tiab] OR gadwalls[tiab] OR pelobates fuscus[tiab] OR wryneck[tiab] OR wrynecks[tiab] OR afrosoricida[tiab] OR culaea[tiab] OR “dover sole”[tiab] OR “dover soles”[tiab] OR paralichthyidae[tiab] OR passeridae[tiab] OR osteolaemus[tiab] OR song thrushes[tiab] OR bluethroat[tiab] OR bluethroats[tiab] OR hydrophiidae[tiab] OR megrim[tiab] OR mephitidae[tiab] OR strepsirhini[tiab] OR tomistoma[tiab] OR epidalea[tiab] OR osmeriformes[tiab] OR bush babies[tiab] OR tarsiiform[tiab] OR atelinae[tiab] OR

		bufotes[tiab] OR eurasian coot[tiab] OR eurasian coots[tiab] OR galagidae[tiab] OR geopelia[tiab] OR philomachus[tiab] OR tubulidentata[tiab] OR bombinatoridae[tiab] OR pelobatidae[tiab] OR tachysurus[tiab] OR ailuridae[tiab] OR woodlark[tiab] OR woodlarks[tiab] OR alcelaphinae[tiab] OR redshank[tiab] OR redshanks[tiab] OR salientia[tiab] OR sand smelt[tiab] OR sand smelts[tiab] OR woodmice[tiab] OR woodmouse[tiab] OR dasyproctidae[tiab] OR eurasian wigeon[tiab] OR eurasian wigeons[tiab] OR garganey[tiab] OR garganeys[tiab] OR “lemon sole”[tiab] OR “lemon soles”[tiab] OR “common dab”[tiab] OR “common dabs”[tiab] OR graylag[tiab] OR graylags[tiab] OR leucorodia[tiab] OR osphronemidae[tiab] OR bewickii[tiab] OR common moorhen[tiab] OR common moorhens[tiab] OR decapodiformes[tiab] OR gobbler[tiab] OR gobblers[tiab] OR odontophoridae[tiab] OR paddlefishes[tiab] OR eutheria[tiab] OR salmonine[tiab] OR esociformes[tiab] OR eurasian woodcock[tiab] OR eurasian woodcocks[tiab] OR “european smelt”[tiab] OR “european smelts”[tiab] OR goldfishes[tiab] OR tenches[tiab] OR tyranni[tiab] OR common chaffinch[tiab] OR common chaffinchs[tiab] OR common redstart[tiab] OR common redstarts[tiab] OR “common roach”[tiab] OR “common roachs”[tiab] OR great knot[tiab] OR great knots[tiab] OR potoroidae[tiab] OR alytidae[tiab] OR coregonine[tiab] OR dipteral[tiab] OR leveret[tiab] OR poeciliopsis gracilis[tiab] OR amphiumidae[tiab] OR batrachoidiformes[tiab] OR bighead goby[tiab] OR heteropneustidae[tiab] OR lullula[tiab] OR “norway pout”[tiab] OR “norway pouts”[tiab] OR sipunculida[tiab] OR dogfishes[tiab] OR sebastidae[tiab] OR tarsiidae[tiab] OR alethinophidia[tiab] OR “common nase”[tiab] OR “common nases”[tiab] OR “common sandpiper”[tiab] OR “common sandpipers”[tiab] OR eurasian blackcap[tiab] OR eurasian blackcaps[tiab] OR pterocnemia[tiab] OR syngnathiformes[tiab] OR common chaffinches[tiab] OR eupleridae[tiab] OR octopodiformes[tiab] OR phascolarctidae[tiab] OR scophthalmidae[tiab] OR “starry smooth-hound”[tiab] OR “starry smooth-hounds”[tiab] OR whitefishes[tiab] OR cuniculidae[tiab] OR “european sprat”[tiab] OR “european sprats”[tiab] OR “rosy bitterling”[tiab] OR “rosy bitterlings”[tiab] OR “common dace”[tiab] OR “common daces”[tiab] OR “lesser weever”[tiab] OR “lesser weevers”[tiab] OR scaldfish[tiab] OR water rail[tiab] OR water rails[tiab] OR alouattinae[tiab] OR centrarchiformes[tiab] OR “common whitethroat”[tiab] OR “common whitethroats”[tiab] OR gavialidae[tiab] OR “grey gurnard”[tiab] OR “grey gurnards”[tiab] OR lateolabracidae[tiab] OR rheiformes[tiab] OR “tub gurnard”[tiab] OR “tub gurnards”[tiab] OR “common chiffchaff”[tiab] OR “common chiffchaffs”[tiab] OR garfishes[tiab] OR “lesser whitethroat”[tiab] OR “lesser whitethroats”[tiab] OR myoxidae[tiab] OR seabasses[tiab] OR spariformes[tiab] OR umbridae[tiab] OR yellow boxfish[tiab] OR anabantiformes[tiab] OR aotidae[tiab] OR “common bleak”[tiab] OR “common bleaks”[tiab] OR “common rudd”[tiab] OR “common rudds”[tiab] OR greater pipefish[tiab] OR hapale[tiab] OR nandiniidae[tiab] OR “stone loaches”[tiab] OR whinchat[tiab] OR whinchats[tiab] OR acanthuriformes[tiab] OR brotula barbata[tiab] OR “common ling”[tiab] OR “common lings”[tiab] OR “common roaches”[tiab] OR cottonrat[tiab] OR cottonrats[tiab] OR douroucoulis[tiab] OR dromaiidae[tiab] OR fitches[tiab] OR fitchew[tiab] OR galaxiiformes[tiab] OR laprine[tiab] OR saimiriinae[tiab] OR solenette[tiab] OR tarsii[tiab] OR tompot blenny[tiab] OR “common dragonet”[tiab] OR “common dragonets”[tiab] OR “longspined bullhead”[tiab] OR “longspined bullheads”[tiab] OR monotremate[tiab] OR monotremates[tiab] OR pempheriformes[tiab] OR perdicinae[tiab] OR presbytini[tiab] OR smegmamorpha[tiab] OR “bighead gobies”[tiab] OR “carangaria incertae sedis”[tiab] OR coiidae[tiab] OR fivebeard rockling[tiab] OR foulmart[tiab] OR foumart[tiab] OR grasskeet[tiab] OR “greater pipefishes”[tiab] OR ibices[tiab] OR millionfish[tiab] OR muguliformes[tiab] OR “norwegian topknot”[tiab] OR peewit[tiab] OR “red sea sailfin tang”[tiab] OR rupicapras[tiab] OR sheatfishes[tiab] OR “tompot blennies”[tiab] OR “twait shad”[tiab] OR “yellow boxfishes”[tiab]) NOT medline[sb])
#5	Cardiac AND repair AND ECM and animals 3,034 hits	#1 AND #2 AND #3 AND #4
#6	Remove reviews 2,448 hits	#5 NOT review[ptyp]
#7	*Retrieve reviews for separate screening* 586 hits	#5 AND review[ptyp]

Supplementary File 2 – full search strings for SCOPUS 28-06-2024

SCOPUS	28-06-2024	1679 hits without reviews
#1	Heart injury<br><br>Hits:<br>1,997,066	(((TITLE-ABS("cardiac fibrosis") OR TITLE-ABS(heart) OR TITLE-ABS(hearts) OR TITLE-ABS(cardiac) OR TITLE-ABS(myocardial) OR TITLE-ABS(myocardium) OR TITLE-ABS(myocardia) OR TITLE-ABS(myocard)) OR ((TITLE-ABS(ischemia) OR TITLE-ABS(ischaemia) OR TITLE-ABS(ischemic) OR TITLE-ABS(ischaemic) OR TITLE-ABS(infarct) OR TITLE-ABS(infarcts) OR TITLE-ABS(infarction) OR TITLE-ABS(infarctions) OR TITLE-ABS(infarcted) OR TITLE-ABS("reperfusion injury") OR TITLE-ABS("reperfusion injuries") OR TITLE-ABS(IR) OR TITLE-ABS(IRI) OR TITLE-ABS(occlusion) OR TITLE-ABS(failure) OR TITLE-ABS(decompensation) OR TITLE-ABS(insufficiency) OR TITLE-ABS(incompetence) OR TITLE-ABS(overload) OR TITLE-ABS(transplantation) OR TITLE-ABS(transplantations) OR TITLE-ABS(transplant) OR TITLE-ABS(transplants) OR TITLE-ABS(graft) OR TITLE-ABS(grafts)) AND (TITLE-ABS(heart) OR TITLE-ABS(hearts) OR TITLE-ABS(cardiac) OR TITLE-ABS(myocardial) OR TITLE-ABS(myocardium) OR TITLE-ABS(myocardia) OR TITLE-ABS(myocard)))) AND NOT (TITLE-ABS("Heart Valves") OR TITLE-ABS("Heart Valve") OR TITLE-ABS("Aortic Valves") OR TITLE-ABS("Aortic Valve") OR TITLE-ABS("Chordae Tendineae") OR TITLE-ABS("Mitral Valves") OR TITLE-ABS("Mitral Valve") OR TITLE-ABS("Pulmonary Valves") OR TITLE-ABS("Pulmonary Valve") OR TITLE-ABS("Tricuspid Valves") OR TITLE-ABS("Tricuspid Valve") OR TITLE-ABS("Cardiac Valves") OR TITLE-ABS("Cardiac Valve"))
#2	Repair<br><br>Hits:<br>1,221,495	(TITLE-ABS(regeneration) OR TITLE-ABS(regenerations) OR TITLE-ABS(regenerated) OR TITLE-ABS(regenerative) OR TITLE-ABS("wound healing") OR TITLE-ABS("wound healings") OR TITLE-ABS(renewal) OR TITLE-ABS(repair) OR TITLE-ABS("Tissue Engineering") OR TITLE-ABS("Tissue Scaffolds") OR TITLE-ABS("Tissue Scaffold"))
#2	ECM<br><br>Hits:<br>642,217	(TITLE-ABS("Extracellular Matrix Protein") OR TITLE-ABS("Extracellular Matrix Proteins") OR TITLE-ABS("Extracellular Matrix") OR TITLE-ABS("Extracellular Matrices") OR TITLE-ABS(ECM) OR TITLE-ABS(ECMs) OR TITLE-ABS("Decellularized Extracellular Matrix") OR TITLE-ABS("Decellularized Extracellular Matrices") OR TITLE-ABS("Decellularized ECM") OR TITLE-ABS("Decellularized ECMs") OR TITLE-ABS(Glycosaminoglycans) OR TITLE-ABS(Glycosaminoglycan) OR TITLE-ABS(Chondroitin) OR TITLE-ABS("Chondroitin Sulfates") OR TITLE-ABS("Chondroitin Sulfate") OR TITLE-ABS("Dermatan Sulfates") OR TITLE-ABS("Dermatan Sulfate") OR TITLE-ABS(Heparin) OR TITLE-ABS(Dalteparin) OR TITLE-ABS(Enoxaparin) OR TITLE-ABS(Nadroparin) OR TITLE-ABS(Tinzaparin) OR TITLE-ABS(Heparinoids) OR TITLE-ABS("Heparitin Sulfate") OR TITLE-ABS("Heparan Sulfate Proteoglycans") OR TITLE-ABS("Hyaluronic Acid") OR TITLE-ABS("Keratan Sulfate") OR TITLE-ABS("Biocompatible Materials") OR TITLE-ABS(Proteoglycans) OR TITLE-ABS(Proteoglycan) OR TITLE-ABS("Activated-Leukocyte Cell Adhesion Molecule") OR TITLE-ABS("ADAMTS Proteins") OR TITLE-ABS("ADAMTS1 Protein") OR TITLE-ABS("ADAMTS13 Protein") OR TITLE-ABS("ADAMTS4 Protein") OR TITLE-ABS("ADAMTS5 Protein") OR TITLE-ABS("ADAMTS7 Protein") OR TITLE-ABS("ADAMTS9 Protein") OR TITLE-ABS(Aggrecan) OR TITLE-ABS(Aggrecans) OR TITLE-ABS("Cartilage Oligomeric Matrix Protein") OR TITLE-ABS("Cartilage Oligomeric Matrix Proteins") OR TITLE-ABS("CCN Intercellular Signaling Protein") OR TITLE-ABS("CCN Intercellular Signaling Proteins") OR TITLE-ABS("Connective Tissue Growth Factor") OR TITLE-ABS("Connective Tissue Growth Factors") OR TITLE-ABS("Cysteine-Rich Protein 61") OR TITLE-ABS("Nephroblastoma Overexpressed Protein") OR TITLE-ABS(Collagen) OR TITLE-ABS(Collagens) OR TITLE-ABS("Fibrillar Collagens") OR TITLE-ABS("Non-Fibrillar Collagens") OR TITLE-ABS(Procollagen) OR TITLE-ABS(Tropocollagen) OR TITLE-ABS(Elastin) OR TITLE-ABS(Tropoelastin) OR TITLE-ABS(Fibrillins) OR TITLE-ABS(Fibrillin-1) OR TITLE-ABS(Fibrillin-2) OR TITLE-ABS(Fibronectins) OR TITLE-ABS("Integrin-Binding Sialoprotein") OR TITLE-ABS(Laminin) OR TITLE-ABS("Latent TGF-beta Binding Proteins") OR TITLE-ABS("Matrilin Proteins") OR TITLE-ABS(Netrins) OR TITLE-ABS(Netrin-1) OR TITLE-ABS(Osteopontin) OR TITLE-ABS("Reelin Protein") OR TITLE-ABS("Small Leucine-Rich Proteoglycans") OR TITLE-ABS(Biglycan) OR TITLE-ABS(Decorin) OR TITLE-ABS(Fibromodulin) OR TITLE-ABS(Lumican) OR TITLE-ABS(Tenascin) OR TITLE-ABS(Versicans) OR TITLE-ABS(Vitronectin) OR TITLE-ABS("Chondroitin Sulfate Proteoglycans") OR TITLE-ABS(Aggrecans) OR TITLE-ABS(Versicans) OR TITLE-ABS(Versican)

		OR TITLE-ABS("Small Leucine-Rich Proteoglycans") OR TITLE-ABS(Biglycan) OR TITLE-ABS(Decorin) OR TITLE-ABS(Fibromodulin) OR TITLE-ABS(Lumican) OR TITLE-ABS("Hyaluronan Receptors") OR TITLE-ABS("Hyaluronan Receptor") OR TITLE-ABS(Glypicans) OR TITLE-ABS(Syndecans) OR TITLE-ABS(Syndecan) OR TITLE-ABS(Syndecan-1) OR TITLE-ABS(Syndecan-2) OR TITLE-ABS(Syndecan-3) OR TITLE-ABS(Syndecan-4) OR TITLE-ABS(Syndecan1) OR TITLE-ABS(Syndecan2) OR TITLE-ABS(Syndecan3) OR TITLE-ABS(Syndecan4) OR TITLE-ABS(Glypican) OR TITLE-ABS(Glypican-5) OR TITLE-ABS(Glypican5) OR TITLE-ABS(Glypican-3) OR TITLE-ABS(Glypican3) OR TITLE-ABS(Glypican-4) OR TITLE-ABS(Glypican4) OR TITLE-ABS(Glypican-1) OR TITLE-ABS(Glypican1) OR TITLE-ABS(Glypican-2) OR TITLE-ABS(Glypican2) OR TITLE-ABS(Biglycan) OR TITLE-ABS(Decorin) OR TITLE-ABS(Fibromodulin) OR TITLE-ABS(Lumican) OR TITLE-ABS("Fibrillar Collagen") OR TITLE-ABS("Collagen Type I") OR TITLE-ABS("Collagen Type II") OR TITLE-ABS("Collagen Type III") OR TITLE-ABS("Collagen Type V") OR TITLE-ABS("Collagen Type XI") OR TITLE-ABS("Non-Fibrillar Collagen") OR TITLE-ABS("Collagen Type IV") OR TITLE-ABS("Collagen Type VI") OR TITLE-ABS("Collagen Type VII") OR TITLE-ABS("Collagen Type VIII") OR TITLE-ABS("Collagen Type X") OR TITLE-ABS("Collagen Type XIII") OR TITLE-ABS("Collagen Type XVIII +") OR TITLE-ABS("Fibril-Associated Collagens") OR TITLE-ABS("Collagen Type IX") OR TITLE-ABS("Collagen Type XII") OR TITLE-ABS(Endostatins) OR TITLE-ABS(Endostatin) OR TITLE-ABS(Fibrillin) OR TITLE-ABS(Fibronectins) OR TITLE-ABS(Fibronectin))
#4	Animal models<br><br>Hits: 10,604,663	(((TITLE-ABS(rat) OR TITLE-ABS(rats) OR TITLE-ABS(animal) OR TITLE-ABS(animals) OR TITLE-ABS(mice) OR TITLE-ABS("in vivo") OR TITLE-ABS(mouse) OR TITLE-ABS(rabbit) OR TITLE-ABS(rabbits) OR TITLE-ABS(murine) OR TITLE-ABS(pig) OR TITLE-ABS(pigs) OR TITLE-ABS(dog) OR TITLE-ABS(dogs) OR TITLE-ABS(bovine) OR TITLE-ABS(fish) OR TITLE-ABS(vertebrate) OR TITLE-ABS(vertebrates) OR TITLE-ABS(cat) OR TITLE-ABS(cats) OR TITLE-ABS(rodent) OR TITLE-ABS(rodents) OR TITLE-ABS(mammal) OR TITLE-ABS(mammals) OR TITLE-ABS(chicken) OR TITLE-ABS(chickens) OR TITLE-ABS(monkey) OR TITLE-ABS(monkeys) OR TITLE-ABS(sheep) OR TITLE-ABS(canine) OR TITLE-ABS(canines) OR TITLE-ABS(porcine) OR TITLE-ABS(cattle) OR TITLE-ABS(bird) OR TITLE-ABS(birds) OR TITLE-ABS(hamster) OR TITLE-ABS(hamsters) OR TITLE-ABS(primate) OR TITLE-ABS(primates) OR TITLE-ABS(cow) OR TITLE-ABS(cows) OR TITLE-ABS(chick) OR TITLE-ABS(horse) OR TITLE-ABS(horses) OR TITLE-ABS(avian) OR TITLE-ABS(avians) OR TITLE-ABS(calf) OR TITLE-ABS(swine) OR TITLE-ABS(swines) OR TITLE-ABS(xenopus) OR TITLE-ABS(turkeys) OR TITLE-ABS(bear) OR TITLE-ABS(bears) OR TITLE-ABS(frog) OR TITLE-ABS(frogs) OR TITLE-ABS(zebrafish) OR TITLE-ABS(goat) OR TITLE-ABS(goats) OR TITLE-ABS(equine) OR TITLE-ABS(calves) OR TITLE-ABS(poultry) OR TITLE-ABS(macaque) OR TITLE-ABS(macaques) OR TITLE-ABS(mole) OR TITLE-ABS(moles) OR TITLE-ABS(ovine) OR TITLE-ABS(lamb) OR TITLE-ABS(lambs) OR TITLE-ABS(fishes) OR TITLE-ABS(diptera) OR TITLE-ABS(amphibian) OR TITLE-ABS(amphibians) OR TITLE-ABS(snake) OR TITLE-ABS(snakes) OR TITLE-ABS(ruminant) OR TITLE-ABS(ruminants) OR TITLE-ABS(hen) OR TITLE-ABS(hens) OR TITLE-ABS(piglet) OR TITLE-ABS(piglets) OR TITLE-ABS(feline) OR TITLE-ABS(felines) OR TITLE-ABS(simian) OR TITLE-ABS(simians) OR TITLE-ABS(laevis) OR TITLE-ABS(trout) OR TITLE-ABS(trouts) OR TITLE-ABS(teleost) OR TITLE-ABS(teleosts) OR TITLE-ABS(salmon) OR TITLE-ABS(salmons) OR TITLE-ABS(seal) OR TITLE-ABS(seals) OR TITLE-ABS(bull) OR TITLE-ABS(bulls) OR TITLE-ABS(ewe) OR TITLE-ABS(ewes) OR TITLE-ABS(hedgehog) OR TITLE-ABS(hedgehogs) OR TITLE-ABS(macaca) OR TITLE-ABS(macacas) OR TITLE-ABS(proteus) OR TITLE-ABS(pigeon) OR TITLE-ABS(pigeons) OR TITLE-ABS(bat) OR TITLE-ABS(bats) OR TITLE-ABS(duck) OR TITLE-ABS(ducks) OR TITLE-ABS(chimpanzee) OR TITLE-ABS(chimpanzees) OR TITLE-ABS(baboon) OR TITLE-ABS(baboons) OR TITLE-ABS(deer) OR TITLE-ABS(rana) OR TITLE-ABS(ranas) OR TITLE-ABS(carp) OR TITLE-ABS(carps) OR TITLE-ABS(heifer) OR TITLE-ABS(swallow) OR TITLE-ABS(swallows) OR TITLE-ABS(lizard) OR TITLE-ABS(lizards) OR TITLE-ABS(canis) OR TITLE-ABS(sow) OR TITLE-ABS(sows) OR TITLE-ABS(cynomolgus) OR TITLE-ABS(quail) OR TITLE-ABS(quails) OR TITLE-ABS(reptile) OR TITLE-ABS(reptiles) OR TITLE-ABS(turtle) OR TITLE-ABS(turtles) OR TITLE-ABS(buffalo) OR TITLE-ABS(gerbil) OR TITLE-ABS(gerbils) OR TITLE-ABS(boar) OR TITLE-ABS(boars) OR TITLE-ABS(squirrel) OR TITLE-ABS(squirrels) OR TITLE-ABS(oncorhynchus) OR TITLE-ABS(mus) OR TITLE-ABS(toad) OR TITLE-ABS(toads) OR TITLE-ABS(fowl) OR TITLE-ABS(fowls) OR TITLE-ABS(rerio) OR TITLE-ABS(danio) OR TITLE-ABS(ara) OR TITLE-ABS(aras) OR TITLE-ABS(musculus) OR TITLE-ABS(tadpole) OR TITLE-ABS(tadpoles) OR TITLE-ABS(mulatta) OR TITLE-ABS(salmo) OR TITLE-ABS(ram) OR TITLE-ABS(eagle) OR TITLE-ABS(eagles) OR TITLE-ABS(ferret) OR TITLE-ABS(ferrets) OR TITLE-ABS(goldfish) OR TITLE-ABS(catfish) OR TITLE-ABS(whale) OR TITLE-ABS(whales) OR TITLE-ABS(fox) OR TITLE-ABS(foxes) OR TITLE-ABS(ape) OR TITLE-ABS(apes) OR TITLE-ABS(elephant) OR TITLE-ABS(elephants) OR TITLE-ABS(bos) OR TITLE-ABS(marmoset) OR TITLE-ABS(marmosets) OR TITLE-ABS(cod) OR TITLE-ABS(cods) OR TITLE-ABS(shark) OR TITLE-ABS(sharks) OR TITLE-ABS(wolf) OR TITLE-ABS(eel) OR TITLE-ABS(eels) OR TITLE-ABS(auratus) OR TITLE-ABS(rattus) OR TITLE-ABS(zebra) OR TITLE-ABS(zebras) OR TITLE-ABS(tilapia) OR TITLE-ABS(tilapias) OR TITLE-ABS(gilt) OR TITLE-ABS(camel) OR TITLE-ABS(camels) OR TITLE-ABS(squid) OR TITLE-ABS(gallus) OR TITLE-ABS(marsupial) OR TITLE-ABS(marsupials) OR TITLE-ABS(vole) OR TITLE-ABS(voles) OR TITLE-ABS(fascicularis) OR TITLE-ABS(ovis) OR TITLE-ABS(salmonid) OR TITLE-ABS(salmonids) OR TITLE-ABS(tiger) OR TITLE-ABS(tigers) OR TITLE-ABS(dolphin) OR TITLE-ABS(dolphins) OR TITLE-ABS(robin) OR TITLE-ABS(robins) OR TITLE-ABS(carpio) OR TITLE-ABS(opossum) OR TITLE-ABS(opossums) OR TITLE-ABS(cyprinus) OR TITLE-ABS(salamander) OR TITLE-ABS(salamanders) OR TITLE-ABS(felis) OR TITLE-ABS(mink) OR TITLE-ABS(minks) OR TITLE-ABS(swan) OR TITLE-ABS(swans) OR TITLE-ABS(norvegicus) OR TITLE-ABS(bufo) OR TITLE-ABS(torpedo) OR TITLE-ABS(bass) OR TITLE-ABS(lamprey) OR TITLE-ABS(lampreys) OR TITLE-ABS(sus) OR TITLE-ABS(python) OR TITLE-ABS(pythons) OR TITLE-ABS(tetrapod) OR TITLE-ABS(tetrapods) OR TITLE-ABS(shrew) OR TITLE-ABS(shrews) OR TITLE-ABS(lion) OR TITLE-ABS(lions) OR TITLE-ABS(hog) OR TITLE-ABS(hogs) OR TITLE-ABS(songbird) OR TITLE-ABS(songbirds) OR TITLE-ABS(oreochromis) OR TITLE-ABS(starling) OR TITLE-ABS(starlings) OR TITLE-ABS(caprine) OR TITLE-ABS(carassius) OR TITLE-ABS(owl) OR TITLE-ABS(owls) OR TITLE-ABS(newt) OR TITLE-ABS(newts) OR TITLE-ABS(papio) OR TITLE-ABS(scrofa) OR TITLE-ABS(hare) OR TITLE-ABS(hares) OR TITLE-ABS(gorilla) OR TITLE-ABS(gorillas) OR TITLE-ABS(flounder) OR TITLE-ABS(flounders) OR TITLE-ABS(goose) OR TITLE-ABS(herring) OR TITLE-ABS(herrings) OR TITLE-ABS(therian) OR TITLE-ABS(buffaloes) OR TITLE-ABS(canary) OR TITLE-ABS(sparrow) OR TITLE-ABS(sparrows) OR TITLE-ABS(microtus) OR TITLE-ABS(octopus) OR TITLE-ABS(troglodytes) OR TITLE-ABS(tuna) OR TITLE-ABS(amphibia) OR TITLE-ABS(chinchilla) OR TITLE-ABS(chinchillas) OR TITLE-ABS(ide) OR TITLE-ABS(oryzias) OR TITLE-ABS(cervus) OR TITLE-ABS(kangaroo) OR TITLE-ABS(kangaroos) OR TITLE-ABS(armadillo) OR TITLE-ABS(armadillos) OR TITLE-ABS(callithrix) OR TITLE-ABS("pan troglodytes") OR TITLE-ABS(saimiri) OR TITLE-ABS(cichlid) OR TITLE-ABS(cichlids) OR TITLE-ABS(donkey) OR TITLE-ABS(donkeys) OR TITLE-ABS(bream) OR TITLE-ABS(char) OR TITLE-

		ABS(chars) OR TITLE-ABS(finch) OR TITLE-ABS(raccoon) OR TITLE-ABS(raccoons) OR TITLE-ABS(bothrops) OR TITLE-ABS(anguilla) OR TITLE-ABS(perch) OR TITLE-ABS(cricetus) OR TITLE-ABS(seabird) OR TITLE-ABS(seabirds) OR TITLE-ABS(buck) OR TITLE-ABS(bucks) OR TITLE-ABS(naja) OR TITLE-ABS(coturnix) OR TITLE-ABS(salmonids) OR TITLE-ABS(geese) OR TITLE-ABS(minnow) OR TITLE-ABS(minnows) OR TITLE-ABS(raptor) OR TITLE-ABS(raptors) OR TITLE-ABS(merione) OR TITLE-ABS(meriones) OR TITLE-ABS(rodentia) OR TITLE-ABS(elaphus) OR TITLE-ABS(amniote) OR TITLE-ABS(amniotes) OR TITLE-ABS(elasmobranch) OR TITLE-ABS(emu) OR TITLE-ABS(emus) OR TITLE-ABS(peromyscus) OR TITLE-ABS(hominid) OR TITLE-ABS(hominids) OR TITLE-ABS(bubalus) OR TITLE-ABS(crotalus) OR TITLE-ABS(gull) OR TITLE-ABS(gulls) OR TITLE-ABS(anas) OR TITLE-ABS(anura) OR TITLE-ABS(lemur) OR TITLE-ABS(lemurs) OR TITLE-ABS(crow) OR TITLE-ABS(crows) OR TITLE-ABS(camelus) OR TITLE-ABS(gibbon) OR TITLE-ABS(gibbons) OR TITLE-ABS(waterfowl) OR TITLE-ABS(parrot) OR TITLE-ABS(parrots) OR TITLE-ABS(eels) OR TITLE-ABS(cob) OR TITLE-ABS(stickleback) OR TITLE-ABS(sticklebacks) OR TITLE-ABS(columba) OR TITLE-ABS(mesocricetus) OR TITLE-ABS(ambystoma) OR TITLE-ABS(raven) OR TITLE-ABS(ravens) OR TITLE-ABS(gadus) OR TITLE-ABS(penguin) OR TITLE-ABS(penguins) OR TITLE-ABS(orangutan) OR TITLE-ABS(orangutans) OR TITLE-ABS(sturgeon) OR TITLE-ABS(sturgeons) OR TITLE-ABS(cuniculus) OR TITLE-ABS(aves) OR TITLE-ABS(virginianus) OR TITLE-ABS(cephalopod) OR TITLE-ABS(cephalopods) OR TITLE-ABS(cebus) OR TITLE-ABS(sparus) OR TITLE-ABS(tortoise) OR TITLE-ABS(tortoises) OR TITLE-ABS(guttata) OR TITLE-ABS(morhua) OR TITLE-ABS(unguiculatus) OR TITLE-ABS(dogfish) OR TITLE-ABS(vulpes) OR TITLE-ABS(mallard) OR TITLE-ABS(mallards) OR TITLE-ABS(apodemus) OR TITLE-ABS(alligator) OR TITLE-ABS(alligators) OR TITLE-ABS(oryctolagus) OR TITLE-ABS(llama) OR TITLE-ABS(llamas) OR TITLE-ABS(reindeer) OR TITLE-ABS(mustela) OR TITLE-ABS(duckling) OR TITLE-ABS(ducklings) OR TITLE-ABS(wolves) OR TITLE-ABS(sander) OR TITLE-ABS(amazona) OR TITLE-ABS(zebu) OR TITLE-ABS(badger) OR TITLE-ABS(badgers) OR TITLE-ABS(dove) OR TITLE-ABS(doves) OR TITLE-ABS(ictalurus) OR TITLE-ABS(capra) OR TITLE-ABS(capras) OR TITLE-ABS(equus) OR TITLE-ABS(camelid) OR TITLE-ABS(camelids) OR TITLE-ABS(poecilia) OR TITLE-ABS(mule) OR TITLE-ABS(mules) OR TITLE-ABS(perciformes) OR TITLE-ABS(salvelinus) OR TITLE-ABS(labrax) OR TITLE-ABS(cyprinidae) OR TITLE-ABS(ariidae) OR TITLE-ABS(crocodile) OR TITLE-ABS(crocodiles) OR TITLE-ABS(fundulus) OR TITLE-ABS(dicentrarchus) OR TITLE-ABS(clarias) OR TITLE-ABS(cercopithecus) OR TITLE-ABS(chiroptera) OR TITLE-ABS(alpaca) OR TITLE-ABS(alpacas) OR TITLE-ABS(pike) OR TITLE-ABS(pikes) OR TITLE-ABS(paralichthys) OR TITLE-ABS(puma) OR TITLE-ABS(pumas) OR TITLE-ABS(didelphis) OR TITLE-ABS(pisces) OR TITLE-ABS(macropus) OR TITLE-ABS(triturus) OR TITLE-ABS(bison) OR TITLE-ABS(bisons) OR TITLE-ABS(epinephelus) OR TITLE-ABS(gasterosteus) OR TITLE-ABS(panthera) OR TITLE-ABS(acipenser) OR TITLE-ABS(mackerel) OR TITLE-ABS(mackerels) OR TITLE-ABS(tamarin) OR TITLE-ABS(tamarins) OR TITLE-ABS(ostrich) OR TITLE-ABS(anolis) OR TITLE-ABS(vervet) OR TITLE-ABS(vervets) OR TITLE-ABS(wallaby) OR TITLE-ABS(glareolus) OR TITLE-ABS(beaver) OR TITLE-ABS(beavers) OR TITLE-ABS(dromedary) OR TITLE-ABS(catus) OR TITLE-ABS(killifish) OR TITLE-ABS(pimephales) OR TITLE-ABS(promelas) OR TITLE-ABS(aotus) OR TITLE-ABS(phoca) OR TITLE-ABS(panda) OR TITLE-ABS(pandas) OR TITLE-ABS(porpoise) OR TITLE-ABS(porpoises) OR TITLE-ABS(myotis) OR TITLE-ABS(yak) OR TITLE-ABS(yaks) OR TITLE-ABS(agkistrodon) OR TITLE-ABS(vipera) OR TITLE-ABS(otter) OR TITLE-ABS(otters) OR TITLE-ABS(turbot) OR TITLE-ABS(turbots) OR TITLE-ABS(squamate) OR TITLE-ABS(carnivora) OR TITLE-ABS(mullet) OR TITLE-ABS(mullets) OR TITLE-ABS(hawk) OR TITLE-ABS(hawks) OR TITLE-ABS(taeniopygia) OR TITLE-ABS(seahorse) OR TITLE-ABS(seahorses) OR TITLE-ABS("poecilia reticulata") OR TITLE-ABS(falcon) OR TITLE-ABS(falcons) OR TITLE-ABS(prosimian) OR TITLE-ABS(prosimians) OR TITLE-ABS(parus) OR TITLE-ABS(perca) OR TITLE-ABS(fingerling) OR TITLE-ABS(fingerlings) OR TITLE-ABS(antelope) OR TITLE-ABS(antelopes) OR TITLE-ABS(tupaia) OR TITLE-ABS(passeriformes) OR TITLE-ABS(sepia) OR TITLE-ABS(saguinus) OR TITLE-ABS(coyote) OR TITLE-ABS(coyotes) OR TITLE-ABS(pongo) OR TITLE-ABS(meleagris) OR TITLE-ABS(reptilia) OR TITLE-ABS(lepus) OR TITLE-ABS(psittacine) OR TITLE-ABS(hagfish) OR TITLE-ABS(warbler) OR TITLE-ABS(warblers) OR TITLE-ABS("russell's viper") OR TITLE-ABS("russell's vipers") OR TITLE-ABS(smolt) OR TITLE-ABS(smolts) OR TITLE-ABS(budgerigar) OR TITLE-ABS(sardine) OR TITLE-ABS(sardines) OR TITLE-ABS(cavia) OR TITLE-ABS(cavias) OR TITLE-ABS(hyla) OR TITLE-ABS(pleurodeles) OR TITLE-ABS(siluriformes) OR TITLE-ABS("great tit") OR TITLE-ABS("great tits") OR TITLE-ABS(guppy) OR TITLE-ABS(bonobo) OR TITLE-ABS(bonobos) OR TITLE-ABS(rutilus) OR TITLE-ABS(trichosurus) OR TITLE-ABS(muridae) OR TITLE-ABS(phodopus) OR TITLE-ABS(channa) OR TITLE-ABS(squalus) OR TITLE-ABS(lynx) OR TITLE-ABS(sturnus) OR TITLE-ABS(petromyzon) OR TITLE-ABS(vitulina) OR TITLE-ABS(monodelphis) OR TITLE-ABS(cuttlefish) OR TITLE-ABS(adder) OR TITLE-ABS(adders) OR TITLE-ABS(lepomis) OR TITLE-ABS(canaria) OR TITLE-ABS(gambusia) OR TITLE-ABS(guppies) OR TITLE-ABS(xiphophorus) OR TITLE-ABS(flatfish) OR TITLE-ABS(koala) OR TITLE-ABS(koalas) OR TITLE-ABS(labeo) OR TITLE-ABS(stingray) OR TITLE-ABS(stingrays) OR TITLE-ABS(chelonia) OR TITLE-ABS(lampetra) OR TITLE-ABS(spermophilus) OR TITLE-ABS(crocodilian) OR TITLE-ABS("passer domesticus") OR TITLE-ABS(sciurus) OR TITLE-ABS(artiodactyla) OR TITLE-ABS(ranidae) OR TITLE-ABS(corvus) OR TITLE-ABS(necturus) OR TITLE-ABS(platypus) OR TITLE-ABS(canaries) OR TITLE-ABS(bovid) OR TITLE-ABS(lagopus) OR TITLE-ABS(trimeresurus) OR TITLE-ABS(gariepinus) OR TITLE-ABS(marten) OR TITLE-ABS(martens) OR TITLE-ABS(drosophilidae) OR TITLE-ABS(mugil) OR TITLE-ABS(sunfish) OR TITLE-ABS(porcellus) OR TITLE-ABS(cypriniformes) OR TITLE-ABS(alouatta) OR TITLE-ABS(scophthalmus) OR TITLE-ABS(anser) OR TITLE-ABS(electrophorus) OR TITLE-ABS(putorius) OR TITLE-ABS(iguana) OR TITLE-ABS(iguanas) OR TITLE-ABS(lama) OR TITLE-ABS(lamas) OR TITLE-ABS(takifugu) OR TITLE-ABS(circus) OR TITLE-ABS(eptesicus) OR TITLE-ABS(flycatcher) OR TITLE-ABS(galago) OR TITLE-ABS(galagos) OR TITLE-ABS(trachemys) OR TITLE-ABS(lungfish) OR TITLE-ABS(characiformes) OR TITLE-ABS(shorebird) OR TITLE-ABS(shorebirds) OR TITLE-ABS(giraffe) OR TITLE-ABS(giraffes) OR TITLE-ABS(micropterus) OR TITLE-ABS(scyliorhinus) OR TITLE-ABS(cichlidae) OR TITLE-ABS(loligo) OR TITLE-ABS(porcupine) OR TITLE-ABS(porcupines) OR TITLE-ABS(chub) OR TITLE-ABS(chubs) OR TITLE-ABS(solea) OR TITLE-ABS(pleuronectes) OR TITLE-ABS(hylidae) OR TITLE-ABS(viperidae) OR TITLE-ABS(echis) OR TITLE-ABS(sorex) OR TITLE-ABS(anchovy) OR TITLE-ABS(lagomorph) OR TITLE-ABS(ostriches) OR TITLE-ABS(vulture) OR TITLE-ABS(vultures) OR TITLE-ABS(whitefish) OR TITLE-ABS(araneus) OR TITLE-ABS(jird) OR TITLE-ABS(jirds) OR TITLE-ABS(tern) OR TITLE-ABS(esox) OR TITLE-ABS(drake) OR TITLE-ABS(drakes) OR TITLE-ABS(elapidae) OR TITLE-ABS(gallopavo) OR TITLE-ABS(chordata) OR TITLE-ABS(myodes) OR TITLE-ABS(caretta) OR TITLE-ABS(serinus) OR TITLE-ABS(grouse) OR TITLE-ABS(misgurnus) OR TITLE-ABS(meles) OR TITLE-ABS(blackbird) OR TITLE-ABS(blackbirds) OR TITLE-ABS(coregonus) OR TITLE-ABS(bobwhite) OR TITLE-ABS(bobwhites) OR TITLE-ABS(heteropneustes) OR TITLE-ABS(mammoth) OR TITLE-ABS(mammoths) OR TITLE-ABS(turdus) OR TITLE-ABS(rhinella) OR TITLE-ABS(ateles) OR TITLE-ABS(characidae) OR TITLE-ABS(clupea) OR TITLE-ABS(bungarus) OR TITLE-ABS(brill) OR TITLE-ABS("struthio camelus") OR TITLE-ABS(sloth) OR TITLE-ABS(sloths) OR TITLE-ABS(pteropus) OR TITLE-ABS(sculpin) OR TITLE-ABS(anthropoids) OR TITLE-ABS(pollock) OR TITLE-ABS(pollocks) OR TITLE-ABS(morone) OR TITLE-ABS("pan paniscus") OR TITLE-ABS(litoria) OR TITLE-ABS(chipmunk) OR TITLE-ABS(chipmunks) OR TITLE-ABS(balaenoptera) OR TITLE-ABS(marmota) OR TITLE-ABS(melopsittacus) OR TITLE-ABS(hyrax) OR TITLE-ABS(lemming) OR TITLE-ABS(lemmings) OR TITLE-ABS(halibut) OR TITLE-ABS(hylobates) OR TITLE-ABS(lates) OR TITLE-ABS(caiman) OR TITLE-ABS(caimans) OR TITLE-ABS(sigmodon) OR TITLE-ABS(stenella) OR TITLE-ABS(barbel) OR TITLE-ABS(barbels) OR TITLE-ABS(sterna) OR TITLE-ABS(parakeet) OR TITLE-ABS(parakeets) OR TITLE-ABS(phocoena) OR TITLE-ABS(leptodactylus) OR TITLE-ABS(canidae) OR TITLE-ABS(buteo) OR TITLE-ABS(harengus) OR TITLE-ABS(gopher) OR TITLE-ABS(gophers) OR TITLE-ABS(marmot) OR TITLE-ABS(marmots) OR TITLE-ABS(gosling) OR TITLE-ABS(goslings) OR TITLE-ABS(platichthys) OR TITLE-ABS(gar) OR TITLE-ABS(gars) OR TITLE-ABS(sebastes) OR TITLE-ABS(marsupialia) OR TITLE-ABS(notophthalmus) OR TITLE-ABS(gazelle) OR TITLE-ABS(gazelles) OR TITLE-ABS(insectivora) OR TITLE-ABS(paridae) OR TITLE-ABS(felidae) OR TITLE-ABS(russula) OR TITLE-ABS(galliformes) OR TITLE-

		ABS(bombina) OR TITLE-ABS(colobus) OR TITLE-ABS(echidna) OR TITLE-ABS(echidnas) OR TITLE-ABS(seabass) OR TITLE-ABS(syncerus) OR TITLE-ABS(plaice) OR TITLE-ABS("blue tit") OR TITLE-ABS("blue tits") OR TITLE-ABS(pagrus) OR TITLE-ABS(catfishes) OR TITLE-ABS(cetacea) OR TITLE-ABS(barbus) OR TITLE-ABS(cygnus) OR TITLE-ABS(ficedula) OR TITLE-ABS(chamois) OR TITLE-ABS(colubridae) OR TITLE-ABS(perches) OR TITLE-ABS(coelacanth) OR TITLE-ABS(fitch) OR TITLE-ABS(urodela) OR TITLE-ABS(cynops) OR TITLE-ABS(martes) OR TITLE-ABS(halichoerus) OR TITLE-ABS(aix) OR TITLE-ABS(salmonidae) OR TITLE-ABS(leuciscus) OR TITLE-ABS(magpie) OR TITLE-ABS(magpies) OR TITLE-ABS(silurus) OR TITLE-ABS(whiting) OR TITLE-ABS(whitings) OR TITLE-ABS(anseriformes) OR TITLE-ABS(colinus) OR TITLE-ABS(rhea) OR TITLE-ABS(chlorocebus) OR TITLE-ABS(octodon) OR TITLE-ABS(acinonyx) OR TITLE-ABS(mouflon) OR TITLE-ABS(mouflons) OR TITLE-ABS(ibex) OR TITLE-ABS(tetraodon) OR TITLE-ABS(bufonidae) OR TITLE-ABS(equidae) OR TITLE-ABS(jackal) OR TITLE-ABS(cephalopoda) OR TITLE-ABS(dendroaspis) OR TITLE-ABS(glama) OR TITLE-ABS(muskrat) OR TITLE-ABS(muskrats) OR TITLE-ABS(sable) OR TITLE-ABS(sables) OR TITLE-ABS(wildebeest) OR TITLE-ABS(streptopelia) OR TITLE-ABS(albifrons) OR TITLE-ABS(vespertilionidae) OR TITLE-ABS(woodpecker) OR TITLE-ABS(woodpeckers) OR TITLE-ABS(muntjac) OR TITLE-ABS(muntjacs) OR TITLE-ABS(archosaur) OR TITLE-ABS(branta) OR TITLE-ABS(cricetulus) OR TITLE-ABS(megalobrama) OR TITLE-ABS(poeciliidae) OR TITLE-ABS(desmodus) OR TITLE-ABS(snakehead) OR TITLE-ABS(snakeheads) OR TITLE-ABS(tench) OR TITLE-ABS(teal) OR TITLE-ABS(teals) OR TITLE-ABS(bandicoot) OR TITLE-ABS(bandicoots) OR TITLE-ABS(apteronotus) OR TITLE-ABS(phyllostomidae) OR TITLE-ABS(crocidura) OR TITLE-ABS(buzzard) OR TITLE-ABS(buzzards) OR TITLE-ABS(larimichthys) OR TITLE-ABS(cercocebus) OR TITLE-ABS(pipistrellus) OR TITLE-ABS(erithacus) OR TITLE-ABS(impala) OR TITLE-ABS(impalas) OR TITLE-ABS(rousettus) OR TITLE-ABS(haddock) OR TITLE-ABS(haddocks) OR TITLE-ABS(tinca) OR TITLE-ABS(ratite) OR TITLE-ABS(calidris) OR TITLE-ABS(cynoglossus) OR TITLE-ABS(hypophthalmichthys) OR TITLE-ABS(bullock) OR TITLE-ABS(bullocks) OR TITLE-ABS(dromedaries) OR TITLE-ABS(alectoris) OR TITLE-ABS(filly) OR TITLE-ABS(salamandra) OR TITLE-ABS(cingulata) OR TITLE-ABS(bitis) OR TITLE-ABS(grus) OR TITLE-ABS(ammodytes) OR TITLE-ABS(macaw) OR TITLE-ABS(macaws) OR TITLE-ABS(hypoleuca) OR TITLE-ABS(sapajus) OR TITLE-ABS(cyprinodontiformes) OR TITLE-ABS(hippopotamus) OR TITLE-ABS(pelophylax) OR TITLE-ABS(capybara) OR TITLE-ABS(capybaras) OR TITLE-ABS(weasel) OR TITLE-ABS(weasels) OR TITLE-ABS(cairina) OR TITLE-ABS(cynomys) OR TITLE-ABS(lutra) OR TITLE-ABS(cockatoo) OR TITLE-ABS(cockatoos) OR TITLE-ABS(lachesis) OR TITLE-ABS(lagomorpha) OR TITLE-ABS(rupicapra) OR TITLE-ABS(daboia) OR TITLE-ABS("orang utan") OR TITLE-ABS("orang utans") OR TITLE-ABS(platyrrhini) OR TITLE-ABS(charadriiformes) OR TITLE-ABS(micrurus) OR TITLE-ABS(psittaciformes) OR TITLE-ABS(spalax) OR TITLE-ABS(loris) OR TITLE-ABS(mustelidae) OR TITLE-ABS(sylvilagus) OR TITLE-ABS(vitticeps) OR TITLE-ABS(cockatiel) OR TITLE-ABS(mustelus) OR TITLE-ABS(cottus) OR TITLE-ABS(erythrocebus) OR TITLE-ABS(dipodomys) OR TITLE-ABS(platessa) OR TITLE-ABS(callicebus) OR TITLE-ABS(loricariidae) OR TITLE-ABS(catostomus) OR TITLE-ABS(cuneata) OR TITLE-ABS(cyanistes) OR TITLE-ABS(cyprinodon) OR TITLE-ABS(sigmodontinae) OR TITLE-ABS(elasmobranchii) OR TITLE-ABS(trichechus) OR TITLE-ABS(sauropsid) OR TITLE-ABS(xenarthra) OR TITLE-ABS(dormouse) OR TITLE-ABS(perissodactyla) OR TITLE-ABS(nautilus) OR TITLE-ABS(cirrhinus) OR TITLE-ABS(gulo) OR TITLE-ABS(tragelaphus) OR TITLE-ABS(merula) OR TITLE-ABS(numida) OR TITLE-ABS(sciaenidae) OR TITLE-ABS(cerastes) OR TITLE-ABS(sciuridae) OR TITLE-ABS(gibbosus) OR TITLE-ABS(octopuses) OR TITLE-ABS(eland) OR TITLE-ABS(elands) OR TITLE-ABS(phyllomedusa) OR TITLE-ABS(pogona) OR TITLE-ABS(walrus) OR TITLE-ABS(agamidae) OR TITLE-ABS(leptodactylidae) OR TITLE-ABS(ridibundus) OR TITLE-ABS(leontopithecus) OR TITLE-ABS(anteater) OR TITLE-ABS(anteaters) OR TITLE-ABS(pelodiscus) OR TITLE-ABS(cebidae) OR TITLE-ABS(columbianus) OR TITLE-ABS("pelteobagrus fulvidraco") OR TITLE-ABS(hominoidea) OR TITLE-ABS(mandrillus) OR TITLE-ABS("zonotrichia leucophrys") OR TITLE-ABS(agama) OR TITLE-ABS(gobiocypris) OR TITLE-ABS("bearded dragon") OR TITLE-ABS("bearded dragons") OR TITLE-ABS(sarotherodon) OR TITLE-ABS(talpa) OR TITLE-ABS(discoglossus) OR TITLE-ABS(hagfishes) OR TITLE-ABS(sphenodon) OR TITLE-ABS(gudgeon) OR TITLE-ABS(amphiuma) OR TITLE-ABS(aythya) OR TITLE-ABS(tenrec) OR TITLE-ABS(tenrec) OR TITLE-ABS(hominidae) OR TITLE-ABS(risoria) OR TITLE-ABS(salamandridae) OR TITLE-ABS(camelidae) OR TITLE-ABS(columbiformes) OR TITLE-ABS(latimeria) OR TITLE-ABS(plover) OR TITLE-ABS(plovers) OR TITLE-ABS(afrotheria) OR TITLE-ABS("falco sparverius") OR TITLE-ABS(polecat) OR TITLE-ABS(polecats) OR TITLE-ABS(crotalinae) OR TITLE-ABS(salvadora) OR TITLE-ABS(tarsier) OR TITLE-ABS(lucioperca) OR TITLE-ABS(anchovies) OR TITLE-ABS(lungfishes) OR TITLE-ABS(terrapin) OR TITLE-ABS("dromaius novaehollandiae") OR TITLE-ABS(lateolabrax) OR TITLE-ABS(eigenmannia) OR TITLE-ABS(pelamis) OR TITLE-ABS(theropithecus) OR TITLE-ABS(murinae) OR TITLE-ABS(gander) OR TITLE-ABS(gymnotus) OR TITLE-ABS(pseudacris) OR TITLE-ABS(gymnophiona) OR TITLE-ABS(gymnotiformes) OR TITLE-ABS(laticauda) OR TITLE-ABS(falconiformes) OR TITLE-ABS(dugong) OR TITLE-ABS(dugongs) OR TITLE-ABS(pintail) OR TITLE-ABS(pintails) OR TITLE-ABS(rook) OR TITLE-ABS(rooks) OR TITLE-ABS(lasiurus) OR TITLE-ABS(catshark) OR TITLE-ABS(catsharks) OR TITLE-ABS(micropogonias) OR TITLE-ABS("red junglefowl") OR TITLE-ABS(paddlefish) OR TITLE-ABS(ophiophagus) OR TITLE-ABS(hollandicus) OR TITLE-ABS(nymphicus) OR TITLE-ABS(pimelodidae) OR TITLE-ABS(aepyceros) OR TITLE-ABS(cobitidae) OR TITLE-ABS(strigiformes) OR TITLE-ABS(cobitis) OR TITLE-ABS(dormice) OR TITLE-ABS(alytes) OR TITLE-ABS(calloselasma) OR TITLE-ABS(guanaco) OR TITLE-ABS(phasianidae) OR TITLE-ABS("round goby") OR TITLE-ABS(trichogaster) OR TITLE-ABS(catarrhini) OR TITLE-ABS(eelpout) OR TITLE-ABS(eelpouts) OR TITLE-ABS(galaxias) OR TITLE-ABS(gaur) OR TITLE-ABS(pungitius) OR TITLE-ABS(suslik) OR TITLE-ABS(susliks) OR TITLE-ABS(flatfishes) OR TITLE-ABS(percidae) OR TITLE-ABS(caprinae) OR TITLE-ABS(todarodes) OR TITLE-ABS(osmerus) OR TITLE-ABS(ameiurus) OR TITLE-ABS(anthropoidea) OR TITLE-ABS("castor canadensis") OR TITLE-ABS(pouting) OR TITLE-ABS(poutings) OR TITLE-ABS(tetraodontiformes) OR TITLE-ABS(arvicolinae) OR TITLE-ABS(siamang) OR TITLE-ABS(siamangs) OR TITLE-ABS("castor fiber") OR TITLE-ABS(nomascus) OR TITLE-ABS("red knot") OR TITLE-ABS("red knots") OR TITLE-ABS(syngnathidae) OR TITLE-ABS(iguanidae) OR TITLE-ABS(eretmochelys) OR TITLE-ABS(ursidae) OR TITLE-ABS(callimico) OR TITLE-ABS(columbidae) OR TITLE-ABS(microhylidae) OR TITLE-ABS(anaxyrus) OR TITLE-ABS(menidia) OR TITLE-ABS(pipistrelle) OR TITLE-ABS(greylag) OR TITLE-ABS(pipidae) OR TITLE-ABS(scandentia) OR TITLE-ABS(bowfin) OR TITLE-ABS(bowfins) OR TITLE-ABS(dendrobatidae) OR TITLE-ABS(zenaida) OR TITLE-ABS(bushbaby) OR TITLE-ABS(harrier) OR TITLE-ABS(harriers) OR TITLE-ABS(macropodidae) OR TITLE-ABS(pygerythrus) OR TITLE-ABS(clupeidae) OR TITLE-ABS(odorrana) OR TITLE-ABS(corvidae) OR TITLE-ABS(jerboa) OR TITLE-ABS(jerboas) OR TITLE-ABS(canutus) OR TITLE-ABS(hylobatidae) OR TITLE-ABS(clupeiformes) OR TITLE-ABS("great cormorant") OR TITLE-ABS("great cormorants") OR TITLE-ABS(scorpaeniformes) OR TITLE-ABS(chondrostean) OR TITLE-ABS(garfish) OR TITLE-ABS(proboscidea) OR TITLE-ABS(psetta) OR TITLE-ABS(diapsid) OR TITLE-ABS(serotinus) OR TITLE-ABS(tetrao) OR TITLE-ABS(walruses) OR TITLE-ABS(carcharhiniformes) OR TITLE-ABS(leucoraja) OR TITLE-ABS(pumpkinseed) OR TITLE-ABS(dosidicus) OR TITLE-ABS(acipenseriformes) OR TITLE-ABS(daubentonii) OR TITLE-ABS(emberizidae) OR TITLE-ABS(gadiformes) OR TITLE-ABS(hyraxes) OR TITLE-ABS(stizostedion) OR TITLE-ABS(wolverine) OR TITLE-ABS(wolverines) OR TITLE-ABS(lissotriton) OR TITLE-ABS(acanthurus) OR TITLE-ABS(centrarchidae) OR TITLE-ABS(gloydius) OR TITLE-ABS(laurasiatheria) OR TITLE-ABS(limosa) OR TITLE-ABS(psittacula) OR TITLE-ABS(leporidae) OR TITLE-ABS(proteidae) OR TITLE-ABS(zander) OR TITLE-ABS(zanders) OR TITLE-ABS(arapaima) OR TITLE-ABS(bagridae) OR TITLE-ABS(cyprinodontidae) OR TITLE-ABS(mithun) OR TITLE-ABS(pandion) OR TITLE-ABS(jackdaw) OR TITLE-ABS(jackdaws) OR TITLE-ABS(procyonidae) OR TITLE-ABS(carus) OR TITLE-ABS(jaculus) OR TITLE-ABS(salmoniformes) OR TITLE-ABS("common sole") OR TITLE-ABS("common soles") OR TITLE-ABS(protobothrops) OR TITLE-ABS(calamita) OR TITLE-ABS(brachyteles) OR TITLE-ABS(trionyx) OR TITLE-ABS(turdidae) OR TITLE-ABS(boidae) OR TITLE-ABS(luscinia) OR TITLE-ABS(pugnax) OR TITLE-ABS(euarchontoglires) OR TITLE-ABS(saithe) OR TITLE-ABS(saithes) OR TITLE-ABS(symphalangus) OR TITLE-ABS(aardvark) OR

		TITLE-ABS(aardvarks) OR TITLE-ABS(oystercatcher) OR TITLE-ABS(oystercatchers) OR TITLE-ABS(arius) OR TITLE-ABS(corydoras) OR TITLE-ABS(poacher) OR TITLE-ABS(poachers) OR TITLE-ABS(aurochs) OR TITLE-ABS(cebuella) OR TITLE-ABS(crecca) OR TITLE-ABS(lemuridae) OR TITLE-ABS(sirenia) OR TITLE-ABS(lemmus) OR TITLE-ABS(perdix) OR TITLE-ABS(glires) OR TITLE-ABS(lepidosaur) OR TITLE-ABS(muskox) OR TITLE-ABS(deinagkistrodon) OR TITLE-ABS(pholidota) OR TITLE-ABS(holocephali) OR TITLE-ABS(cercopithecinae) OR TITLE-ABS(clariidae) OR TITLE-ABS(agapornis) OR TITLE-ABS(doryteuthis) OR TITLE-ABS(tyrannidae) OR TITLE-ABS(dicroglossidae) OR TITLE-ABS(godwit) OR TITLE-ABS(godwits) OR TITLE-ABS(monedula) OR TITLE-ABS(pongidae) OR TITLE-ABS(atheriniformes) OR TITLE-ABS(colobinae) OR TITLE-ABS(lophocebus) OR TITLE-ABS(atelidae) OR TITLE-ABS(cottidae) OR TITLE-ABS(leucopsis) OR TITLE-ABS(acanthuridae) OR TITLE-ABS(didelphimorphia) OR TITLE-ABS(elver) OR TITLE-ABS(elvers) OR TITLE-ABS(lapponica) OR TITLE-ABS(dermoptera) OR TITLE-ABS("european hake") OR TITLE-ABS("european hakes") OR TITLE-ABS(gerbillinae) OR TITLE-ABS(banteng) OR TITLE-ABS(hartebeest) OR TITLE-ABS(hartebeests) OR TITLE-ABS(hogget) OR TITLE-ABS(haematopus) OR TITLE-ABS("anguis fragilis") OR TITLE-ABS("grey heron") OR TITLE-ABS("grey herons") OR TITLE-ABS("blue whiting") OR TITLE-ABS("blue whitings") OR TITLE-ABS(furnariidae) OR TITLE-ABS(macrovipera) OR TITLE-ABS(esocidae) OR TITLE-ABS(lapwing) OR TITLE-ABS(lapwings) OR TITLE-ABS(mylopharyngodon) OR TITLE-ABS(wallabia) OR TITLE-ABS(beloniformes) OR TITLE-ABS(potoroo) OR TITLE-ABS(potoroos) OR TITLE-ABS("athene noctua") OR TITLE-ABS(pleuronectidae) OR TITLE-ABS(bushbabies) OR TITLE-ABS(muscicapidae) OR TITLE-ABS(alligatoridae) OR TITLE-ABS(fuligula) OR TITLE-ABS("bush baby") OR TITLE-ABS(guineafowl) OR TITLE-ABS(spoonbill) OR TITLE-ABS(spoonbills) OR TITLE-ABS(viverridae) OR TITLE-ABS(catostomidae) OR TITLE-ABS(zebrafishes) OR TITLE-ABS(ibexes) OR TITLE-ABS(vendace) OR TITLE-ABS(estrildidae) OR TITLE-ABS(monotremata) OR TITLE-ABS(sepiella) OR TITLE-ABS(ambystomatidae) OR TITLE-ABS(shelduck) OR TITLE-ABS(shelducks) OR TITLE-ABS(treeshrew) OR TITLE-ABS(treeshrews) OR TITLE-ABS(hoplobatrachus) OR TITLE-ABS(pochard) OR TITLE-ABS(hoolock) OR TITLE-ABS(hoolocks) OR TITLE-ABS(lynxes) OR TITLE-ABS(antilope) OR TITLE-ABS(antilopes) OR TITLE-ABS(blackbuck) OR TITLE-ABS(blackbucks) OR TITLE-ABS(cricetinae) OR TITLE-ABS(paramisgurnus) OR TITLE-ABS(skylark) OR TITLE-ABS(skylarks) OR TITLE-ABS(soleidae) OR TITLE-ABS(allobates) OR TITLE-ABS("northern wheatear") OR TITLE-ABS("northern wheatears") OR TITLE-ABS(pitheciidae) OR TITLE-ABS(takin) OR TITLE-ABS(theria) OR TITLE-ABS(vanellus) OR TITLE-ABS(galaxiidae) OR TITLE-ABS(lorisidae) OR TITLE-ABS(ostralegus) OR TITLE-ABS(palaeognathae) OR TITLE-ABS("stone loach") OR TITLE-ABS(alauda) OR TITLE-ABS(callitrichinae) OR TITLE-ABS(caniformia) OR TITLE-ABS(duttaphrynus) OR TITLE-ABS(ictaluridae) OR TITLE-ABS(osteoglossiformes) OR TITLE-ABS(poultries) OR TITLE-ABS(curema) OR TITLE-ABS("ruddy turnstone") OR TITLE-ABS("ruddy turnstones") OR TITLE-ABS(sheatfish) OR TITLE-ABS(sunfishes) OR TITLE-ABS(centropomidae) OR TITLE-ABS(hemachatus) OR TITLE-ABS(platalea) OR TITLE-ABS(thamnophilidae) OR TITLE-ABS("song thrush") OR TITLE-ABS(atherinopsidae) OR TITLE-ABS(siluridae) OR TITLE-ABS(tadorna) OR TITLE-ABS(chroicocephalus) OR TITLE-ABS(ermine) OR TITLE-ABS(ermines) OR TITLE-ABS(gavialis) OR TITLE-ABS(ruff) OR TITLE-ABS(tupaiidae) OR TITLE-ABS(diprotodontia) OR TITLE-ABS(hyaenidae) OR TITLE-ABS(antilopinae) OR TITLE-ABS(crocodylidae) OR TITLE-ABS(herpestidae) OR TITLE-ABS(hippopotamidae) OR TITLE-ABS("northern shoveler") OR TITLE-ABS("round gobies") OR TITLE-ABS(cheirogaleidae) OR TITLE-ABS(indriidae) OR TITLE-ABS(fundulidae) OR TITLE-ABS(pythonidae) OR TITLE-ABS(rhynchocephalia) OR TITLE-ABS(anodorhynchus) OR TITLE-ABS("red-backed shrike") OR TITLE-ABS("red-backed shrikes") OR TITLE-ABS(triakidae) OR TITLE-ABS(phalangeridae) OR TITLE-ABS(aoudad) OR TITLE-ABS(boreoeutheria) OR TITLE-ABS("eurasian jay") OR TITLE-ABS("eurasian jays") OR TITLE-ABS(feliformia) OR TITLE-ABS(haplorhini) OR TITLE-ABS(osteoglossidae) OR TITLE-ABS(paenungulata) OR TITLE-ABS(struthioniformes) OR TITLE-ABS(ferina) OR TITLE-ABS(sanderling) OR TITLE-ABS(sanderlings) OR TITLE-ABS(spheniscidae) OR TITLE-ABS(cuttlefishes) OR TITLE-ABS(cygnet) OR TITLE-ABS(dasycneme) OR TITLE-ABS(gadwall) OR TITLE-ABS(gadwalls) OR TITLE-ABS("pelobates fuscus") OR TITLE-ABS(wryneck) OR TITLE-ABS(wrynecks) OR TITLE-ABS(afrosoricida) OR TITLE-ABS(culaea) OR TITLE-ABS("dover sole") OR TITLE-ABS("dover soles") OR TITLE-ABS(paralichthyidae) OR TITLE-ABS(passeridae) OR TITLE-ABS(osteolaemus) OR TITLE-ABS("song thrushes") OR TITLE-ABS(bluethroat) OR TITLE-ABS(bluethroats) OR TITLE-ABS(hydrophiidae) OR TITLE-ABS(megrim) OR TITLE-ABS(mephitidae) OR TITLE-ABS(strepsirhini) OR TITLE-ABS(tomistoma) OR TITLE-ABS(epidalea) OR TITLE-ABS(osmeriformes) OR TITLE-ABS("bush babies") OR TITLE-ABS(tarsiiform) OR TITLE-ABS(atelinae) OR TITLE-ABS(bufotes) OR TITLE-ABS("eurasian coot") OR TITLE-ABS("eurasian coots") OR TITLE-ABS(galagidae) OR TITLE-ABS(geopelia) OR TITLE-ABS(philomachus) OR TITLE-ABS(tubulidentata) OR TITLE-ABS(bombinatoridae) OR TITLE-ABS(pelobatidae) OR TITLE-ABS(tachysurus) OR TITLE-ABS(ailuridae) OR TITLE-ABS(woodlark) OR TITLE-ABS(woodlarks) OR TITLE-ABS(alcelaphinae) OR TITLE-ABS(redshank) OR TITLE-ABS(redshanks) OR TITLE-ABS(salientia) OR TITLE-ABS("sand smelt") OR TITLE-ABS("sand smelts") OR TITLE-ABS(woodmice) OR TITLE-ABS(woodmouse) OR TITLE-ABS(dasyproctidae) OR TITLE-ABS("eurasian wigeon") OR TITLE-ABS("eurasian wigeons") OR TITLE-ABS(garganey) OR TITLE-ABS(garganeys) OR TITLE-ABS("lemon sole") OR TITLE-ABS("lemon soles") OR TITLE-ABS("common dab") OR TITLE-ABS("common dabs") OR TITLE-ABS(graylag) OR TITLE-ABS(graylags) OR TITLE-ABS(leucorodia) OR TITLE-ABS(osphronemidae) OR TITLE-ABS(bewickii) OR TITLE-ABS("common moorhen") OR TITLE-ABS("common moorhens") OR TITLE-ABS(decapodiformes) OR TITLE-ABS(gobbler) OR TITLE-ABS(gobblers) OR TITLE-ABS(odontophoridae) OR TITLE-ABS(paddlefishes) OR TITLE-ABS(eutheria) OR TITLE-ABS(salmonine) OR TITLE-ABS(esociformes) OR TITLE-ABS("eurasian woodcock") OR TITLE-ABS("eurasian woodcocks") OR TITLE-ABS("european smelt") OR TITLE-ABS("european smelts") OR TITLE-ABS(goldfishes) OR TITLE-ABS(tenches) OR TITLE-ABS(tyranni) OR TITLE-ABS("common chaffinch") OR TITLE-ABS("common chaffinchs") OR TITLE-ABS("common redstart") OR TITLE-ABS("common redstarts") OR TITLE-ABS("common roach") OR TITLE-ABS("common roachs") OR TITLE-ABS("great knot") OR TITLE-ABS("great knots") OR TITLE-ABS(potoroidae) OR TITLE-ABS(alytidae) OR TITLE-ABS(coregonine) OR TITLE-ABS(dipteral) OR TITLE-ABS(leveret) OR TITLE-ABS("poeciliopsis gracilis") OR TITLE-ABS(amphiumidae) OR TITLE-ABS(batrachoidiformes) OR TITLE-ABS("bighead goby") OR TITLE-ABS(heteropneustidae) OR TITLE-ABS(lullula) OR TITLE-ABS("norway pout") OR TITLE-ABS("norway pouts") OR TITLE-ABS(sipunculida) OR TITLE-ABS(dogfishes) OR TITLE-ABS(sebastidae) OR TITLE-ABS(tarsiidae) OR TITLE-ABS(alethinophidia) OR TITLE-ABS("common nase") OR TITLE-ABS("common nases") OR TITLE-ABS("common sandpiper") OR TITLE-ABS("common sandpipers") OR TITLE-ABS("eurasian blackcap") OR TITLE-ABS("eurasian blackcaps") OR TITLE-ABS(pterocnemia) OR TITLE-ABS(syngnathiformes) OR TITLE-ABS("common chaffinches") OR TITLE-ABS(eupleridae) OR TITLE-ABS(octopodiformes) OR TITLE-ABS(phascolarctidae) OR TITLE-ABS(scophthalmidae) OR TITLE-ABS("starry smooth-hound") OR TITLE-ABS("starry smooth-hounds") OR TITLE-ABS(whitefishes) OR TITLE-ABS(cuniculidae) OR TITLE-ABS("european sprat") OR TITLE-ABS("european sprats") OR TITLE-ABS("rosy bitterling") OR TITLE-ABS("rosy bitterlings") OR TITLE-ABS("common dace") OR TITLE-ABS("common daces") OR TITLE-ABS("lesser weever") OR TITLE-ABS("lesser weevers") OR TITLE-ABS(scaldfish) OR TITLE-ABS("water rail") OR TITLE-ABS("water rails") OR TITLE-ABS(alouattinae) OR TITLE-ABS(centrarchiformes) OR TITLE-ABS("common whitethroat") OR TITLE-ABS("common whitethroats") OR TITLE-ABS(gavialidae) OR TITLE-ABS("grey gurnard") OR TITLE-ABS("grey gurnards") OR TITLE-ABS(lateolabracidae) OR TITLE-ABS(rheiformes) OR TITLE-ABS("tub gurnard") OR TITLE-ABS("tub gurnards") OR TITLE-ABS("common chiffchaff") OR TITLE-ABS("common chiffchaffs") OR TITLE-ABS(garfishes) OR TITLE-ABS("lesser whitethroat") OR TITLE-ABS("lesser whitethroats") OR TITLE-ABS(myoxidae) OR TITLE-ABS(seabasses) OR TITLE-ABS(spariformes) OR TITLE-ABS(umbridae) OR TITLE-ABS("yellow boxfish") OR TITLE-ABS(anabantiformes) OR TITLE-ABS(aotidae) OR TITLE-ABS("common bleak") OR TITLE-ABS("common bleaks") OR TITLE-ABS("common rudd") OR TITLE-ABS("common rudds") OR TITLE-ABS("greater pipefish") OR TITLE-

		ABS(hapale) OR TITLE-ABS(nandiniidae) OR TITLE-ABS("stone loaches") OR TITLE-ABS(whinchat) OR TITLE-ABS(whinchats) OR TITLE-ABS(acanthuriformes) OR TITLE-ABS("brotula barbata") OR TITLE-ABS("common ling") OR TITLE-ABS("common lings") OR TITLE-ABS("common roaches") OR TITLE-ABS(cottonrat) OR TITLE-ABS(cottonrats) OR TITLE-ABS(douroucoulis) OR TITLE-ABS(dromaiidae) OR TITLE-ABS(fitches) OR TITLE-ABS(fitchew) OR TITLE-ABS(galaxiiformes) OR TITLE-ABS(laprine) OR TITLE-ABS(saimiriinae) OR TITLE-ABS(solenette) OR TITLE-ABS(tarsii) OR TITLE-ABS("tompot blenny") OR TITLE-ABS("common dragonet") OR TITLE-ABS("common dragonets") OR TITLE-ABS("longspined bullhead") OR TITLE-ABS("longspined bullheads") OR TITLE-ABS(monotremate) OR TITLE-ABS(monotremates) OR TITLE-ABS(pempheriformes) OR TITLE-ABS(perdicinae) OR TITLE-ABS(presbytini) OR TITLE-ABS(smegmamorpha) OR TITLE-ABS("bighead gobies") OR TITLE-ABS("carangaria incertae sedis") OR TITLE-ABS(coiidae) OR TITLE-ABS("fivebeard rockling") OR TITLE-ABS(foulmart) OR TITLE-ABS(foumart) OR TITLE-ABS(grasskeet) OR TITLE-ABS("greater pipefishes") OR TITLE-ABS(ibices) OR TITLE-ABS(millionfish) OR TITLE-ABS(muguliformes) OR TITLE-ABS("norwegian topknot") OR TITLE-ABS(peewit) OR TITLE-ABS("red sea sailfin tang") OR TITLE-ABS(rupicapras) OR TITLE-ABS(sheatfishes) OR TITLE-ABS("tompot blennies") OR TITLE-ABS("twait shad") OR TITLE-ABS("yellow boxfishes")) AND NOT ALL(medline)))
#5	Cardiac AND repair AND ECM and animals 1,932 hits	#1 AND #2 AND #3 AND #4
#6	Remove reviews 1,679 hits	#5 AND (LIMIT-TO (DOCTYPE , "ar") OR LIMIT-TO (DOCTYPE , "cp") OR LIMIT-TO (DOCTYPE , "er"))
#7	*Retrieve reviews for separate screening* 242 hits	#5 AND (LIMIT-TO (DOCTYPE , "re") OR LIMIT-TO (DOCTYPE , "ch") OR LIMIT-TO (DOCTYPE , "bk") OR LIMIT-TO (DOCTYPE , "cr"))

Supplementary File 3 – full search strings for Pubmed 06-07-2022

Original search 06-07-2022

PubMed	06-07-2022	2236 hits without reviews
#1	Heart injury<br><br>Hits:<br>1,742,176	((“myocardial ischemia”[Mesh] OR “coronary occlusion”[Mesh] OR “heart transplantation”[Mesh] OR “heart failure”[Mesh] OR “heart”[Mesh] OR “myocardium”[Mesh] OR “cardiac fibrosis”[tiab] OR heart[tiab] OR hearts[tiab] OR cardiac[tiab] OR myocardial[tiab] OR myocardium[tiab] OR myocardia[tiab] OR myocard[tiab]) OR ((“ischemia”[Mesh:noexp] OR “infarction”[Mesh:noexp] OR “warm ischemia”[Mesh] OR “cold ischemia”[Mesh] OR “reperfusion injury”[Mesh] OR “transplantation”[Mesh:noexp] OR “graft survival”[Mesh] OR “graft rejection”[Mesh] OR “transplants”[Mesh] OR ischemia[tiab] OR ischaemia[tiab] OR ischemic[tiab] OR ischaemic[tiab] OR infarct[tiab] OR infarcts[tiab] OR infarction[tiab] OR infarctions[tiab] OR infarcted[tiab] OR “reperfusion injury”[tiab] OR “reperfusion injuries”[tiab] OR I/R[tiab] OR IRI[tiab] OR occlusion[tiab] OR failure[tiab] OR decompensation[tiab] OR insufficiency[tiab] OR incompetence[tiab] OR overload[tiab] OR transplantation[tiab] OR transplantations[tiab] OR transplant[tiab] OR transplants[tiab] OR graft[tiab] OR grafts[tiab]) AND (heart[tiab] OR hearts[tiab] OR cardiac[tiab] OR myocardial[tiab] OR myocardium[tiab] OR myocardia[tiab] OR myocard[tiab]))) NOT ("Heart Valves"[MeSH:noexp] OR "Aortic Valve"[MeSH] OR "Chordae Tendineae"[MeSH] OR "Mitral Valve"[MeSH] OR "Pulmonary Valve"[MeSH] OR "Tricuspid Valve"[MeSH] OR "Heart Valves"[tiab] OR "Heart Valve"[tiab] OR "Aortic Valves"[tiab] OR "Aortic Valve"[tiab] OR "Chordae Tendineae"[tiab] OR "Mitral Valves"[tiab] OR "Mitral Valve"[tiab] OR "Pulmonary Valves"[tiab] OR "Pulmonary Valve"[tiab] OR "Tricuspid Valves"[tiab] OR "Tricuspid Valve"[tiab] OR "Cardiac Valves"[tiab] OR "Cardiac Valve"[tiab])
#2	Repair<br><br>Hits:<br>734,933	"Regeneration"[MeSH:noexp] OR "Wound Healing"[Mesh:noexp] OR "Regenerative Medicine"[Mesh] OR “Tissue Scaffolds”[MeSH] OR “Tissue Engineering”[MeSH] OR regeneration[tiab] OR regenerations[tiab] OR regenerated[tiab] OR regenerative[tiab] OR “wound healing”[tiab] OR “wound healings”[tiab] OR renewal[tiab] OR repair[tiab] OR “Tissue Engineering”[tiab] OR “Tissue Scaffolds”[tiab] OR “Tissue Scaffold”[tiab]
#2	ECM<br><br>Hits:<br>649,182	"Extracellular Matrix"[Mesh:noexp] OR "Decellularized Extracellular Matrix"[Mesh] OR "Extracellular Matrix Proteins"[Mesh] OR “Glycosaminoglycans”[Mesh] OR “Chondroitin”[Mesh] OR “Chondroitin Sulfates”[Mesh] OR “Dermatan Sulfate”[Mesh] OR “Heparin”[Mesh] OR “Heparin, Low-Molecular-Weight ”[Mesh] OR “Heparinoids”[Mesh] OR “Heparitin Sulfate”[Mesh] OR “Heparan Sulfate Proteoglycans”[Mesh] OR “Hyaluronic Acid”[Mesh] OR “Keratan Sulfate”[Mesh] OR “Dalteparin”[Mesh] OR “Enoxaparin”[Mesh] OR “Nadroparin”[Mesh] OR “Tinzaparin”[Mesh] OR “Biocompatible Materials”[Mesh] OR “Extracellular Matrix Protein”[tiab] OR “Extracellular Matrix Proteins”[tiab] OR “Extracellular Matrix”[tiab] OR “Extracellular Matrices”[tiab] OR “ECM”[tiab] OR “ECMs”[tiab] OR “Decellularized Extracellular Matrix”[tiab] OR “Decellularized Extracellular Matrices”[tiab] OR “Decellularized ECM”[tiab] OR “Decellularized ECMs”[tiab] OR “Glycosaminoglycans”[tiab] OR “Glycosaminoglycan”[tiab] OR “Chondroitin”[tiab] OR “Chondroitin Sulfates”[tiab] OR “Chondroitin Sulfate”[tiab] OR “Dermatan Sulfates”[tiab] OR “Dermatan Sulfate”[tiab] OR “Heparin”[tiab] OR “Dalteparin”[tiab] OR “Enoxaparin”[tiab] OR “Nadroparin”[tiab] OR “Tinzaparin”[tiab] OR “Heparinoids”[tiab] OR “Heparitin Sulfate”[tiab] OR “Heparan Sulfate Proteoglycans”[tiab] OR “Hyaluronic Acid”[tiab] OR “Keratan Sulfate”[tiab] OR “Biocompatible Materials”[tiab] OR Proteoglycans[tiab] OR Proteoglycan[tiab] OR “Activated-Leukocyte Cell Adhesion Molecule”[tiab] OR “ADAMTS Proteins”[tiab] OR “ADAMTS1 Protein”[tiab] OR “ADAMTS13 Protein”[tiab] OR “ADAMTS4 Protein”[tiab] OR “ADAMTS5 Protein”[tiab] OR “ADAMTS7 Protein”[tiab] OR “ADAMTS9 Protein”[tiab] OR “Aggrecan”[tiab] OR “Aggrecans”[tiab] OR “Cartilage Oligomeric Matrix Protein”[tiab] OR “Cartilage Oligomeric Matrix Proteins”[tiab] OR “CCN Intercellular Signaling Protein”[tiab] OR “CCN Intercellular Signaling Proteins”[tiab] OR “Connective Tissue Growth Factor”[tiab] OR “Connective Tissue Growth Factors”[tiab] OR “Cysteine-Rich Protein 61”[tiab] OR “Nephroblastoma Overexpressed Protein”[tiab] OR “Collagen”[tiab] OR “Collagens”[tiab] OR “Fibrillar Collagens”[tiab] OR

		"Non-Fibrillar Collagens"[tiab] OR "Procollagen"[tiab] OR "Tropocollagen"[tiab] OR "Elastin"[tiab] OR "Tropoelastin"[tiab] OR "Fibrillins"[tiab] OR "Fibrillin-1"[tiab] OR "Fibrillin-2"[tiab] OR "Fibronectins"[tiab] OR "Integrin-Binding Sialoprotein"[tiab] OR "Laminin"[tiab] OR "Latent TGF-beta Binding Proteins"[tiab] OR "Matrilin Proteins"[tiab] OR "Netrins"[tiab] OR "Netrin-1"[tiab] OR "Osteopontin"[tiab] OR "Reelin Protein"[tiab] OR "Small Leucine-Rich Proteoglycans"[tiab] OR "Biglycan"[tiab] OR "Decorin"[tiab] OR "Fibromodulin"[tiab] OR "Lumican"[tiab] OR "Tenascin"[tiab] OR "Versicans"[tiab] OR "Vitronectin"[tiab] OR "Chondroitin Sulfate Proteoglycans"[tiab] OR "Aggrecans"[tiab] OR "Versicans"[tiab] OR "Versican"[tiab] OR "Small Leucine-Rich Proteoglycans"[tiab] OR "Biglycan"[tiab] OR "Decorin"[tiab] OR "Fibromodulin"[tiab] OR "Lumican"[tiab] OR "Hyaluronan Receptors"[tiab] OR "Hyaluronan Receptor"[tiab] OR "Glypicans"[tiab] OR "Syndecans"[tiab] OR "Syndecan"[tiab] OR "Syndecan-1"[tiab] OR "Syndecan-2"[tiab] OR "Syndecan-3"[tiab] OR "Syndecan-4"[tiab] OR "Syndecan1"[tiab] OR "Syndecan2"[tiab] OR "Syndecan3"[tiab] OR "Syndecan4"[tiab] OR "Glypican"[tiab] OR "Glypican-5"[tiab] OR "Glypican5"[tiab] OR "Glypican-3"[tiab] OR "Glypican3"[tiab] OR "Glypican-4"[tiab] OR "Glypican4"[tiab] OR "Glypican-1"[tiab] OR "Glypican1"[tiab] OR "Glypican-2"[tiab] OR "Glypican2"[tiab] OR "Biglycan"[tiab] OR "Decorin"[tiab] OR "Fibromodulin"[tiab] OR "Lumican"[tiab] OR "Fibrillar Collagen"[tiab] OR "Collagen Type I"[tiab] OR "Collagen Type II"[tiab] OR "Collagen Type III"[tiab] OR "Collagen Type V"[tiab] OR "Collagen Type XI"[tiab] OR "Non-Fibrillar Collagen"[tiab] OR "Collagen Type IV"[tiab] OR "Collagen Type VI"[tiab] OR "Collagen Type VII"[tiab] OR "Collagen Type VIII"[tiab] OR "Collagen Type X"[tiab] OR "Collagen Type XIII"[tiab] OR "Collagen Type XVIII +"[tiab] OR "Fibril-Associated Collagens"[tiab] OR "Collagen Type IX"[tiab] OR "Collagen Type XII"[tiab] OR "Endostatins"[tiab] OR "Endostatin"[tiab] OR "Fibrillin"[tiab] OR "Fibronectins"[tiab] OR "Fibronectin"[tiab]
#4	Animal models<br><br>Hits: 7,660,699	(animal experimentation[MeSH] OR models, animal[MeSH] OR Animals[Mesh:noexp] OR animal population groups [MeSH] OR chordata[MeSH Terms:noexp] OR vertebrates[MeSH Terms:noexp] OR amphibians[MeSH] OR birds[MeSH] OR fishes[MeSH] OR reptiles[MeSH] OR mammals[MeSH Terms:noexp] OR primates[MeSH Terms:noexp] OR eutheria[MeSH Terms:noexp] OR artiodactyla[MeSH] OR carnivore[MeSH] OR cephalopoda[MeSH] OR cetacea[MeSH] OR chiroptera[MeSH] OR elephants[MeSH] OR hyraxes[MeSH] OR insectivora[MeSH] OR lagomorpha[MeSH] OR marsupialia[MeSH] OR monotremata[MeSH] OR perissodactyla[MeSH] OR Proboscidea Mammal[MeSH Terms:noexp] OR rodentia[MeSH] OR scandentia[MeSH] OR sirenia[MeSH] OR cingulata[MeSH] OR haplorhini[MeSH Terms:noexp] OR strepsirhini[MeSH] OR platyrrhini[MeSH] OR tarsii[MeSH] OR catarrhini[MeSH Terms:noexp] OR cercopithecidae[MeSH] OR hylobatidae[MeSH] OR hominidae[MeSH Terms:noexp] OR gorilla gorilla[MeSH] OR pan paniscus[MeSH] OR pan troglodytes[MeSH] OR pongo[MeSH]) OR ((rat[tiab] OR rats[tiab] OR animal[tiab] OR animals[tiab] OR mice[tiab] OR in vivo[tiab] OR mouse[tiab] OR rabbit[tiab] OR rabbits[tiab] OR murine[tiab] OR pig[tiab] OR pigs[tiab] OR dog[tiab] OR dogs[tiab] OR bovine[tiab] OR fish[tiab] OR vertebrate[tiab] OR vertebrates[tiab] OR cat[tiab] OR cats[tiab] OR rodent[tiab] OR rodents[tiab] OR mammal[tiab] OR mammals[tiab] OR chicken[tiab] OR chickens[tiab] OR monkey[tiab] OR monkeys[tiab] OR sheep[tiab] OR canine[tiab] OR canines[tiab] OR porcine[tiab] OR cattle[tiab] OR bird[tiab] OR birds[tiab] OR hamster[tiab] OR hamsters[tiab] OR primate[tiab] OR primates[tiab] OR cow[tiab] OR cows[tiab] OR chick[tiab] OR horse[tiab] OR horses[tiab] OR avian[tiab] OR avians[tiab] OR calf[tiab] OR swine[tiab] OR swines[tiab] OR xenopus[tiab] OR turkeys[tiab] OR bear[tiab] OR bears[tiab] OR frog[tiab] OR frogs[tiab] OR zebrafish[tiab] OR goat[tiab] OR goats[tiab] OR equine[tiab] OR calves[tiab] OR poultry[tiab] OR macaque[tiab] OR macaques[tiab] OR mole[tiab] OR moles[tiab] OR ovine[tiab] OR lamb[tiab] OR lambs[tiab] OR fishes[tiab] OR diptera[tiab] OR amphibian[tiab] OR amphibians[tiab] OR snake[tiab] OR snakes[tiab] OR ruminant[tiab] OR ruminants[tiab] OR hen[tiab] OR hens[tiab] OR piglet[tiab] OR piglets[tiab] OR feline[tiab] OR felines[tiab] OR simian[tiab] OR simians[tiab] OR laevis[tiab] OR trout[tiab] OR trouts[tiab] OR teleost[tiab] OR teleosts[tiab] OR salmon[tiab] OR salmons[tiab] OR seal[tiab] OR seals[tiab] OR bull[tiab] OR bulls[tiab] OR ewe[tiab] OR ewes[tiab] OR hedgehog[tiab] OR hedgehogs[tiab] OR macaca[tiab] OR macacas[tiab] OR proteus[tiab] OR pigeon[tiab] OR pigeons[tiab] OR bat[tiab] OR bats[tiab] OR duck[tiab] OR ducks[tiab] OR chimpanzee[tiab] OR chimpanzees[tiab] OR baboon[tiab] OR baboons[tiab] OR deer[tiab] OR rana[tiab] OR ranas[tiab] OR carp[tiab] OR carps[tiab] OR heifer[tiab] OR swallow[tiab] OR swallows[tiab] OR lizard[tiab] OR lizards[tiab] OR canis[tiab] OR sow[tiab] OR sows[tiab] OR cynomolgus[tiab] OR quail[tiab] OR quails[tiab] OR reptile[tiab] OR reptiles[tiab] OR turtle[tiab] OR turtles[tiab] OR buffalo[tiab] OR gerbil[tiab] OR gerbils[tiab] OR boar[tiab] OR boars[tiab] OR squirrel[tiab] OR squirrels[tiab] OR oncorhynchus[tiab] OR mus[tiab] OR toad[tiab] OR toads[tiab] OR fowl[tiab] OR fowls[tiab] OR rerio[tiab] OR danio[tiab] OR ara[tiab] OR aras[tiab] OR musculus[tiab] OR tadpole[tiab] OR tadpoles[tiab] OR mulatta[tiab] OR salmo[tiab] OR ram[tiab] OR eagle[tiab] OR eagles[tiab] OR ferret[tiab] OR ferrets[tiab] OR goldfish[tiab] OR catfish[tiab] OR whale[tiab] OR whales[tiab] OR fox[tiab] OR foxes[tiab] OR ape[tiab] OR apes[tiab] OR elephant[tiab] OR elephants[tiab] OR bos[tiab] OR marmoset[tiab] OR marmosets[tiab] OR cod[tiab] OR cods[tiab] OR shark[tiab] OR sharks[tiab] OR wolf[tiab] OR eel[tiab] OR eels[tiab] OR auratus[tiab] OR rattus[tiab] OR zebra[tiab] OR zebras[tiab] OR tilapia[tiab] OR tilapias[tiab] OR gilt[tiab] OR camel[tiab] OR camels[tiab] OR squid[tiab] OR gallus[tiab] OR marsupial[tiab] OR marsupials[tiab] OR vole[tiab] OR voles[tiab] OR fascicularis[tiab] OR ovis[tiab] OR salmonid[tiab] OR salmonids[tiab] OR tiger[tiab] OR tigers[tiab] OR dolphin[tiab] OR dolphins[tiab] OR robin[tiab] OR robins[tiab] OR carpio[tiab] OR opossum[tiab] OR opossums[tiab] OR cyprinus[tiab] OR salamander[tiab] OR salamanders[tiab] OR felis[tiab] OR mink[tiab] OR minks[tiab] OR swan[tiab] OR swans[tiab] OR norvegicus[tiab] OR bufo[tiab] OR torpedo[tiab] OR bass[tiab] OR lamprey[tiab] OR lampreys[tiab] OR sus[tiab] OR python[tiab] OR pythons[tiab] OR tetrapod[tiab] OR tetrapods[tiab] OR shrew[tiab] OR shrews[tiab] OR lion[tiab] OR lions[tiab] OR hog[tiab] OR hogs[tiab] OR songbird[tiab] OR songbirds[tiab] OR oreochromis[tiab] OR starling[tiab] OR starlings[tiab] OR caprine[tiab] OR carassius[tiab] OR owl[tiab] OR owls[tiab] OR newt[tiab] OR newts[tiab] OR papio[tiab] OR scrofa[tiab] OR hare[tiab] OR hares[tiab] OR gorilla[tiab] OR gorillas[tiab] OR flounder[tiab] OR flounders[tiab] OR goose[tiab] OR herring[tiab] OR herrings[tiab] OR therian[tiab] OR buffaloes[tiab] OR canary[tiab] OR sparrow[tiab] OR sparrows[tiab] OR microtus[tiab] OR octopus[tiab] OR troglodytes[tiab] OR tuna[tiab] OR

		amphibia[tiab] OR chinchilla[tiab] OR chinchillas[tiab] OR ide[tiab] OR oryzias[tiab] OR cervus[tiab] OR kangaroo[tiab] OR kangaroos[tiab] OR armadillo[tiab] OR armadillos[tiab] OR callithrix[tiab] OR pan troglodytes[tiab] OR saimiri[tiab] OR cichlid[tiab] OR cichlids[tiab] OR donkey[tiab] OR donkeys[tiab] OR bream[tiab] OR char[tiab] OR chars[tiab] OR finch[tiab] OR raccoon[tiab] OR raccoons[tiab] OR bothrops[tiab] OR anguilla[tiab] OR perch[tiab] OR cricetus[tiab] OR seabird[tiab] OR seabirds[tiab] OR buck[tiab] OR bucks[tiab] OR naja[tiab] OR coturnix[tiab] OR salmonids[tiab] OR geese[tiab] OR minnow[tiab] OR minnows[tiab] OR raptor[tiab] OR raptors[tiab] OR merione[tiab] OR meriones[tiab] OR rodentia[tiab] OR elaphus[tiab] OR amniote[tiab] OR amniotes[tiab] OR elasmobranch[tiab] OR emu[tiab] OR emus[tiab] OR peromyscus[tiab] OR hominid[tiab] OR hominids[tiab] OR bubalus[tiab] OR crotalus[tiab] OR gull[tiab] OR gulls[tiab] OR anas[tiab] OR anura[tiab] OR lemur[tiab] OR lemurs[tiab] OR crow[tiab] OR crows[tiab] OR camelus[tiab] OR gibbon[tiab] OR gibbons[tiab] OR waterfowl[tiab] OR parrot[tiab] OR parrots[tiab] OR eels[tiab] OR cob[tiab] OR stickleback[tiab] OR sticklebacks[tiab] OR columba[tiab] OR mesocricetus[tiab] OR ambystoma[tiab] OR raven[tiab] OR ravens[tiab] OR gadus[tiab] OR penguin[tiab] OR penguins[tiab] OR orangutan[tiab] OR orangutans[tiab] OR sturgeon[tiab] OR sturgeons[tiab] OR cuniculus[tiab] OR aves[tiab] OR virginianus[tiab] OR cephalopod[tiab] OR cephalopods[tiab] OR cebus[tiab] OR sparus[tiab] OR tortoise[tiab] OR tortoises[tiab] OR guttata[tiab] OR morhua[tiab] OR unguiculatus[tiab] OR dogfish[tiab] OR vulpes[tiab] OR mallard[tiab] OR mallards[tiab] OR apodemus[tiab] OR alligator[tiab] OR alligators[tiab] OR oryctolagus[tiab] OR llama[tiab] OR llamas[tiab] OR reindeer[tiab] OR mustela[tiab] OR duckling[tiab] OR ducklings[tiab] OR wolves[tiab] OR sander[tiab] OR amazona[tiab] OR zebu[tiab] OR badger[tiab] OR badgers[tiab] OR dove[tiab] OR doves[tiab] OR ictalurus[tiab] OR capra[tiab] OR capras[tiab] OR equus[tiab] OR camelid[tiab] OR camelids[tiab] OR poecilia[tiab] OR mule[tiab] OR mules[tiab] OR perciformes[tiab] OR salvelinus[tiab] OR labrax[tiab] OR cyprinidae[tiab] OR ariidae[tiab] OR crocodile[tiab] OR crocodiles[tiab] OR fundulus[tiab] OR dicentrarchus[tiab] OR clarias[tiab] OR cercopithecus[tiab] OR chiroptera[tiab] OR alpaca[tiab] OR alpacas[tiab] OR pike[tiab] OR pikes[tiab] OR paralichthys[tiab] OR puma[tiab] OR pumas[tiab] OR didelphis[tiab] OR pisces[tiab] OR macropus[tiab] OR triturus[tiab] OR bison[tiab] OR bisons[tiab] OR epinephelus[tiab] OR gasterosteus[tiab] OR panthera[tiab] OR acipenser[tiab] OR mackerel[tiab] OR mackerels[tiab] OR tamarin[tiab] OR tamarins[tiab] OR ostrich[tiab] OR anolis[tiab] OR vervet[tiab] OR vervets[tiab] OR wallaby[tiab] OR glareolus[tiab] OR beaver[tiab] OR beavers[tiab] OR dromedary[tiab] OR catus[tiab] OR killifish[tiab] OR pimephales[tiab] OR promelas[tiab] OR aotus[tiab] OR phoca[tiab] OR panda[tiab] OR pandas[tiab] OR porpoise[tiab] OR porpoises[tiab] OR myotis[tiab] OR yak[tiab] OR yaks[tiab] OR agkistrodon[tiab] OR vipera[tiab] OR otter[tiab] OR otters[tiab] OR turbot[tiab] OR turbots[tiab] OR squamate[tiab] OR carnivora[tiab] OR mullet[tiab] OR mullets[tiab] OR hawk[tiab] OR hawks[tiab] OR taeniopygia[tiab] OR seahorse[tiab] OR seahorses[tiab] OR poecilia reticulata[tiab] OR falcon[tiab] OR falcons[tiab] OR prosimian[tiab] OR prosimians[tiab] OR parus[tiab] OR perca[tiab] OR fingerling[tiab] OR fingerlings[tiab] OR antelope[tiab] OR antelopes[tiab] OR tupaia[tiab] OR passeriformes[tiab] OR sepia[tiab] OR saguinus[tiab] OR coyote[tiab] OR coyotes[tiab] OR pongo[tiab] OR meleagris[tiab] OR reptilia[tiab] OR lepus[tiab] OR psittacine[tiab] OR hagfish[tiab] OR warbler[tiab] OR warblers[tiab] OR russell's viper[tiab] OR russell's vipers[tiab] OR smolt[tiab] OR smolts[tiab] OR budgerigar[tiab] OR sardine[tiab] OR sardines[tiab] OR cavia[tiab] OR cavias[tiab] OR hyla[tiab] OR pleurodeles[tiab] OR siluriformes[tiab] OR great tit[tiab] OR great tits[tiab] OR guppy[tiab] OR bonobo[tiab] OR bonobos[tiab] OR rutilus[tiab] OR trichosurus[tiab] OR muridae[tiab] OR phodopus[tiab] OR channa[tiab] OR squalus[tiab] OR lynx[tiab] OR sturnus[tiab] OR petromyzon[tiab] OR vitulina[tiab] OR monodelphis[tiab] OR cuttlefish[tiab] OR adder[tiab] OR adders[tiab] OR lepomis[tiab] OR canaria[tiab] OR gambusia[tiab] OR guppies[tiab] OR xiphophorus[tiab] OR flatfish[tiab] OR koala[tiab] OR koalas[tiab] OR labeo[tiab] OR stingray[tiab] OR stingrays[tiab] OR chelonia[tiab] OR lampetra[tiab] OR spermophilus[tiab] OR crocodilian[tiab] OR passer domesticus[tiab] OR sciurus[tiab] OR artiodactyla[tiab] OR ranidae[tiab] OR corvus[tiab] OR necturus[tiab] OR platypus[tiab] OR canaries[tiab] OR bovid[tiab] OR lagopus[tiab] OR trimeresurus[tiab] OR gariepinus[tiab] OR marten[tiab] OR martens[tiab] OR drosophilidae[tiab] OR mugil[tiab] OR sunfish[tiab] OR porcellus[tiab] OR cypriniformes[tiab] OR alouatta[tiab] OR scophthalmus[tiab] OR anser[tiab] OR electrophorus[tiab] OR putorius[tiab] OR iguana[tiab] OR iguanas[tiab] OR lama[tiab] OR lamas[tiab] OR takifugu[tiab] OR circus[tiab] OR eptesicus[tiab] OR flycatcher[tiab] OR galago[tiab] OR galagos[tiab] OR trachemys[tiab] OR lungfish[tiab] OR characiformes[tiab] OR shorebird[tiab] OR shorebirds[tiab] OR giraffe[tiab] OR giraffes[tiab] OR micropterus[tiab] OR scyliorhinus[tiab] OR cichlidae[tiab] OR loligo[tiab] OR porcupine[tiab] OR porcupines[tiab] OR chub[tiab] OR chubs[tiab] OR solea[tiab] OR pleuronectes[tiab] OR hylidae[tiab] OR viperidae[tiab] OR echis[tiab] OR sorex[tiab] OR anchovy[tiab] OR lagomorph[tiab] OR ostriches[tiab] OR vulture[tiab] OR vultures[tiab] OR whitefish[tiab] OR araneus[tiab] OR jird[tiab] OR jirds[tiab] OR tern[tiab] OR esox[tiab] OR drake[tiab] OR drakes[tiab] OR elapidae[tiab] OR gallopavo[tiab] OR chordata[tiab] OR myodes[tiab] OR caretta[tiab] OR serinus[tiab] OR grouse[tiab] OR misgurnus[tiab] OR meles[tiab] OR blackbird[tiab] OR blackbirds[tiab] OR coregonus[tiab] OR bobwhite[tiab] OR bobwhites[tiab] OR heteropneustes[tiab] OR mammoth[tiab] OR mammoths[tiab] OR turdus[tiab] OR rhinella[tiab] OR ateles[tiab] OR characidae[tiab] OR clupea[tiab] OR bungarus [tiab] OR brill[tiab] OR struthio camelus[tiab] OR sloth[tiab] OR sloths[tiab] OR pteropus[tiab] OR sculpin[tiab] OR anthropoids[tiab] OR pollock[tiab] OR pollocks[tiab] OR morone[tiab] OR pan paniscus[tiab] OR litoria[tiab] OR chipmunk[tiab] OR chipmunks[tiab] OR balaenoptera[tiab] OR marmota[tiab] OR melopsittacus[tiab] OR hyrax[tiab] OR lemming[tiab] OR lemmings[tiab] OR halibut[tiab] OR hylobates[tiab] OR lates[tiab] OR caiman[tiab] OR caimans[tiab] OR sigmodon[tiab] OR stenella[tiab] OR barbel[tiab] OR barbels[tiab] OR sterna[tiab] OR parakeet[tiab] OR parakeets[tiab] OR phocoena[tiab] OR leptodactylus[tiab] OR canidae[tiab] OR buteo[tiab] OR harengus[tiab] OR gopher[tiab] OR gophers[tiab] OR marmot[tiab] OR marmots[tiab] OR gosling[tiab] OR goslings[tiab] OR platichthys[tiab] OR gar[tiab] OR gars[tiab] OR sebastes[tiab] OR marsupialia[tiab] OR notophthalmus[tiab] OR gazelle[tiab] OR gazelles[tiab] OR insectivora[tiab] OR paridae[tiab] OR felidae[tiab] OR russula[tiab] OR galliformes[tiab] OR bombina[tiab] OR colobus [tiab] OR echidna[tiab] OR echidnas[tiab] OR seabass[tiab] OR syncerus[tiab] OR plaice[tiab] OR blue tit[tiab] OR blue tits[tiab] OR pagrus[tiab] OR catfishes[tiab] OR cetacea[tiab] OR barbus[tiab] OR cygnus[tiab] OR ficedula[tiab] OR chamois[tiab] OR colubridae[tiab] OR perches[tiab] OR coelacanth[tiab] OR fitch[tiab] OR urodela[tiab] OR cynops[tiab] OR martes[tiab] OR halichoerus[tiab] OR aix[tiab] OR salmonidae[tiab] OR leuciscus[tiab] OR magpie[tiab] OR magpies[tiab] OR silurus[tiab] OR whiting[tiab] OR whitings[tiab] OR anseriformes[tiab] OR colinus[tiab] OR rhea[tiab] OR chlorocebus[tiab] OR octodon[tiab] OR acinonyx[tiab] OR mouflon[tiab] OR mouflons[tiab] OR ibex[tiab] OR tetraodon[tiab] OR bufonidae[tiab] OR equidae[tiab] OR jackal[tiab] OR cephalopoda[tiab] OR dendroaspis[tiab] OR glama[tiab] OR muskrat[tiab] OR muskrats[tiab] OR sable[tiab] OR sables[tiab] OR wildebeest[tiab] OR streptopelia[tiab] OR albifrons[tiab] OR vespertilionidae[tiab] OR woodpecker[tiab] OR woodpeckers[tiab] OR muntjac[tiab] OR muntjacs[tiab] OR archosaur[tiab] OR branta[tiab] OR cricetulus[tiab] OR megalobrama[tiab] OR poeciliidae[tiab] OR desmodus[tiab] OR snakehead[tiab] OR snakeheads[tiab] OR tench[tiab] OR teal[tiab] OR teals[tiab] OR bandicoot[tiab] OR bandicoots[tiab] OR apteronotus[tiab] OR phyllostomidae[tiab] OR crocidura[tiab] OR buzzard[tiab] OR buzzards[tiab] OR larimichthys[tiab] OR cercocebus[tiab] OR pipistrellus[tiab] OR erithacus[tiab] OR impala[tiab] OR impalas[tiab] OR rousettus[tiab] OR haddock[tiab] OR haddocks[tiab] OR tinca[tiab] OR ratite[tiab] OR calidris[tiab] OR cynoglossus[tiab] OR hypophthalmichthys[tiab] OR bullock[tiab] OR bullocks[tiab] OR dromedaries[tiab] OR alectoris[tiab] OR filly[tiab] OR salamandra[tiab] OR cingulata[tiab] OR bitis[tiab] OR grus[tiab] OR ammodytes[tiab] OR macaw[tiab] OR macaws[tiab] OR hypoleuca[tiab] OR sapajus[tiab] OR cyprinodontiformes[tiab] OR hippopotamus[tiab] OR pelophylax[tiab] OR capybara[tiab] OR capybaras[tiab] OR weasel[tiab] OR weasels[tiab] OR cairina[tiab] OR cynomys[tiab] OR lutra[tiab] OR cockatoo[tiab] OR

		cockatoos[tiab] OR lachesis[tiab] OR lagomorpha[tiab] OR rupicapra[tiab] OR daboia[tiab] OR orang utan[tiab] OR orang utans[tiab] OR platyrrhini[tiab] OR charadriiformes[tiab] OR micrurus[tiab] OR psittaciformes[tiab] OR spalax[tiab] OR loris[tiab] OR mustelidae[tiab] OR sylvilagus[tiab] OR vitticeps[tiab] OR cockatiel[tiab] OR mustelus[tiab] OR cottus[tiab] OR erythrocebus[tiab] OR dipodomys[tiab] OR platessa[tiab] OR callicebus[tiab] OR loricariidae[tiab] OR catostomus[tiab] OR cuneata[tiab] OR cyanistes[tiab] OR cyprinodon[tiab] OR sigmodontinae[tiab] OR elasmobranchii[tiab] OR trichechus[tiab] OR sauropsid[tiab] OR xenarthra[tiab] OR dormouse[tiab] OR perissodactyla[tiab] OR nautilus[tiab] OR cirrhinus[tiab] OR gulo[tiab] OR tragelaphus[tiab] OR merula[tiab] OR numida[tiab] OR sciaenidae[tiab] OR cerastes[tiab] OR sciuridae[tiab] OR gibbosus[tiab] OR octopuses[tiab] OR eland[tiab] OR elands[tiab] OR phyllomedusa[tiab] OR pogona[tiab] OR walrus[tiab] OR agamidae[tiab] OR leptodactylidae[tiab] OR ridibundus[tiab] OR leontopithecus[tiab] OR anteater[tiab] OR anteaters[tiab] OR pelodiscus[tiab] OR cebidae[tiab] OR columbianus[tiab] OR pelteobagrus fulvidraco[tiab] OR hominoidea[tiab] OR mandrillus[tiab] OR zonotrichia leucophrys[tiab] OR agama[tiab] OR gobiocypris[tiab] OR bearded dragon[tiab] OR bearded dragons[tiab] OR sarotherodon[tiab] OR talpa[tiab] OR discoglossus[tiab] OR hagfishes[tiab] OR sphenodon[tiab] OR gudgeon[tiab] OR amphiuma[tiab] OR aythya[tiab] OR tenrec[tiab] OR tenrec[tiab] OR hominidae[tiab] OR risoria[tiab] OR salamandridae[tiab] OR camelidae[tiab] OR columbiformes[tiab] OR latimeria[tiab] OR plover[tiab] OR plovers[tiab] OR afrotheria[tiab] OR falco sparverius[tiab] OR polecat[tiab] OR polecats[tiab] OR crotalinae[tiab] OR salvadora[tiab] OR tarsier[tiab] OR lucioperca[tiab] OR anchovies[tiab] OR lungfishes[tiab] OR terrapin[tiab] OR dromaius novaehollandiae[tiab] OR lateolabrax[tiab] OR eigenmannia[tiab] OR pelamis[tiab] OR theropithecus[tiab] OR murinae[tiab] OR gander[tiab] OR gymnotus[tiab] OR pseudacris[tiab] OR gymnophiona[tiab] OR gymnotiformes[tiab] OR laticauda[tiab] OR falconiformes[tiab] OR dugong[tiab] OR dugongs[tiab] OR pintail[tiab] OR pintails[tiab] OR rook[tiab] OR rooks[tiab] OR lasiurus[tiab] OR catshark[tiab] OR catsharks[tiab] OR micropogonias[tiab] OR red junglefowl[tiab] OR paddlefish[tiab] OR ophiophagus[tiab] OR hollandicus[tiab] OR nymphicus[tiab] OR pimelodidae[tiab] OR aepyceros[tiab] OR cobitidae[tiab] OR strigiformes[tiab] OR cobitis[tiab] OR dormice[tiab] OR alytes[tiab] OR calloselasma[tiab] OR guanaco[tiab] OR phasianidae[tiab] OR round goby[tiab] OR trichogaster[tiab] OR catarrhini[tiab] OR eelpout[tiab] OR eelpouts[tiab] OR galaxias[tiab] OR gaur[tiab] OR pungitius[tiab] OR suslik[tiab] OR susliks[tiab] OR flatfishes[tiab] OR percidae[tiab] OR caprinae[tiab] OR todarodes[tiab] OR osmerus[tiab] OR ameiurus[tiab] OR anthropoidea[tiab] OR castor canadensis[tiab] OR pouting[tiab] OR poutings[tiab] OR tetraodontiformes[tiab] OR arvicolinae[tiab] OR siamang[tiab] OR siamangs[tiab] OR castor fiber[tiab] OR nomascus[tiab] OR red knot[tiab] OR red knots[tiab] OR syngnathidae[tiab] OR iguanidae[tiab] OR eretmochelys[tiab] OR ursidae[tiab] OR callimico[tiab] OR columbidae[tiab] OR microhylidae[tiab] OR anaxyrus[tiab] OR menidia[tiab] OR pipistrelle[tiab] OR greylag[tiab] OR pipidae[tiab] OR scandentia[tiab] OR bowfin[tiab] OR bowfins[tiab] OR dendrobatidae[tiab] OR zenaida[tiab] OR bushbaby[tiab] OR harrier[tiab] OR harriers[tiab] OR macropodidae[tiab] OR pygerythrus[tiab] OR clupeidae[tiab] OR odorrana[tiab] OR corvidae[tiab] OR jerboa[tiab] OR jerboas[tiab] OR canutus[tiab] OR hylobatidae[tiab] OR clupeiformes[tiab] OR great cormorant[tiab] OR great cormorants[tiab] OR scorpaeniformes[tiab] OR chondrostean[tiab] OR garfish[tiab] OR proboscidea[tiab] OR psetta[tiab] OR diapsid[tiab] OR serotinus[tiab] OR tetrao[tiab] OR walruses[tiab] OR carcharhiniformes[tiab] OR leucoraja[tiab] OR pumpkinseed[tiab] OR dosidicus[tiab] OR acipenseriformes[tiab] OR daubentonii[tiab] OR emberizidae[tiab] OR gadiformes[tiab] OR hyraxes[tiab] OR stizostedion[tiab] OR wolverine[tiab] OR wolverines[tiab] OR lissotriton[tiab] OR acanthurus[tiab] OR centrarchidae[tiab] OR gloydius[tiab] OR laurasiatheria[tiab] OR limosa[tiab] OR psittacula[tiab] OR leporidae[tiab] OR proteidae[tiab] OR zander[tiab] OR zanders[tiab] OR arapaima[tiab] OR bagridae[tiab] OR cyprinodontidae[tiab] OR mithun[tiab] OR pandion[tiab] OR jackdaw[tiab] OR jackdaws[tiab] OR procyonidae[tiab] OR carus[tiab] OR jaculus[tiab] OR salmoniformes[tiab] OR common sole[tiab] OR common soles[tiab] OR protobothrops[tiab] OR calamita[tiab] OR brachyteles[tiab] OR trionyx[tiab] OR turdidae[tiab] OR boidae[tiab] OR luscinia[tiab] OR pugnax[tiab] OR euarchontoglires[tiab] OR saithe[tiab] OR saithes[tiab] OR symphalangus[tiab] OR aardvark[tiab] OR aardvarks[tiab] OR oystercatcher[tiab] OR oystercatchers[tiab] OR arius[tiab] OR corydoras[tiab] OR poacher[tiab] OR poachers[tiab] OR aurochs[tiab] OR cebuella[tiab] OR crecca[tiab] OR lemuridae[tiab] OR sirenia[tiab] OR lemmus[tiab] OR perdix[tiab] OR glires[tiab] OR lepidosaur[tiab] OR muskox[tiab] OR deinagkistrodon[tiab] OR pholidota[tiab] OR holocephali[tiab] OR cercopithecinae[tiab] OR clariidae[tiab] OR agapornis[tiab] OR doryteuthis[tiab] OR tyrannidae[tiab] OR dicroglossidae[tiab] OR godwit[tiab] OR godwits[tiab] OR monedula[tiab] OR pongidae[tiab] OR atheriniformes[tiab] OR colobinae[tiab] OR lophocebus[tiab] OR atelidae[tiab] OR cottidae[tiab] OR leucopsis[tiab] OR acanthuridae[tiab] OR didelphimorphia[tiab] OR elver[tiab] OR elvers[tiab] OR lapponica[tiab] OR dermoptera[tiab] OR “european hake”[tiab] OR “european hakes”[tiab] OR gerbillinae[tiab] OR banteng[tiab] OR hartebeest[tiab] OR hartebeests[tiab] OR hogget[tiab] OR haematopus[tiab] OR anguis fragilis[tiab] OR grey heron[tiab] OR grey herons[tiab] OR “blue whiting”[tiab] OR “blue whitings”[tiab] OR furnariidae[tiab] OR macrovipera[tiab] OR esocidae[tiab] OR lapwing[tiab] OR lapwings[tiab] OR mylopharyngodon[tiab] OR wallabia[tiab] OR beloniformes[tiab] OR potoroo[tiab] OR potoroos[tiab] OR athene noctua[tiab] OR pleuronectidae[tiab] OR bushbabies[tiab] OR muscicapidae[tiab] OR alligatoridae[tiab] OR fuligula[tiab] OR bush baby[tiab] OR guineafowl[tiab] OR spoonbill[tiab] OR spoonbills[tiab] OR viverridae[tiab] OR catostomidae[tiab] OR zebrafishes[tiab] OR ibexes[tiab] OR vendace[tiab] OR estrildidae[tiab] OR monotremata[tiab] OR sepiella[tiab] OR ambystomatidae[tiab] OR shelduck[tiab] OR shelducks[tiab] OR treeshrew[tiab] OR treeshrews[tiab] OR hoplobatrachus[tiab] OR pochard[tiab] OR hoolock[tiab] OR hoolocks[tiab] OR lynxes[tiab] OR antilope[tiab] OR antilopes[tiab] OR blackbuck[tiab] OR blackbucks[tiab] OR cricetinae[tiab] OR paramisgurnus[tiab] OR skylark[tiab] OR skylarks[tiab] OR soleidae[tiab] OR allobates[tiab] OR northern wheatear[tiab] OR northern wheatears[tiab] OR pitheciidae[tiab] OR takin[tiab] OR theria[tiab] OR vanellus[tiab] OR galaxiidae[tiab] OR lorisidae[tiab] OR ostralegus[tiab] OR palaeognathae[tiab] OR stone loach[tiab] OR alauda[tiab] OR callitrichinae[tiab] OR caniformia[tiab] OR duttaphrynus[tiab] OR ictaluridae[tiab] OR osteoglossiformes[tiab] OR poultries[tiab] OR curema[tiab] OR ruddy turnstone[tiab] OR ruddy turnstones[tiab] OR sheatfish[tiab] OR sunfishes[tiab] OR centropomidae[tiab] OR hemachatus[tiab] OR platalea[tiab] OR thamnophilidae[tiab] OR song thrush[tiab] OR atherinopsidae[tiab] OR siluridae[tiab] OR tadorna[tiab] OR chroicocephalus[tiab] OR ermine[tiab] OR ermines[tiab] OR gavialis[tiab] OR ruff[tiab] OR tupaiidae[tiab] OR diprotodontia[tiab] OR hyaenidae[tiab] OR antilopinae[tiab] OR crocodylidae[tiab] OR herpestidae[tiab] OR hippopotamidae[tiab] OR northern shoveler[tiab] OR round gobies[tiab] OR cheirogaleidae[tiab] OR indriidae[tiab] OR fundulidae[tiab] OR pythonidae[tiab] OR rhynchocephalia[tiab] OR anodorhynchus[tiab] OR red-backed shrike[tiab] OR red-backed shrikes[tiab] OR triakidae[tiab] OR phalangeridae[tiab] OR aoudad[tiab] OR boreoeutheria[tiab] OR eurasian jay[tiab] OR eurasian jays[tiab] OR feliformia[tiab] OR haplorhini[tiab] OR osteoglossidae[tiab] OR paenungulata[tiab] OR struthioniformes[tiab] OR ferina[tiab] OR sanderling[tiab] OR sanderlings[tiab] OR spheniscidae[tiab] OR cuttlefishes[tiab] OR cygnet[tiab] OR dasycneme[tiab] OR gadwall[tiab] OR gadwalls[tiab] OR pelobates fuscus[tiab] OR wryneck[tiab] OR wrynecks[tiab] OR afrosoricida[tiab] OR culaea[tiab] OR “dover sole”[tiab] OR “dover soles”[tiab] OR paralichthyidae[tiab] OR passeridae[tiab] OR osteolaemus[tiab] OR song thrushes[tiab] OR bluethroat[tiab] OR bluethroats[tiab] OR hydrophiidae[tiab] OR megrim[tiab] OR mephitidae[tiab] OR strepsirhini[tiab] OR tomistoma[tiab] OR epidalea[tiab] OR osmeriformes[tiab] OR bush babies[tiab] OR tarsiiform[tiab] OR atelinae[tiab] OR bufotes[tiab] OR eurasian coot[tiab] OR eurasian coots[tiab] OR galagidae[tiab] OR geopelia[tiab] OR philomachus[tiab] OR tubulidentata[tiab] OR bombinatoridae[tiab] OR pelobatidae[tiab] OR tachysurus[tiab] OR ailuridae[tiab] OR woodlark[tiab] OR woodlarks[tiab] OR alcelaphinae[tiab] OR redshank[tiab] OR redshanks[tiab] OR salientia[tiab] OR sand smelt[tiab] OR

		sand smelts[tiab] OR woodmice[tiab] OR woodmouse[tiab] OR dasyproctidae[tiab] OR eurasian wigeon[tiab] OR eurasian wigeons[tiab] OR garganey[tiab] OR garganeys[tiab] OR “lemon sole”[tiab] OR “lemon soles”[tiab] OR “common dab”[tiab] OR “common dabs”[tiab] OR graylag[tiab] OR graylags[tiab] OR leucorodia[tiab] OR osphronemidae[tiab] OR bewickii[tiab] OR common moorhen[tiab] OR common moorhens[tiab] OR decapodiformes[tiab] OR gobbler[tiab] OR gobblers[tiab] OR odontophoridae[tiab] OR paddlefishes[tiab] OR eutheria[tiab] OR salmonine[tiab] OR esociformes[tiab] OR eurasian woodcock[tiab] OR eurasian woodcocks[tiab] OR “european smelt”[tiab] OR “european smelts”[tiab] OR goldfishes[tiab] OR tenches[tiab] OR tyranni[tiab] OR common chaffinch[tiab] OR common chaffinchs[tiab] OR common redstart[tiab] OR common redstarts[tiab] OR “common roach”[tiab] OR “common roachs”[tiab] OR great knot[tiab] OR great knots[tiab] OR potoroidae[tiab] OR alytidae[tiab] OR coregonine[tiab] OR dipteral[tiab] OR leveret[tiab] OR poeciliopsis gracilis[tiab] OR amphiumidae[tiab] OR batrachoidiformes[tiab] OR bighead goby[tiab] OR heteropneustidae[tiab] OR lullula[tiab] OR “norway pout”[tiab] OR “norway pouts”[tiab] OR sipunculida[tiab] OR dogfishes[tiab] OR sebastidae[tiab] OR tarsiidae[tiab] OR alethinophidia[tiab] OR “common nase”[tiab] OR “common nases”[tiab] OR “common sandpiper”[tiab] OR “common sandpipers”[tiab] OR eurasian blackcap[tiab] OR eurasian blackcaps[tiab] OR pterocnemia[tiab] OR syngnathiformes[tiab] OR common chaffinches[tiab] OR eupleridae[tiab] OR octopodiformes[tiab] OR phascolarctidae[tiab] OR scophthalmidae[tiab] OR “starry smooth-hound”[tiab] OR “starry smooth-hounds”[tiab] OR whitefishes[tiab] OR cuniculidae[tiab] OR “european sprat”[tiab] OR “european sprats”[tiab] OR “rosy bitterling”[tiab] OR “rosy bitterlings”[tiab] OR “common dace”[tiab] OR “common daces”[tiab] OR “lesser weever”[tiab] OR “lesser weevers”[tiab] OR scaldfish[tiab] OR water rail[tiab] OR water rails[tiab] OR alouattinae[tiab] OR centrarchiformes[tiab] OR “common whitethroat”[tiab] OR “common whitethroats”[tiab] OR gavialidae[tiab] OR “grey gurnard”[tiab] OR “grey gurnards”[tiab] OR lateolabracidae[tiab] OR rheiformes[tiab] OR “tub gurnard”[tiab] OR “tub gurnards”[tiab] OR “common chiffchaff”[tiab] OR “common chiffchaffs”[tiab] OR garfishes[tiab] OR “lesser whitethroat”[tiab] OR “lesser whitethroats”[tiab] OR myoxidae[tiab] OR seabasses[tiab] OR spariformes[tiab] OR umbridae[tiab] OR yellow boxfish[tiab] OR anabantiformes[tiab] OR aotidae[tiab] OR “common bleak”[tiab] OR “common bleaks”[tiab] OR “common rudd”[tiab] OR “common rudds”[tiab] OR greater pipefish[tiab] OR hapale[tiab] OR nandiniidae[tiab] OR “stone loaches”[tiab] OR whinchat[tiab] OR whinchats[tiab] OR acanthuriformes[tiab] OR brotula barbata[tiab] OR “common ling”[tiab] OR “common lings”[tiab] OR “common roaches”[tiab] OR cottonrat[tiab] OR cottonrats[tiab] OR douroucoulis[tiab] OR dromaiidae[tiab] OR fitches[tiab] OR fitchew[tiab] OR galaxiiformes[tiab] OR laprine[tiab] OR saimiriinae[tiab] OR solenette[tiab] OR tarsii[tiab] OR tompot blenny[tiab] OR “common dragonet”[tiab] OR “common dragonets”[tiab] OR “longspined bullhead”[tiab] OR “longspined bullheads”[tiab] OR monotremate[tiab] OR monotremates[tiab] OR pempheriformes[tiab] OR perdicinae[tiab] OR presbytini[tiab] OR smegmamorpha[tiab] OR “bighead gobies”[tiab] OR “carangaria incertae sedis”[tiab] OR coiidae[tiab] OR fivebeard rockling[tiab] OR foulmart[tiab] OR foumart[tiab] OR grasskeet[tiab] OR “greater pipefishes”[tiab] OR ibices[tiab] OR millionfish[tiab] OR muguliformes[tiab] OR “norwegian topknot”[tiab] OR peewit[tiab] OR “red sea sailfin tang”[tiab] OR rupicapras[tiab] OR sheatfishes[tiab] OR “tompot blennies”[tiab] OR “twait shad”[tiab] OR “yellow boxfishes”[tiab]) NOT medline[sb])
#5	Cardiac AND repair AND ECM and animals 2,784 hits	#1 AND #2 AND #3 AND #4
#6	Remove reviews 2236 hits	#5 NOT review[ptyp]
#7	*Retrieve reviews for separate screening* 548 hits	#5 AND review[ptyp]

Supplementary File 4 – full search strings for SCOPUS 06-07-2022

SCOPUS	06-07-2022	1528 hits without reviews
#1	Heart injury<br><br>Hits:<br>1,804,426	((TITLE-ABS("cardiac fibrosis") OR TITLE-ABS(heart) OR TITLE-ABS(hearts) OR TITLE-ABS(cardiac) OR TITLE-ABS(myocardial) OR TITLE-ABS(myocardium) OR TITLE-ABS(myocardia) OR TITLE-ABS(myocard)) OR ((TITLE-ABS(ischemia) OR TITLE-ABS(ischaemia) OR TITLE-ABS(ischemic) OR TITLE-ABS(ischaemic) OR TITLE-ABS(infarct) OR TITLE-ABS(infarcts) OR TITLE-ABS(infarction) OR TITLE-ABS(infarctions) OR TITLE-ABS(infarcted) OR TITLE-ABS("reperfusion injury") OR TITLE-ABS("reperfusion injuries") OR TITLE-ABS(IR) OR TITLE-ABS(IRI) OR TITLE-ABS(occlusion) OR TITLE-ABS(failure) OR TITLE-ABS(decompensation) OR TITLE-ABS(insufficiency) OR TITLE-ABS(incompetence) OR TITLE-ABS(overload) OR TITLE-ABS(transplantation) OR TITLE-ABS(transplantations) OR TITLE-ABS(transplant) OR TITLE-ABS(transplants) OR TITLE-ABS(graft) OR TITLE-ABS(grafts)) AND (TITLE-ABS(heart) OR TITLE-ABS(hearts) OR TITLE-ABS(cardiac) OR TITLE-ABS(myocardial) OR TITLE-ABS(myocardium) OR TITLE-ABS(myocardia) OR TITLE-ABS(myocard)))) AND NOT (TITLE-ABS("Heart Valves") OR TITLE-ABS("Heart Valve") OR TITLE-ABS("Aortic Valves") OR TITLE-ABS("Aortic Valve") OR TITLE-ABS("Chordae Tendineae") OR TITLE-ABS("Mitral Valves") OR TITLE-ABS("Mitral Valve") OR TITLE-ABS("Pulmonary Valves") OR TITLE-ABS("Pulmonary Valve") OR TITLE-ABS("Tricuspid Valves") OR TITLE-ABS("Tricuspid Valve") OR TITLE-ABS("Cardiac Valves") OR TITLE-ABS("Cardiac Valve"))
#2	Repair<br><br>Hits:<br>1,057,54	TITLE-ABS(regeneration) OR TITLE-ABS(regenerations) OR TITLE-ABS(regenerated) OR TITLE-ABS(regenerative) OR TITLE-ABS("wound healing") OR TITLE-ABS("wound healings") OR TITLE-ABS(renewal) OR TITLE-ABS(repair) OR TITLE-ABS("Tissue Engineering") OR TITLE-ABS("Tissue Scaffolds") OR TITLE-ABS("Tissue Scaffold")
#2	ECM<br><br>Hits:<br>581,405	TITLE-ABS("Extracellular Matrix Protein") OR TITLE-ABS("Extracellular Matrix Proteins") OR TITLE-ABS("Extracellular Matrix") OR TITLE-ABS("Extracellular Matrices") OR TITLE-ABS(ECM) OR TITLE-ABS(ECMs) OR TITLE-ABS("Decellularized Extracellular Matrix") OR TITLE-ABS("Decellularized Extracellular Matrices") OR TITLE-ABS("Decellularized ECM") OR TITLE-ABS("Decellularized ECMs") OR TITLE-ABS(Glycosaminoglycans) OR TITLE-ABS(Glycosaminoglycan) OR TITLE-ABS(Chondroitin) OR TITLE-ABS("Chondroitin Sulfates") OR TITLE-ABS("Chondroitin Sulfate") OR TITLE-ABS("Dermatan Sulfates") OR TITLE-ABS("Dermatan Sulfate") OR TITLE-ABS(Heparin) OR TITLE-ABS(Dalteparin) OR TITLE-ABS(Enoxaparin) OR TITLE-ABS(Nadroparin) OR TITLE-ABS(Tinzaparin) OR TITLE-ABS(Heparinoids) OR TITLE-ABS("Heparitin Sulfate") OR TITLE-ABS("Heparan Sulfate Proteoglycans") OR TITLE-ABS("Hyaluronic Acid") OR TITLE-ABS("Keratan Sulfate") OR TITLE-ABS("Biocompatible Materials") OR TITLE-ABS(Proteoglycans) OR TITLE-ABS(Proteoglycan) OR TITLE-ABS("Activated-Leukocyte Cell Adhesion Molecule") OR TITLE-ABS("ADAMTS Proteins") OR TITLE-ABS("ADAMTS1 Protein") OR TITLE-ABS("ADAMTS13 Protein") OR TITLE-ABS("ADAMTS4 Protein") OR TITLE-ABS("ADAMTS5 Protein") OR TITLE-ABS("ADAMTS7 Protein") OR TITLE-ABS("ADAMTS9 Protein") OR TITLE-ABS(Aggrecan) OR TITLE-ABS(Aggrecans) OR TITLE-ABS("Cartilage Oligomeric Matrix Protein") OR TITLE-ABS("Cartilage Oligomeric Matrix Proteins") OR TITLE-ABS("CCN Intercellular Signaling Protein") OR TITLE-ABS("CCN Intercellular Signaling Proteins") OR TITLE-ABS("Connective Tissue Growth Factor") OR TITLE-ABS("Connective Tissue Growth Factors") OR TITLE-ABS("Cysteine-Rich Protein 61") OR TITLE-ABS("Nephroblastoma Overexpressed Protein") OR TITLE-ABS(Collagen) OR TITLE-ABS(Collagens) OR TITLE-ABS("Fibrillar Collagens") OR TITLE-ABS("Non-Fibrillar Collagens") OR TITLE-ABS(Procollagen) OR TITLE-ABS(Tropocollagen) OR TITLE-ABS(Elastin) OR TITLE-ABS(Tropoelastin) OR TITLE-ABS(Fibrillins) OR TITLE-ABS(Fibrillin-1) OR TITLE-ABS(Fibrillin-2) OR TITLE-ABS(Fibronectins) OR TITLE-ABS("Integrin-Binding Sialoprotein") OR TITLE-ABS(Laminin) OR TITLE-ABS("Latent TGF-beta Binding Proteins") OR TITLE-ABS("Matrilin Proteins") OR TITLE-ABS(Netrins) OR TITLE-ABS(Netrin-1) OR TITLE-ABS(Osteopontin) OR TITLE-ABS("Reelin Protein") OR TITLE-ABS("Small Leucine-Rich Proteoglycans") OR TITLE-ABS(Biglycan) OR TITLE-ABS(Decorin) OR TITLE-ABS(Fibromodulin) OR TITLE-ABS(Lumican) OR TITLE-ABS(Tenascin) OR TITLE-ABS(Versicans) OR TITLE-ABS(Vitronectin) OR TITLE-ABS("Chondroitin Sulfate Proteoglycans") OR TITLE-ABS(Aggrecans) OR TITLE-ABS(Versicans) OR TITLE-ABS(Versican) OR TITLE-ABS("Small Leucine-Rich Proteoglycans") OR TITLE-ABS(Biglycan) OR TITLE-

		ABS(Decorin) OR TITLE-ABS(Fibromodulin) OR TITLE-ABS(Lumican) OR TITLE-ABS("Hyaluronan Receptors") OR TITLE-ABS("Hyaluronan Receptor") OR TITLE-ABS(Glypicans) OR TITLE-ABS(Syndecans) OR TITLE-ABS(Syndecan) OR TITLE-ABS(Syndecan-1) OR TITLE-ABS(Syndecan-2) OR TITLE-ABS(Syndecan-3) OR TITLE-ABS(Syndecan-4) OR TITLE-ABS(Syndecan1) OR TITLE-ABS(Syndecan2) OR TITLE-ABS(Syndecan3) OR TITLE-ABS(Syndecan4) OR TITLE-ABS(Glypican) OR TITLE-ABS(Glypican-5) OR TITLE-ABS(Glypican5) OR TITLE-ABS(Glypican-3) OR TITLE-ABS(Glypican3) OR TITLE-ABS(Glypican-4) OR TITLE-ABS(Glypican4) OR TITLE-ABS(Glypican-1) OR TITLE-ABS(Glypican1) OR TITLE-ABS(Glypican-2) OR TITLE-ABS(Glypican2) OR TITLE-ABS(Biglycan) OR TITLE-ABS(Decorin) OR TITLE-ABS(Fibromodulin) OR TITLE-ABS(Lumican) OR TITLE-ABS("Fibrillar Collagen") OR TITLE-ABS("Collagen Type I") OR TITLE-ABS("Collagen Type II") OR TITLE-ABS("Collagen Type III") OR TITLE-ABS("Collagen Type V") OR TITLE-ABS("Collagen Type XI") OR TITLE-ABS("Non-Fibrillar Collagen") OR TITLE-ABS("Collagen Type IV") OR TITLE-ABS("Collagen Type VI") OR TITLE-ABS("Collagen Type VII") OR TITLE-ABS("Collagen Type VIII") OR TITLE-ABS("Collagen Type X") OR TITLE-ABS("Collagen Type XIII") OR TITLE-ABS("Collagen Type XVIII +") OR TITLE-ABS("Fibril-Associated Collagens") OR TITLE-ABS("Collagen Type IX") OR TITLE-ABS("Collagen Type XII") OR TITLE-ABS(Endostatins) OR TITLE-ABS(Endostatin) OR TITLE-ABS(Fibrillin) OR TITLE-ABS(Fibronectins) OR TITLE-ABS(Fibronectin)
#4	Animal models<br><br>Hits: 11,370,503	(((TITLE-ABS(rat) OR TITLE-ABS(rats) OR TITLE-ABS(animal) OR TITLE-ABS(animals) OR TITLE-ABS(mice) OR TITLE-ABS("in vivo") OR TITLE-ABS(mouse) OR TITLE-ABS(rabbit) OR TITLE-ABS(rabbits) OR TITLE-ABS(murine) OR TITLE-ABS(pig) OR TITLE-ABS(pigs) OR TITLE-ABS(dog) OR TITLE-ABS(dogs) OR TITLE-ABS(bovine) OR TITLE-ABS(fish) OR TITLE-ABS(vertebrate) OR TITLE-ABS(vertebrates) OR TITLE-ABS(cat) OR TITLE-ABS(cats) OR TITLE-ABS(rodent) OR TITLE-ABS(rodents) OR TITLE-ABS(mammal) OR TITLE-ABS(mammals) OR TITLE-ABS(chicken) OR TITLE-ABS(chickens) OR TITLE-ABS(monkey) OR TITLE-ABS(monkeys) OR TITLE-ABS(sheep) OR TITLE-ABS(canine) OR TITLE-ABS(canines) OR TITLE-ABS(porcine) OR TITLE-ABS(cattle) OR TITLE-ABS(bird) OR TITLE-ABS(birds) OR TITLE-ABS(hamster) OR TITLE-ABS(hamsters) OR TITLE-ABS(primate) OR TITLE-ABS(primates) OR TITLE-ABS(cow) OR TITLE-ABS(cows) OR TITLE-ABS(chick) OR TITLE-ABS(horse) OR TITLE-ABS(horses) OR TITLE-ABS(avian) OR TITLE-ABS(avians) OR TITLE-ABS(calf) OR TITLE-ABS(swine) OR TITLE-ABS(swines) OR TITLE-ABS(xenopus) OR TITLE-ABS(turkeys) OR TITLE-ABS(bear) OR TITLE-ABS(bears) OR TITLE-ABS(frog) OR TITLE-ABS(frogs) OR TITLE-ABS(zebrafish) OR TITLE-ABS(goat) OR TITLE-ABS(goats) OR TITLE-ABS(equine) OR TITLE-ABS(calves) OR TITLE-ABS(poultry) OR TITLE-ABS(macaque) OR TITLE-ABS(macaques) OR TITLE-ABS(mole) OR TITLE-ABS(moles) OR TITLE-ABS(ovine) OR TITLE-ABS(lamb) OR TITLE-ABS(lambs) OR TITLE-ABS(fishes) OR TITLE-ABS(diptera) OR TITLE-ABS(amphibian) OR TITLE-ABS(amphibians) OR TITLE-ABS(snake) OR TITLE-ABS(snakes) OR TITLE-ABS(ruminant) OR TITLE-ABS(ruminants) OR TITLE-ABS(hen) OR TITLE-ABS(hens) OR TITLE-ABS(piglet) OR TITLE-ABS(piglets) OR TITLE-ABS(feline) OR TITLE-ABS(felines) OR TITLE-ABS(simian) OR TITLE-ABS(simians) OR TITLE-ABS(laevis) OR TITLE-ABS(trout) OR TITLE-ABS(trouts) OR TITLE-ABS(teleost) OR TITLE-ABS(teleosts) OR TITLE-ABS(salmon) OR TITLE-ABS(salmons) OR TITLE-ABS(seal) OR TITLE-ABS(seals) OR TITLE-ABS(bull) OR TITLE-ABS(bulls) OR TITLE-ABS(ewe) OR TITLE-ABS(ewes) OR TITLE-ABS(hedgehog) OR TITLE-ABS(hedgehogs) OR TITLE-ABS(macaca) OR TITLE-ABS(macacas) OR TITLE-ABS(proteus) OR TITLE-ABS(pigeon) OR TITLE-ABS(pigeons) OR TITLE-ABS(bat) OR TITLE-ABS(bats) OR TITLE-ABS(duck) OR TITLE-ABS(ducks) OR TITLE-ABS(chimpanzee) OR TITLE-ABS(chimpanzees) OR TITLE-ABS(baboon) OR TITLE-ABS(baboons) OR TITLE-ABS(deer) OR TITLE-ABS(rana) OR TITLE-ABS(ranas) OR TITLE-ABS(carp) OR TITLE-ABS(carps) OR TITLE-ABS(heifer) OR TITLE-ABS(swallow) OR TITLE-ABS(swallows) OR TITLE-ABS(lizard) OR TITLE-ABS(lizards) OR TITLE-ABS(canis) OR TITLE-ABS(sow) OR TITLE-ABS(sows) OR TITLE-ABS(cynomolgus) OR TITLE-ABS(quail) OR TITLE-ABS(quails) OR TITLE-ABS(reptile) OR TITLE-ABS(reptiles) OR TITLE-ABS(turtle) OR TITLE-ABS(turtles) OR TITLE-ABS(buffalo) OR TITLE-ABS(gerbil) OR TITLE-ABS(gerbils) OR TITLE-ABS(boar) OR TITLE-ABS(boars) OR TITLE-ABS(squirrel) OR TITLE-ABS(squirrels) OR TITLE-ABS(oncorhynchus) OR TITLE-ABS(mus) OR TITLE-ABS(toad) OR TITLE-ABS(toads) OR TITLE-ABS(fowl) OR TITLE-ABS(fowls) OR TITLE-ABS(rerio) OR TITLE-ABS(danio) OR TITLE-ABS(ara) OR TITLE-ABS(aras) OR TITLE-ABS(musculus) OR TITLE-ABS(tadpole) OR TITLE-ABS(tadpoles) OR TITLE-ABS(mulatta) OR TITLE-ABS(salmo) OR TITLE-ABS(ram) OR TITLE-ABS(eagle) OR TITLE-ABS(eagles) OR TITLE-ABS(ferret) OR TITLE-ABS(ferrets) OR TITLE-ABS(goldfish) OR TITLE-ABS(catfish) OR TITLE-ABS(whale) OR TITLE-ABS(whales) OR TITLE-ABS(fox) OR TITLE-ABS(foxes) OR TITLE-ABS(ape) OR TITLE-ABS(apes) OR TITLE-ABS(elephant) OR TITLE-ABS(elephants) OR TITLE-ABS(bos) OR TITLE-ABS(marmoset) OR TITLE-ABS(marmosets) OR TITLE-ABS(cod) OR TITLE-ABS(cods) OR TITLE-ABS(shark) OR TITLE-ABS(sharks) OR TITLE-ABS(wolf) OR TITLE-ABS(eel) OR TITLE-ABS(eels) OR TITLE-ABS(auratus) OR TITLE-ABS(rattus) OR TITLE-ABS(zebra) OR TITLE-ABS(zebras) OR TITLE-ABS(tilapia) OR TITLE-ABS(tilapias) OR TITLE-ABS(gilt) OR TITLE-ABS(camel) OR TITLE-ABS(camels) OR TITLE-ABS(squid) OR TITLE-ABS(gallus) OR TITLE-ABS(marsupial) OR TITLE-ABS(marsupials) OR TITLE-ABS(vole) OR TITLE-ABS(voles) OR TITLE-ABS(fascicularis) OR TITLE-ABS(ovis) OR TITLE-ABS(salmonid) OR TITLE-ABS(salmonids) OR TITLE-ABS(tiger) OR TITLE-ABS(tigers) OR TITLE-ABS(dolphin) OR TITLE-ABS(dolphins) OR TITLE-ABS(robin) OR TITLE-ABS(robins) OR TITLE-ABS(carpio) OR TITLE-ABS(opossum) OR TITLE-ABS(opossums) OR TITLE-ABS(cyprinus) OR TITLE-ABS(salamander) OR TITLE-ABS(salamanders) OR TITLE-ABS(felis) OR TITLE-ABS(mink) OR TITLE-ABS(minks) OR TITLE-ABS(swan) OR TITLE-ABS(swans) OR TITLE-ABS(norvegicus) OR TITLE-ABS(bufo) OR TITLE-ABS(torpedo) OR TITLE-ABS(bass) OR TITLE-ABS(lamprey) OR TITLE-ABS(lampreys) OR TITLE-ABS(sus) OR TITLE-ABS(python) OR TITLE-ABS(pythons) OR TITLE-ABS(tetrapod) OR TITLE-ABS(tetrapods) OR TITLE-ABS(shrew) OR TITLE-ABS(shrews) OR TITLE-ABS(lion) OR TITLE-ABS(lions) OR TITLE-ABS(hog) OR TITLE-ABS(hogs) OR TITLE-ABS(songbird) OR TITLE-ABS(songbirds) OR TITLE-ABS(oreochromis) OR TITLE-ABS(starling) OR TITLE-ABS(starlings) OR TITLE-ABS(caprine) OR TITLE-ABS(carassius) OR TITLE-ABS(owl) OR TITLE-ABS(owls) OR TITLE-ABS(newt) OR TITLE-ABS(newts) OR TITLE-ABS(papio) OR TITLE-ABS(scrofa) OR TITLE-ABS(hare) OR TITLE-ABS(hares) OR TITLE-ABS(gorilla) OR TITLE-ABS(gorillas) OR TITLE-ABS(flounder) OR TITLE-ABS(flounders) OR TITLE-ABS(goose) OR TITLE-ABS(herring) OR TITLE-ABS(herrings) OR TITLE-ABS(therian) OR TITLE-ABS(buffaloes) OR TITLE-ABS(canary) OR TITLE-ABS(sparrow) OR TITLE-ABS(sparrows) OR TITLE-ABS(microtus) OR TITLE-ABS(octopus) OR TITLE-ABS(troglodytes) OR TITLE-ABS(tuna) OR TITLE-ABS(amphibia) OR TITLE-ABS(chinchilla) OR TITLE-ABS(chinchillas) OR TITLE-ABS(ide) OR TITLE-ABS(oryzias) OR TITLE-ABS(cervus) OR TITLE-ABS(kangaroo) OR TITLE-ABS(kangaroos) OR TITLE-ABS(armadillo) OR TITLE-ABS(armadillos) OR TITLE-ABS(callithrix) OR TITLE-ABS("pan troglodytes") OR TITLE-ABS(saimiri) OR TITLE-ABS(cichlid) OR TITLE-ABS(cichlids) OR TITLE-ABS(donkey) OR TITLE-ABS(donkeys) OR TITLE-ABS(bream) OR TITLE-ABS(char) OR TITLE-ABS(chars) OR TITLE-ABS(finch) OR TITLE-ABS(raccoon) OR TITLE-ABS(raccoons) OR TITLE-ABS(bothrops) OR TITLE-

		ABS(anguilla) OR TITLE-ABS(perch) OR TITLE-ABS(cricetus) OR TITLE-ABS(seabird) OR TITLE-ABS(seabirds) OR TITLE-ABS(buck) OR TITLE-ABS(bucks) OR TITLE-ABS(naja) OR TITLE-ABS(coturnix) OR TITLE-ABS(salmonids) OR TITLE-ABS(geese) OR TITLE-ABS(minnow) OR TITLE-ABS(minnows) OR TITLE-ABS(raptor) OR TITLE-ABS(raptors) OR TITLE-ABS(merione) OR TITLE-ABS(meriones) OR TITLE-ABS(rodentia) OR TITLE-ABS(elaphus) OR TITLE-ABS(amniote) OR TITLE-ABS(amniotes) OR TITLE-ABS(elasmobranch) OR TITLE-ABS(emu) OR TITLE-ABS(emus) OR TITLE-ABS(peromyscus) OR TITLE-ABS(hominid) OR TITLE-ABS(hominids) OR TITLE-ABS(bubalus) OR TITLE-ABS(crotalus) OR TITLE-ABS(gull) OR TITLE-ABS(gulls) OR TITLE-ABS(anas) OR TITLE-ABS(anura) OR TITLE-ABS(lemur) OR TITLE-ABS(lemurs) OR TITLE-ABS(crow) OR TITLE-ABS(crows) OR TITLE-ABS(camelus) OR TITLE-ABS(gibbon) OR TITLE-ABS(gibbons) OR TITLE-ABS(waterfowl) OR TITLE-ABS(parrot) OR TITLE-ABS(parrots) OR TITLE-ABS(eels) OR TITLE-ABS(cob) OR TITLE-ABS(stickleback) OR TITLE-ABS(sticklebacks) OR TITLE-ABS(columba) OR TITLE-ABS(mesocricetus) OR TITLE-ABS(ambystoma) OR TITLE-ABS(raven) OR TITLE-ABS(ravens) OR TITLE-ABS(gadus) OR TITLE-ABS(penguin) OR TITLE-ABS(penguins) OR TITLE-ABS(orangutan) OR TITLE-ABS(orangutans) OR TITLE-ABS(sturgeon) OR TITLE-ABS(sturgeons) OR TITLE-ABS(cuniculus) OR TITLE-ABS(aves) OR TITLE-ABS(virginianus) OR TITLE-ABS(cephalopod) OR TITLE-ABS(cephalopods) OR TITLE-ABS(cebus) OR TITLE-ABS(sparus) OR TITLE-ABS(tortoise) OR TITLE-ABS(tortoises) OR TITLE-ABS(guttata) OR TITLE-ABS(morhua) OR TITLE-ABS(unguiculatus) OR TITLE-ABS(dogfish) OR TITLE-ABS(vulpes) OR TITLE-ABS(mallard) OR TITLE-ABS(mallards) OR TITLE-ABS(apodemus) OR TITLE-ABS(alligator) OR TITLE-ABS(alligators) OR TITLE-ABS(oryctolagus) OR TITLE-ABS(llama) OR TITLE-ABS(llamas) OR TITLE-ABS(reindeer) OR TITLE-ABS(mustela) OR TITLE-ABS(duckling) OR TITLE-ABS(ducklings) OR TITLE-ABS(wolves) OR TITLE-ABS(sander) OR TITLE-ABS(amazona) OR TITLE-ABS(zebu) OR TITLE-ABS(badger) OR TITLE-ABS(badgers) OR TITLE-ABS(dove) OR TITLE-ABS(doves) OR TITLE-ABS(ictalurus) OR TITLE-ABS(capra) OR TITLE-ABS(capras) OR TITLE-ABS(equus) OR TITLE-ABS(camelid) OR TITLE-ABS(camelids) OR TITLE-ABS(poecilia) OR TITLE-ABS(mule) OR TITLE-ABS(mules) OR TITLE-ABS(perciformes) OR TITLE-ABS(salvelinus) OR TITLE-ABS(labrax) OR TITLE-ABS(cyprinidae) OR TITLE-ABS(ariidae) OR TITLE-ABS(crocodile) OR TITLE-ABS(crocodiles) OR TITLE-ABS(fundulus) OR TITLE-ABS(dicentrarchus) OR TITLE-ABS(clarias) OR TITLE-ABS(cercopithecus) OR TITLE-ABS(chiroptera) OR TITLE-ABS(alpaca) OR TITLE-ABS(alpacas) OR TITLE-ABS(pike) OR TITLE-ABS(pikes) OR TITLE-ABS(paralichthys) OR TITLE-ABS(puma) OR TITLE-ABS(pumas) OR TITLE-ABS(didelphis) OR TITLE-ABS(pisces) OR TITLE-ABS(macropus) OR TITLE-ABS(triturus) OR TITLE-ABS(bison) OR TITLE-ABS(bisons) OR TITLE-ABS(epinephelus) OR TITLE-ABS(gasterosteus) OR TITLE-ABS(panthera) OR TITLE-ABS(acipenser) OR TITLE-ABS(mackerel) OR TITLE-ABS(mackerels) OR TITLE-ABS(tamarin) OR TITLE-ABS(tamarins) OR TITLE-ABS(ostrich) OR TITLE-ABS(anolis) OR TITLE-ABS(vervet) OR TITLE-ABS(vervets) OR TITLE-ABS(wallaby) OR TITLE-ABS(glareolus) OR TITLE-ABS(beaver) OR TITLE-ABS(beavers) OR TITLE-ABS(dromedary) OR TITLE-ABS(catus) OR TITLE-ABS(killifish) OR TITLE-ABS(pimephales) OR TITLE-ABS(promelas) OR TITLE-ABS(aotus) OR TITLE-ABS(phoca) OR TITLE-ABS(panda) OR TITLE-ABS(pandas) OR TITLE-ABS(porpoise) OR TITLE-ABS(porpoises) OR TITLE-ABS(myotis) OR TITLE-ABS(yak) OR TITLE-ABS(yaks) OR TITLE-ABS(agkistrodon) OR TITLE-ABS(vipera) OR TITLE-ABS(otter) OR TITLE-ABS(otters) OR TITLE-ABS(turbot) OR TITLE-ABS(turbots) OR TITLE-ABS(squamate) OR TITLE-ABS(carnivora) OR TITLE-ABS(mullet) OR TITLE-ABS(mullets) OR TITLE-ABS(hawk) OR TITLE-ABS(hawks) OR TITLE-ABS(taeniopygia) OR TITLE-ABS(seahorse) OR TITLE-ABS(seahorses) OR TITLE-ABS("poecilia reticulata") OR TITLE-ABS(falcon) OR TITLE-ABS(falcons) OR TITLE-ABS(prosimian) OR TITLE-ABS(prosimians) OR TITLE-ABS(parus) OR TITLE-ABS(perca) OR TITLE-ABS(fingerling) OR TITLE-ABS(fingerlings) OR TITLE-ABS(antelope) OR TITLE-ABS(antelopes) OR TITLE-ABS(tupaia) OR TITLE-ABS(passeriformes) OR TITLE-ABS(sepia) OR TITLE-ABS(saguinus) OR TITLE-ABS(coyote) OR TITLE-ABS(coyotes) OR TITLE-ABS(pongo) OR TITLE-ABS(meleagris) OR TITLE-ABS(reptilia) OR TITLE-ABS(lepus) OR TITLE-ABS(psittacine) OR TITLE-ABS(hagfish) OR TITLE-ABS(warbler) OR TITLE-ABS(warblers) OR TITLE-ABS("russell's viper") OR TITLE-ABS("russell's vipers") OR TITLE-ABS(smolt) OR TITLE-ABS(smolts) OR TITLE-ABS(budgerigar) OR TITLE-ABS(sardine) OR TITLE-ABS(sardines) OR TITLE-ABS(cavia) OR TITLE-ABS(cavias) OR TITLE-ABS(hyla) OR TITLE-ABS(pleurodeles) OR TITLE-ABS(siluriformes) OR TITLE-ABS("great tit") OR TITLE-ABS("great tits") OR TITLE-ABS(guppy) OR TITLE-ABS(bonobo) OR TITLE-ABS(bonobos) OR TITLE-ABS(rutilus) OR TITLE-ABS(trichosurus) OR TITLE-ABS(muridae) OR TITLE-ABS(phodopus) OR TITLE-ABS(channa) OR TITLE-ABS(squalus) OR TITLE-ABS(lynx) OR TITLE-ABS(sturnus) OR TITLE-ABS(petromyzon) OR TITLE-ABS(vitulina) OR TITLE-ABS(monodelphis) OR TITLE-ABS(cuttlefish) OR TITLE-ABS(adder) OR TITLE-ABS(adders) OR TITLE-ABS(lepomis) OR TITLE-ABS(canaria) OR TITLE-ABS(gambusia) OR TITLE-ABS(guppies) OR TITLE-ABS(xiphophorus) OR TITLE-ABS(flatfish) OR TITLE-ABS(koala) OR TITLE-ABS(koalas) OR TITLE-ABS(labeo) OR TITLE-ABS(stingray) OR TITLE-ABS(stingrays) OR TITLE-ABS(chelonia) OR TITLE-ABS(lampetra) OR TITLE-ABS(spermophilus) OR TITLE-ABS(crocodilian) OR TITLE-ABS("passer domesticus") OR TITLE-ABS(sciurus) OR TITLE-ABS(artiodactyla) OR TITLE-ABS(ranidae) OR TITLE-ABS(corvus) OR TITLE-ABS(necturus) OR TITLE-ABS(platypus) OR TITLE-ABS(canaries) OR TITLE-ABS(bovid) OR TITLE-ABS(lagopus) OR TITLE-ABS(trimeresurus) OR TITLE-ABS(gariepinus) OR TITLE-ABS(marten) OR TITLE-ABS(martens) OR TITLE-ABS(drosophilidae) OR TITLE-ABS(mugil) OR TITLE-ABS(sunfish) OR TITLE-ABS(porcellus) OR TITLE-ABS(cypriniformes) OR TITLE-ABS(alouatta) OR TITLE-ABS(scophthalmus) OR TITLE-ABS(anser) OR TITLE-ABS(electrophorus) OR TITLE-ABS(putorius) OR TITLE-ABS(iguana) OR TITLE-ABS(iguanas) OR TITLE-ABS(lama) OR TITLE-ABS(lamas) OR TITLE-ABS(takifugu) OR TITLE-ABS(circus) OR TITLE-ABS(eptesicus) OR TITLE-ABS(flycatcher) OR TITLE-ABS(galago) OR TITLE-ABS(galagos) OR TITLE-ABS(trachemys) OR TITLE-ABS(lungfish) OR TITLE-ABS(characiformes) OR TITLE-ABS(shorebird) OR TITLE-ABS(shorebirds) OR TITLE-ABS(giraffe) OR TITLE-ABS(giraffes) OR TITLE-ABS(micropterus) OR TITLE-ABS(scyliorhinus) OR TITLE-ABS(cichlidae) OR TITLE-ABS(loligo) OR TITLE-ABS(porcupine) OR TITLE-ABS(porcupines) OR TITLE-ABS(chub) OR TITLE-ABS(chubs) OR TITLE-ABS(solea) OR TITLE-ABS(pleuronectes) OR TITLE-ABS(hylidae) OR TITLE-ABS(viperidae) OR TITLE-ABS(echis) OR TITLE-ABS(sorex) OR TITLE-ABS(anchovy) OR TITLE-ABS(lagomorph) OR TITLE-ABS(ostriches) OR TITLE-ABS(vulture) OR TITLE-ABS(vultures) OR TITLE-ABS(whitefish) OR TITLE-ABS(araneus) OR TITLE-ABS(jird) OR TITLE-ABS(jirds) OR TITLE-ABS(tern) OR TITLE-ABS(esox) OR TITLE-ABS(drake) OR TITLE-ABS(drakes) OR TITLE-ABS(elapidae) OR TITLE-ABS(gallopavo) OR TITLE-ABS(chordata) OR TITLE-ABS(myodes) OR TITLE-ABS(caretta) OR TITLE-ABS(serinus) OR TITLE-ABS(grouse) OR TITLE-ABS(misgurnus) OR TITLE-ABS(meles) OR TITLE-ABS(blackbird) OR TITLE-ABS(blackbirds) OR TITLE-ABS(coregonus) OR TITLE-ABS(bobwhite) OR TITLE-ABS(bobwhites) OR TITLE-ABS(heteropneustes) OR TITLE-ABS(mammoth) OR TITLE-ABS(mammoths) OR TITLE-ABS(turdus) OR TITLE-ABS(rhinella) OR TITLE-ABS(ateles) OR TITLE-ABS(characidae) OR TITLE-ABS(clupea) OR TITLE-ABS(bungarus) OR TITLE-ABS(brill) OR TITLE-ABS("struthio camelus") OR TITLE-ABS(sloth) OR TITLE-ABS(sloths) OR TITLE-ABS(pteropus) OR TITLE-ABS(sculpin) OR TITLE-ABS(anthropoids) OR TITLE-ABS(pollock) OR TITLE-ABS(pollocks) OR TITLE-ABS(morone) OR TITLE-ABS("pan paniscus") OR TITLE-ABS(litoria) OR TITLE-ABS(chipmunk) OR TITLE-ABS(chipmunks) OR TITLE-ABS(balaenoptera) OR TITLE-ABS(marmota) OR TITLE-ABS(melopsittacus) OR TITLE-ABS(hyrax) OR TITLE-ABS(lemming) OR TITLE-ABS(lemmings) OR TITLE-ABS(halibut) OR TITLE-ABS(hylobates) OR TITLE-ABS(lates) OR TITLE-ABS(caiman) OR TITLE-ABS(caimans) OR TITLE-ABS(sigmodon) OR TITLE-ABS(stenella) OR TITLE-ABS(barbel) OR TITLE-ABS(barbels) OR TITLE-ABS(sterna) OR TITLE-ABS(parakeet) OR TITLE-ABS(parakeets) OR TITLE-ABS(phocoena) OR TITLE-ABS(leptodactylus) OR TITLE-ABS(canidae) OR TITLE-ABS(buteo) OR TITLE-ABS(harengus) OR TITLE-ABS(gopher) OR TITLE-ABS(gophers) OR TITLE-ABS(marmot) OR TITLE-ABS(marmots) OR TITLE-ABS(gosling) OR TITLE-ABS(goslings) OR TITLE-ABS(platichthys) OR TITLE-ABS(gar) OR TITLE-ABS(gars) OR TITLE-ABS(sebastes) OR TITLE-ABS(marsupialia) OR TITLE-ABS(notophthalmus) OR TITLE-ABS(gazelle) OR TITLE-ABS(gazelles) OR TITLE-ABS(insectivora) OR TITLE-ABS(paridae) OR TITLE-ABS(felidae) OR TITLE-ABS(russula) OR TITLE-ABS(galliformes) OR TITLE-ABS(bombina) OR TITLE-ABS(colobus) OR TITLE-ABS(echidna) OR TITLE-ABS(echidnas) OR TITLE-ABS(seabass) OR TITLE-

		ABS(syncerus) OR TITLE-ABS(plaice) OR TITLE-ABS("blue tit") OR TITLE-ABS("blue tits") OR TITLE-ABS(pagrus) OR TITLE-ABS(catfishes) OR TITLE-ABS(cetacea) OR TITLE-ABS(barbus) OR TITLE-ABS(cygnus) OR TITLE-ABS(ficedula) OR TITLE-ABS(chamois) OR TITLE-ABS(colubridae) OR TITLE-ABS(perches) OR TITLE-ABS(coelacanth) OR TITLE-ABS(fitch) OR TITLE-ABS(urodela) OR TITLE-ABS(cynops) OR TITLE-ABS(martes) OR TITLE-ABS(halichoerus) OR TITLE-ABS(aix) OR TITLE-ABS(salmonidae) OR TITLE-ABS(leuciscus) OR TITLE-ABS(magpie) OR TITLE-ABS(magpies) OR TITLE-ABS(silurus) OR TITLE-ABS(whiting) OR TITLE-ABS(whitings) OR TITLE-ABS(anseriformes) OR TITLE-ABS(colinus) OR TITLE-ABS(rhea) OR TITLE-ABS(chlorocebus) OR TITLE-ABS(octodon) OR TITLE-ABS(acinonyx) OR TITLE-ABS(mouflon) OR TITLE-ABS(mouflons) OR TITLE-ABS(ibex) OR TITLE-ABS(tetraodon) OR TITLE-ABS(bufonidae) OR TITLE-ABS(equidae) OR TITLE-ABS(jackal) OR TITLE-ABS(cephalopoda) OR TITLE-ABS(dendroaspis) OR TITLE-ABS(glama) OR TITLE-ABS(muskrat) OR TITLE-ABS(muskrats) OR TITLE-ABS(sable) OR TITLE-ABS(sables) OR TITLE-ABS(wildebeest) OR TITLE-ABS(streptopelia) OR TITLE-ABS(albifrons) OR TITLE-ABS(vespertilionidae) OR TITLE-ABS(woodpecker) OR TITLE-ABS(woodpeckers) OR TITLE-ABS(muntjac) OR TITLE-ABS(muntjacs) OR TITLE-ABS(archosaur) OR TITLE-ABS(branta) OR TITLE-ABS(cricetulus) OR TITLE-ABS(megalobrama) OR TITLE-ABS(poeciliidae) OR TITLE-ABS(desmodus) OR TITLE-ABS(snakehead) OR TITLE-ABS(snakeheads) OR TITLE-ABS(tench) OR TITLE-ABS(teal) OR TITLE-ABS(teals) OR TITLE-ABS(bandicoot) OR TITLE-ABS(bandicoots) OR TITLE-ABS(apteronotus) OR TITLE-ABS(phyllostomidae) OR TITLE-ABS(crocidura) OR TITLE-ABS(buzzard) OR TITLE-ABS(buzzards) OR TITLE-ABS(larimichthys) OR TITLE-ABS(cercocebus) OR TITLE-ABS(pipistrellus) OR TITLE-ABS(erithacus) OR TITLE-ABS(impala) OR TITLE-ABS(impalas) OR TITLE-ABS(rousettus) OR TITLE-ABS(haddock) OR TITLE-ABS(haddocks) OR TITLE-ABS(tinca) OR TITLE-ABS(ratite) OR TITLE-ABS(calidris) OR TITLE-ABS(cynoglossus) OR TITLE-ABS(hypophthalmichthys) OR TITLE-ABS(bullock) OR TITLE-ABS(bullocks) OR TITLE-ABS(dromedaries) OR TITLE-ABS(alectoris) OR TITLE-ABS(filly) OR TITLE-ABS(salamandra) OR TITLE-ABS(cingulata) OR TITLE-ABS(bitis) OR TITLE-ABS(grus) OR TITLE-ABS(ammodytes) OR TITLE-ABS(macaw) OR TITLE-ABS(macaws) OR TITLE-ABS(hypoleuca) OR TITLE-ABS(sapajus) OR TITLE-ABS(cyprinodontiformes) OR TITLE-ABS(hippopotamus) OR TITLE-ABS(pelophylax) OR TITLE-ABS(capybara) OR TITLE-ABS(capybaras) OR TITLE-ABS(weasel) OR TITLE-ABS(weasels) OR TITLE-ABS(cairina) OR TITLE-ABS(cynomys) OR TITLE-ABS(lutra) OR TITLE-ABS(cockatoo) OR TITLE-ABS(cockatoos) OR TITLE-ABS(lachesis) OR TITLE-ABS(lagomorpha) OR TITLE-ABS(rupicapra) OR TITLE-ABS(daboia) OR TITLE-ABS("orang utan") OR TITLE-ABS("orang utans") OR TITLE-ABS(platyrrhini) OR TITLE-ABS(charadriiformes) OR TITLE-ABS(micrurus) OR TITLE-ABS(psittaciformes) OR TITLE-ABS(spalax) OR TITLE-ABS(loris) OR TITLE-ABS(mustelidae) OR TITLE-ABS(sylvilagus) OR TITLE-ABS(vitticeps) OR TITLE-ABS(cockatiel) OR TITLE-ABS(mustelus) OR TITLE-ABS(cottus) OR TITLE-ABS(erythrocebus) OR TITLE-ABS(dipodomys) OR TITLE-ABS(platessa) OR TITLE-ABS(callicebus) OR TITLE-ABS(loricariidae) OR TITLE-ABS(catostomus) OR TITLE-ABS(cuneata) OR TITLE-ABS(cyanistes) OR TITLE-ABS(cyprinodon) OR TITLE-ABS(sigmodontinae) OR TITLE-ABS(elasmobranchii) OR TITLE-ABS(trichechus) OR TITLE-ABS(sauropsid) OR TITLE-ABS(xenarthra) OR TITLE-ABS(dormouse) OR TITLE-ABS(perissodactyla) OR TITLE-ABS(nautilus) OR TITLE-ABS(cirrhinus) OR TITLE-ABS(gulo) OR TITLE-ABS(tragelaphus) OR TITLE-ABS(merula) OR TITLE-ABS(numida) OR TITLE-ABS(sciaenidae) OR TITLE-ABS(cerastes) OR TITLE-ABS(sciuridae) OR TITLE-ABS(gibbosus) OR TITLE-ABS(octopuses) OR TITLE-ABS(eland) OR TITLE-ABS(elands) OR TITLE-ABS(phyllomedusa) OR TITLE-ABS(pogona) OR TITLE-ABS(walrus) OR TITLE-ABS(agamidae) OR TITLE-ABS(leptodactylidae) OR TITLE-ABS(ridibundus) OR TITLE-ABS(leontopithecus) OR TITLE-ABS(anteater) OR TITLE-ABS(anteaters) OR TITLE-ABS(pelodiscus) OR TITLE-ABS(cebidae) OR TITLE-ABS(columbianus) OR TITLE-ABS("pelteobagrus fulvidraco") OR TITLE-ABS(hominoidea) OR TITLE-ABS(mandrillus) OR TITLE-ABS("zonotrichia leucophrys") OR TITLE-ABS(agama) OR TITLE-ABS(gobiocypris) OR TITLE-ABS("bearded dragon") OR TITLE-ABS("bearded dragons") OR TITLE-ABS(sarotherodon) OR TITLE-ABS(talpa) OR TITLE-ABS(discoglossus) OR TITLE-ABS(hagfishes) OR TITLE-ABS(sphenodon) OR TITLE-ABS(gudgeon) OR TITLE-ABS(amphiuma) OR TITLE-ABS(aythya) OR TITLE-ABS(tenrec) OR TITLE-ABS(tenrec) OR TITLE-ABS(hominidae) OR TITLE-ABS(risoria) OR TITLE-ABS(salamandridae) OR TITLE-ABS(camelidae) OR TITLE-ABS(columbiformes) OR TITLE-ABS(latimeria) OR TITLE-ABS(plover) OR TITLE-ABS(plovers) OR TITLE-ABS(afrotheria) OR TITLE-ABS("falco sparverius") OR TITLE-ABS(polecat) OR TITLE-ABS(polecats) OR TITLE-ABS(crotalinae) OR TITLE-ABS(salvadora) OR TITLE-ABS(tarsier) OR TITLE-ABS(lucioperca) OR TITLE-ABS(anchovies) OR TITLE-ABS(lungfishes) OR TITLE-ABS(terrapin) OR TITLE-ABS("dromaius novaehollandiae") OR TITLE-ABS(lateolabrax) OR TITLE-ABS(eigenmannia) OR TITLE-ABS(pelamis) OR TITLE-ABS(theropithecus) OR TITLE-ABS(murinae) OR TITLE-ABS(gander) OR TITLE-ABS(gymnotus) OR TITLE-ABS(pseudacris) OR TITLE-ABS(gymnophiona) OR TITLE-ABS(gymnotiformes) OR TITLE-ABS(laticauda) OR TITLE-ABS(falconiformes) OR TITLE-ABS(dugong) OR TITLE-ABS(dugongs) OR TITLE-ABS(pintail) OR TITLE-ABS(pintails) OR TITLE-ABS(rook) OR TITLE-ABS(rooks) OR TITLE-ABS(lasiurus) OR TITLE-ABS(catshark) OR TITLE-ABS(catsharks) OR TITLE-ABS(micropogonias) OR TITLE-ABS("red junglefowl") OR TITLE-ABS(paddlefish) OR TITLE-ABS(ophiophagus) OR TITLE-ABS(hollandicus) OR TITLE-ABS(nymphicus) OR TITLE-ABS(pimelodidae) OR TITLE-ABS(aepyceros) OR TITLE-ABS(cobitidae) OR TITLE-ABS(strigiformes) OR TITLE-ABS(cobitis) OR TITLE-ABS(dormice) OR TITLE-ABS(alytes) OR TITLE-ABS(calloselasma) OR TITLE-ABS(guanaco) OR TITLE-ABS(phasianidae) OR TITLE-ABS("round goby") OR TITLE-ABS(trichogaster) OR TITLE-ABS(catarrhini) OR TITLE-ABS(eelpout) OR TITLE-ABS(eelpouts) OR TITLE-ABS(galaxias) OR TITLE-ABS(gaur) OR TITLE-ABS(pungitius) OR TITLE-ABS(suslik) OR TITLE-ABS(susliks) OR TITLE-ABS(flatfishes) OR TITLE-ABS(percidae) OR TITLE-ABS(caprinae) OR TITLE-ABS(todarodes) OR TITLE-ABS(osmerus) OR TITLE-ABS(ameiurus) OR TITLE-ABS(anthropoidea) OR TITLE-ABS("castor canadensis") OR TITLE-ABS(pouting) OR TITLE-ABS(poutings) OR TITLE-ABS(tetraodontiformes) OR TITLE-ABS(arvicolinae) OR TITLE-ABS(siamang) OR TITLE-ABS(siamangs) OR TITLE-ABS("castor fiber") OR TITLE-ABS(nomascus) OR TITLE-ABS("red knot") OR TITLE-ABS("red knots") OR TITLE-ABS(syngnathidae) OR TITLE-ABS(iguanidae) OR TITLE-ABS(eretmochelys) OR TITLE-ABS(ursidae) OR TITLE-ABS(callimico) OR TITLE-ABS(columbidae) OR TITLE-ABS(microhylidae) OR TITLE-ABS(anaxyrus) OR TITLE-ABS(menidia) OR TITLE-ABS(pipistrelle) OR TITLE-ABS(greylag) OR TITLE-ABS(pipidae) OR TITLE-ABS(scandentia) OR TITLE-ABS(bowfin) OR TITLE-ABS(bowfins) OR TITLE-ABS(dendrobatidae) OR TITLE-ABS(zenaida) OR TITLE-ABS(bushbaby) OR TITLE-ABS(harrier) OR TITLE-ABS(harriers) OR TITLE-ABS(macropodidae) OR TITLE-ABS(pygerythrus) OR TITLE-ABS(clupeidae) OR TITLE-ABS(odorrana) OR TITLE-ABS(corvidae) OR TITLE-ABS(jerboa) OR TITLE-ABS(jerboas) OR TITLE-ABS(canutus) OR TITLE-ABS(hylobatidae) OR TITLE-ABS(clupeiformes) OR TITLE-ABS("great cormorant") OR TITLE-ABS("great cormorants") OR TITLE-ABS(scorpaeniformes) OR TITLE-ABS(chondrostean) OR TITLE-ABS(garfish) OR TITLE-ABS(proboscidea) OR TITLE-ABS(psetta) OR TITLE-ABS(diapsid) OR TITLE-ABS(serotinus) OR TITLE-ABS(tetrao) OR TITLE-ABS(walruses) OR TITLE-ABS(carcharhiniformes) OR TITLE-ABS(leucoraja) OR TITLE-ABS(pumpkinseed) OR TITLE-ABS(dosidicus) OR TITLE-ABS(acipenseriformes) OR TITLE-ABS(daubentonii) OR TITLE-ABS(emberizidae) OR TITLE-ABS(gadiformes) OR TITLE-ABS(hyraxes) OR TITLE-ABS(stizostedion) OR TITLE-ABS(wolverine) OR TITLE-ABS(wolverines) OR TITLE-ABS(lissotriton) OR TITLE-ABS(acanthurus) OR TITLE-ABS(centrarchidae) OR TITLE-ABS(gloydius) OR TITLE-ABS(laurasiatheria) OR TITLE-ABS(limosa) OR TITLE-ABS(psittacula) OR TITLE-ABS(leporidae) OR TITLE-ABS(proteidae) OR TITLE-ABS(zander) OR TITLE-ABS(zanders) OR TITLE-ABS(arapaima) OR TITLE-ABS(bagridae) OR TITLE-ABS(cyprinodontidae) OR TITLE-ABS(mithun) OR TITLE-ABS(pandion) OR TITLE-ABS(jackdaw) OR TITLE-ABS(jackdaws) OR TITLE-ABS(procyonidae) OR TITLE-ABS(carus) OR TITLE-ABS(jaculus) OR TITLE-ABS(salmoniformes) OR TITLE-ABS("common sole") OR TITLE-ABS("common soles") OR TITLE-ABS(protobothrops) OR TITLE-ABS(calamita) OR TITLE-ABS(brachyteles) OR TITLE-ABS(trionyx) OR TITLE-ABS(turdidae) OR TITLE-ABS(boidae) OR TITLE-ABS(luscinia) OR TITLE-ABS(pugnax) OR TITLE-ABS(euarchontoglires) OR TITLE-ABS(saithe) OR TITLE-ABS(saithes) OR TITLE-ABS(symphalangus) OR TITLE-ABS(aardvark) OR TITLE-ABS(aardvarks) OR TITLE-ABS(oystercatcher) OR TITLE-ABS(oystercatchers) OR TITLE-ABS(arius) OR TITLE-

		ABS(corydoras) OR TITLE-ABS(poacher) OR TITLE-ABS(poachers) OR TITLE-ABS(aurochs) OR TITLE-ABS(cebuella) OR TITLE-ABS(crecca) OR TITLE-ABS(lemuridae) OR TITLE-ABS(sirenia) OR TITLE-ABS(lemmus) OR TITLE-ABS(perdix) OR TITLE-ABS(glires) OR TITLE-ABS(lepidosaur) OR TITLE-ABS(muskox) OR TITLE-ABS(deinagkistrodon) OR TITLE-ABS(pholidota) OR TITLE-ABS(holocephali) OR TITLE-ABS(cercopithecinae) OR TITLE-ABS(clariidae) OR TITLE-ABS(agapornis) OR TITLE-ABS(doryteuthis) OR TITLE-ABS(tyrannidae) OR TITLE-ABS(dicroglossidae) OR TITLE-ABS(godwit) OR TITLE-ABS(godwits) OR TITLE-ABS(monedula) OR TITLE-ABS(pongidae) OR TITLE-ABS(atheriniformes) OR TITLE-ABS(colobinae) OR TITLE-ABS(lophocebus) OR TITLE-ABS(atelidae) OR TITLE-ABS(cottidae) OR TITLE-ABS(leucopsis) OR TITLE-ABS(acanthuridae) OR TITLE-ABS(didelphimorphia) OR TITLE-ABS(elver) OR TITLE-ABS(elvers) OR TITLE-ABS(lapponica) OR TITLE-ABS(dermoptera) OR TITLE-ABS("european hake") OR TITLE-ABS("european hakes") OR TITLE-ABS(gerbillinae) OR TITLE-ABS(banteng) OR TITLE-ABS(hartebeest) OR TITLE-ABS(hartebeests) OR TITLE-ABS(hogget) OR TITLE-ABS(haematopus) OR TITLE-ABS("anguis fragilis") OR TITLE-ABS("grey heron") OR TITLE-ABS("grey herons") OR TITLE-ABS("blue whiting") OR TITLE-ABS("blue whitings") OR TITLE-ABS(furnariidae) OR TITLE-ABS(macrovipera) OR TITLE-ABS(esocidae) OR TITLE-ABS(lapwing) OR TITLE-ABS(lapwings) OR TITLE-ABS(mylopharyngodon) OR TITLE-ABS(wallabia) OR TITLE-ABS(beloniformes) OR TITLE-ABS(potoroo) OR TITLE-ABS(potoroos) OR TITLE-ABS("athene noctua") OR TITLE-ABS(pleuronectidae) OR TITLE-ABS(bushbabies) OR TITLE-ABS(muscicapidae) OR TITLE-ABS(alligatoridae) OR TITLE-ABS(fuligula) OR TITLE-ABS("bush baby") OR TITLE-ABS(guineafowl) OR TITLE-ABS(spoonbill) OR TITLE-ABS(spoonbills) OR TITLE-ABS(viverridae) OR TITLE-ABS(catostomidae) OR TITLE-ABS(zebrafishes) OR TITLE-ABS(ibexes) OR TITLE-ABS(vendace) OR TITLE-ABS(estrildidae) OR TITLE-ABS(monotremata) OR TITLE-ABS(sepiella) OR TITLE-ABS(ambystomatidae) OR TITLE-ABS(shelduck) OR TITLE-ABS(shelducks) OR TITLE-ABS(treeshrew) OR TITLE-ABS(treeshrews) OR TITLE-ABS(hoplobatrachus) OR TITLE-ABS(pochard) OR TITLE-ABS(hoolock) OR TITLE-ABS(hoolocks) OR TITLE-ABS(lynxes) OR TITLE-ABS(antilope) OR TITLE-ABS(antilopes) OR TITLE-ABS(blackbuck) OR TITLE-ABS(blackbucks) OR TITLE-ABS(cricetinae) OR TITLE-ABS(paramisgurnus) OR TITLE-ABS(skylark) OR TITLE-ABS(skylarks) OR TITLE-ABS(soleidae) OR TITLE-ABS(allobates) OR TITLE-ABS("northern wheatear") OR TITLE-ABS("northern wheatears") OR TITLE-ABS(pitheciidae) OR TITLE-ABS(takin) OR TITLE-ABS(theria) OR TITLE-ABS(vanellus) OR TITLE-ABS(galaxiidae) OR TITLE-ABS(lorisidae) OR TITLE-ABS(ostralegus) OR TITLE-ABS(palaeognathae) OR TITLE-ABS("stone loach") OR TITLE-ABS(alauda) OR TITLE-ABS(callitrichinae) OR TITLE-ABS(caniformia) OR TITLE-ABS(duttaphrynus) OR TITLE-ABS(ictaluridae) OR TITLE-ABS(osteoglossiformes) OR TITLE-ABS(poultries) OR TITLE-ABS(curema) OR TITLE-ABS("ruddy turnstone") OR TITLE-ABS("ruddy turnstones") OR TITLE-ABS(sheatfish) OR TITLE-ABS(sunfishes) OR TITLE-ABS(centropomidae) OR TITLE-ABS(hemachatus) OR TITLE-ABS(platalea) OR TITLE-ABS(thamnophilidae) OR TITLE-ABS("song thrush") OR TITLE-ABS(atherinopsidae) OR TITLE-ABS(siluridae) OR TITLE-ABS(tadorna) OR TITLE-ABS(chroicocephalus) OR TITLE-ABS(ermine) OR TITLE-ABS(ermines) OR TITLE-ABS(gavialis) OR TITLE-ABS(ruff) OR TITLE-ABS(tupaiidae) OR TITLE-ABS(diprotodontia) OR TITLE-ABS(hyaenidae) OR TITLE-ABS(antilopinae) OR TITLE-ABS(crocodylidae) OR TITLE-ABS(herpestidae) OR TITLE-ABS(hippopotamidae) OR TITLE-ABS("northern shoveler") OR TITLE-ABS("round gobies") OR TITLE-ABS(cheirogaleidae) OR TITLE-ABS(indriidae) OR TITLE-ABS(fundulidae) OR TITLE-ABS(pythonidae) OR TITLE-ABS(rhynchocephalia) OR TITLE-ABS(anodorhynchus) OR TITLE-ABS("red-backed shrike") OR TITLE-ABS("red-backed shrikes") OR TITLE-ABS(triakidae) OR TITLE-ABS(phalangeridae) OR TITLE-ABS(aoudad) OR TITLE-ABS(boreoeutheria) OR TITLE-ABS("eurasian jay") OR TITLE-ABS("eurasian jays") OR TITLE-ABS(feliformia) OR TITLE-ABS(haplorhini) OR TITLE-ABS(osteoglossidae) OR TITLE-ABS(paenungulata) OR TITLE-ABS(struthioniformes) OR TITLE-ABS(ferina) OR TITLE-ABS(sanderling) OR TITLE-ABS(sanderlings) OR TITLE-ABS(spheniscidae) OR TITLE-ABS(cuttlefishes) OR TITLE-ABS(cygnet) OR TITLE-ABS(dasycneme) OR TITLE-ABS(gadwall) OR TITLE-ABS(gadwalls) OR TITLE-ABS("pelobates fuscus") OR TITLE-ABS(wryneck) OR TITLE-ABS(wrynecks) OR TITLE-ABS(afrosoricida) OR TITLE-ABS(culaea) OR TITLE-ABS("dover sole") OR TITLE-ABS("dover soles") OR TITLE-ABS(paralichthyidae) OR TITLE-ABS(passeridae) OR TITLE-ABS(osteolaemus) OR TITLE-ABS("song thrushes") OR TITLE-ABS(bluethroat) OR TITLE-ABS(bluethroats) OR TITLE-ABS(hydrophiidae) OR TITLE-ABS(megrim) OR TITLE-ABS(mephitidae) OR TITLE-ABS(strepsirhini) OR TITLE-ABS(tomistoma) OR TITLE-ABS(epidalea) OR TITLE-ABS(osmeriformes) OR TITLE-ABS("bush babies") OR TITLE-ABS(tarsiiform) OR TITLE-ABS(atelinae) OR TITLE-ABS(bufotes) OR TITLE-ABS("eurasian coot") OR TITLE-ABS("eurasian coots") OR TITLE-ABS(galagidae) OR TITLE-ABS(geopelia) OR TITLE-ABS(philomachus) OR TITLE-ABS(tubulidentata) OR TITLE-ABS(bombinatoridae) OR TITLE-ABS(pelobatidae) OR TITLE-ABS(tachysurus) OR TITLE-ABS(ailuridae) OR TITLE-ABS(woodlark) OR TITLE-ABS(woodlarks) OR TITLE-ABS(alcelaphinae) OR TITLE-ABS(redshank) OR TITLE-ABS(redshanks) OR TITLE-ABS(salientia) OR TITLE-ABS("sand smelt") OR TITLE-ABS("sand smelts") OR TITLE-ABS(woodmice) OR TITLE-ABS(woodmouse) OR TITLE-ABS(dasyproctidae) OR TITLE-ABS("eurasian wigeon") OR TITLE-ABS("eurasian wigeons") OR TITLE-ABS(garganey) OR TITLE-ABS(garganeys) OR TITLE-ABS("lemon sole") OR TITLE-ABS("lemon soles") OR TITLE-ABS("common dab") OR TITLE-ABS("common dabs") OR TITLE-ABS(graylag) OR TITLE-ABS(graylags) OR TITLE-ABS(leucorodia) OR TITLE-ABS(osphronemidae) OR TITLE-ABS(bewickii) OR TITLE-ABS("common moorhen") OR TITLE-ABS("common moorhens") OR TITLE-ABS(decapodiformes) OR TITLE-ABS(gobbler) OR TITLE-ABS(gobblers) OR TITLE-ABS(odontophoridae) OR TITLE-ABS(paddlefishes) OR TITLE-ABS(eutheria) OR TITLE-ABS(salmonine) OR TITLE-ABS(esociformes) OR TITLE-ABS("eurasian woodcock") OR TITLE-ABS("eurasian woodcocks") OR TITLE-ABS("european smelt") OR TITLE-ABS("european smelts") OR TITLE-ABS(goldfishes) OR TITLE-ABS(tenches) OR TITLE-ABS(tyranni) OR TITLE-ABS("common chaffinch") OR TITLE-ABS("common chaffinchs") OR TITLE-ABS("common redstart") OR TITLE-ABS("common redstarts") OR TITLE-ABS("common roach") OR TITLE-ABS("common roachs") OR TITLE-ABS("great knot") OR TITLE-ABS("great knots") OR TITLE-ABS(potoroidae) OR TITLE-ABS(alytidae) OR TITLE-ABS(coregonine) OR TITLE-ABS(dipteral) OR TITLE-ABS(leveret) OR TITLE-ABS("poeciliopsis gracilis") OR TITLE-ABS(amphiumidae) OR TITLE-ABS(batrachoidiformes) OR TITLE-ABS("bighead goby") OR TITLE-ABS(heteropneustidae) OR TITLE-ABS(lullula) OR TITLE-ABS("norway pout") OR TITLE-ABS("norway pouts") OR TITLE-ABS(sipunculida) OR TITLE-ABS(dogfishes) OR TITLE-ABS(sebastidae) OR TITLE-ABS(tarsiidae) OR TITLE-ABS(alethinophidia) OR TITLE-ABS("common nase") OR TITLE-ABS("common nases") OR TITLE-ABS("common sandpiper") OR TITLE-ABS("common sandpipers") OR TITLE-ABS("eurasian blackcap") OR TITLE-ABS("eurasian blackcaps") OR TITLE-ABS(pterocnemia) OR TITLE-ABS(syngnathiformes) OR TITLE-ABS("common chaffinches") OR TITLE-ABS(eupleridae) OR TITLE-ABS(octopodiformes) OR TITLE-ABS(phascolarctidae) OR TITLE-ABS(scophthalmidae) OR TITLE-ABS("starry smooth-hound") OR TITLE-ABS("starry smooth-hounds") OR TITLE-ABS(whitefishes) OR TITLE-ABS(cuniculidae) OR TITLE-ABS("european sprat") OR TITLE-ABS("european sprats") OR TITLE-ABS("rosy bitterling") OR TITLE-ABS("rosy bitterlings") OR TITLE-ABS("common dace") OR TITLE-ABS("common daces") OR TITLE-ABS("lesser weever") OR TITLE-ABS("lesser weevers") OR TITLE-ABS(scaldfish) OR TITLE-ABS("water rail") OR TITLE-ABS("water rails") OR TITLE-ABS(alouattinae) OR TITLE-ABS(centrarchiformes) OR TITLE-ABS("common whitethroat") OR TITLE-ABS("common whitethroats") OR TITLE-ABS(gavialidae) OR TITLE-ABS("grey gurnard") OR TITLE-ABS("grey gurnards") OR TITLE-ABS(lateolabracidae) OR TITLE-ABS(rheiformes) OR TITLE-ABS("tub gurnard") OR TITLE-ABS("tub gurnards") OR TITLE-ABS("common chiffchaff") OR TITLE-ABS("common chiffchaffs") OR TITLE-ABS(garfishes) OR TITLE-ABS("lesser whitethroat") OR TITLE-ABS("lesser whitethroats") OR TITLE-ABS(myoxidae) OR TITLE-ABS(seabasses) OR TITLE-ABS(spariformes) OR TITLE-ABS(umbridae) OR TITLE-ABS("yellow boxfish") OR TITLE-ABS(anabantiformes) OR TITLE-ABS(aotidae) OR TITLE-ABS("common bleak") OR TITLE-ABS("common bleaks") OR TITLE-ABS("common rudd") OR TITLE-ABS("common rudds") OR TITLE-ABS("greater pipefish") OR TITLE-ABS(hapale) OR TITLE-ABS(nandiniidae) OR TITLE-ABS("stone loaches") OR TITLE-ABS(whinchat) OR TITLE-ABS(whinchats) OR

		TITLE-ABS(acanthuriformes) OR TITLE-ABS("brotula barbata") OR TITLE-ABS("common ling") OR TITLE-ABS("common lings") OR TITLE-ABS("common roaches") OR TITLE-ABS(cottonrat) OR TITLE-ABS(cottonrats) OR TITLE-ABS(douroucoulis) OR TITLE-ABS(dromaiidae) OR TITLE-ABS(fitches) OR TITLE-ABS(fitchew) OR TITLE-ABS(galaxiiformes) OR TITLE-ABS(laprine) OR TITLE-ABS(saimiriinae) OR TITLE-ABS(solenette) OR TITLE-ABS(tarsii) OR TITLE-ABS("tompot blenny") OR TITLE-ABS("common dragonet") OR TITLE-ABS("common dragonets") OR TITLE-ABS("longspined bullhead") OR TITLE-ABS("longspined bullheads") OR TITLE-ABS(monotremate) OR TITLE-ABS(monotremates) OR TITLE-ABS(pempheriformes) OR TITLE-ABS(perdicinae) OR TITLE-ABS(presbytini) OR TITLE-ABS(smegmamorpha) OR TITLE-ABS("bighead gobies") OR TITLE-ABS("carangaria incertae sedis") OR TITLE-ABS(coiidae) OR TITLE-ABS("fivebeard rockling") OR TITLE-ABS(foulmart) OR TITLE-ABS(foumart) OR TITLE-ABS(grasskeet) OR TITLE-ABS("greater pipefishes") OR TITLE-ABS(ibices) OR TITLE-ABS(millionfish) OR TITLE-ABS(muguliformes) OR TITLE-ABS("norwegian topknot") OR TITLE-ABS(peewit) OR TITLE-ABS("red sea sailfin tang") OR TITLE-ABS(rupicapras) OR TITLE-ABS(sheatfishes) OR TITLE-ABS("tompot blennies") OR TITLE-ABS("twait shad") OR TITLE-ABS("yellow boxfishes")) AND NOT ALL(medline)))
#5	Cardiac AND repair AND ECM and animals 1,749 hits	#1 AND #2 AND #3 AND #4
#6	Remove reviews 1,528 hits	#5 AND (LIMIT-TO (DOCTYPE , "ar") OR LIMIT-TO (DOCTYPE , "cp") OR LIMIT-TO (DOCTYPE , "er"))
#7	*Retrieve reviews for separate screening* 215 hits	#5 AND (LIMIT-TO (DOCTYPE , "re") OR LIMIT-TO (DOCTYPE , "ch") OR LIMIT-TO (DOCTYPE , "bk") OR LIMIT-TO (DOCTYPE , "cr"))

Supplementary References

1. Abdalla, S., Makhoul, G., Duong, M., Chiu, R. C. J., & Cecere, R. (2013). Hyaluronic acid-based hydrogel induces neovascularization and improves cardiac function in a rat model of myocardial infarction. Interactive Cardiovascular and Thoracic Surgery, 17(5), 767–772. https://doi.org/10.1093/ICVTS/IVT277
2. Ahmadi, A., McNeill, B., Vulesevic, B., Kordos, M., Mesana, L., Thorn, S., Renaud, J. M., Manthorp, E., Kuraitis, D., Toeg, H., Mesana, T. G., Davis, D. R., Beanlands, R. S., DaSilva, J. N., deKemp, R. A., Ruel, M., & Suuronen, E. J. (2014). The role of integrin α2 in cell and matrix therapy that improves perfusion, viability and function of infarcted myocardium. Biomaterials, 35(17), 4749–4758. https://doi.org/10.1016/J.BIOMATERIALS.2014.02.028
3. Araña, M., Gavira, J. J., Peña, E., González, A., Abizanda, G., Cilla, M., Pérez, M. M., Albiasu, E., Aguado, N., Casado, M., López, B., González, S., Soriano, M., Moreno, C., Merino, J., García-Verdugo, J., Díez, J., Doblaré, M., Pelacho, B., & Prosper, F. (2014). Epicardial delivery of collagen patches with adipose-derived stem cells in rat and minipig models of chronic myocardial infarction. Biomaterials, 35(1), 143–151. https://doi.org/10.1016/J.BIOMATERIALS.2013.09.083
4. Aubin, H., Rath, L., Vey, A., Schmidt, V., Barth, M., Weber, E., Lichtenberg, A., & Akhyari, P. (2022). Ventricular stabilization with a customized decellularized cardiac ECM-based scaffold after myocardial infarction alters gene expression in a rodent LAD-ligation model. Frontiers in Bioengineering and Biotechnology, 10. https://doi.org/10.3389/fbioe.2022.896269
5. Baehr, A., Umansky, K. B., Bassat, E., Jurisch, V., Klett, K., Bozoglu, T., Hornaschewitz, N., Solyanik, O., Kain, D., Ferraro, B., Cohen-Rabi, R., Krane, M., Cyran, C., Soehnlein, O., Laugwitz, K. L., Hinkel, R., Kupatt, C., & Tzahor, E. (2020). Agrin Promotes Coordinated Therapeutic Processes Leading to Improved Cardiac Repair in Pigs. Circulation, 142(9), 868–881. https://doi.org/10.1161/CIRCULATIONAHA.119.045116
6. Bai, R., Tian, L., Li, Y., Zhang, J., Wei, Y., Jin, Z., Liu, Z., & Liu, H. (2019). Combining ECM Hydrogels of Cardiac Bioactivity with Stem Cells of High Cardiomyogenic Potential for Myocardial Repair. Stem Cells International, 2019. https://doi.org/10.1155/2019/6708435
7. Bassat, E., Mutlak, Y. E., Genzelinakh, A., Shadrin, I. Y., Baruch Umansky, K., Yifa, O., Kain, D., Rajchman, D., Leach, J., Riabov Bassat, D., Udi, Y., Sarig, R., Sagi, I., Martin, J. F., Bursac, N., Cohen, S., & Tzahor, E. (2017). The extracellular matrix protein agrin promotes heart regeneration in mice. Nature, 547(7662), 179–184. https://doi.org/10.1038/NATURE22978
8. Blackburn, N. J. R., Sofrenovic, T., Kuraitis, D., Ahmadi, A., McNeill, B., Deng, C., Rayner, K. J., Zhong, Z., Ruel, M., & Suuronen, E. J. (2015). Timing underpins the benefits associated with injectable collagen biomaterial therapy for the treatment of myocardial infarction. Biomaterials, 39, 182–192. https://doi.org/10.1016/J.BIOMATERIALS.2014.11.004
9. Chang, M.-Y., Huang, T.-T., Chen, C.-H., Cheng, B., Hwang, S.-M., & Hsieh, P. C. H. (2016). Injection of Human Cord Blood Cells With Hyaluronan Improves Postinfarction Cardiac Repair in Pigs. Stem Cells Translational Medicine, 5(1), 56–66. https://doi.org/10.5966/SCTM.2015-0092
10. Chen, C. H., Chang, M. Y., Wang, S. S., & Hsieh, P. C. H. (2014). Injection of autologous bone marrow cells in hyaluronan hydrogel improves cardiac performance after infarction in pigs. American Journal of Physiology - Heart and Circulatory Physiology, 306(7). https://doi.org/10.1152/AJPHEART.00801.2013

11. Chen, C. H., Wang, S. S., Wei, E. I. H., Chu, T. Y., & Hsieh, P. C. H. (2013). Hyaluronan enhances bone marrow cell therapy for myocardial repair after infarction. Molecular Therapy, 21(3), 670–679. https://doi.org/10.1038/MT.2012.268
12. Chen, C. H., Wei, H. J., Lin, W. W., Chiu, I., Hwang, S. M., Wang, C. C., Lee, W. Y., Chang, Y., & Sung, H. W. (2008). Porous tissue grafts sandwiched with multilayered mesenchymal stromal cell sheets induce tissue regeneration for cardiac repair. Cardiovascular Research, 80(1), 88–95. https://doi.org/10.1093/CVR/CVN149
13. Chen, W. C. W., Wang, Z., Missinato, M. A., Park, D. W., Long, D. W., Liu, H. J., Zeng, X., Yates, N. A., Kim, K., & Wang, Y. (2016). Decellularized zebrafish cardiac extracellular matrix induces mammalian heart regeneration. Science Advances, 2(11). https://doi.org/10.1126/SCIADV.1600844
14. Cortes-Morichetti, M., Frati, G., Schussler, O., Van Huyen, J. P. D., Lauret, E., Genovese, J. A., Carpentier, A. F., & Chachques, J. C. (2007). Association between a cell-seeded collagen matrix and cellular cardiomyoplasty for myocardial support and regeneration. Tissue Engineering, 13(11), 2681–2687. https://doi.org/10.1089/TEN.2006.0447
15. D'Amore, A., Yoshizumi, T., Luketich, S. K., Wolf, M. T., Gu, X., Cammarata, M., Hoff, R., Badylak, S. F., & Wagner, W. R. (2016). Bi-layered polyurethane - Extracellular matrix cardiac patch improves ischemic ventricular wall remodeling in a rat model. Biomaterials, 107, 1–14. https://doi.org/10.1016/J.BIOMATERIALS.2016.07.039
16. Feng, J., Li, Y., Li, Y., Yin, Q., Li, H., Li, J., Zhou, B., Meng, J., Lian, H., Wu, M., Li, Y., Dou, K., Song, W., Lu, B., Liu, L., Hu, S., & Nie, Y. (2024). Versican Promotes Cardiomyocyte Proliferation and Cardiac Repair. Circulation, 149(13), 1004–1015. https://doi.org/10.1161/CIRCULATIONAHA.123.066298
17. Feng, L., Ling, C., Yong, G., Jianye, Z., & Qingqing, S. (2024). Hyaluronic acid hydrogel-encapsulated bone marrow mesenchymal stem cells promote cardiac function in myocardial infarction rats (III). Chinese Journal of Tissue Engineering Research, 28(3), 355–359.
18. Francis, M. P., Breathwaite, E., Bulysheva, A. A., Varghese, F., Rodriguez, R. U., Dutta, S., Semenov, I., Ogle, R., Huber, A., Tichy, A. M., Chen, S., & Zemlin, C. (2017). Human placenta hydrogel reduces scarring in a rat model of cardiac ischemia and enhances cardiomyocyte and stem cell cultures. Acta Biomaterialia, 52, 92–104. https://doi.org/10.1016/J.ACTBIO.2016.12.027
19. Francisco, J. C., Uemura, L., Simeoni, R. B., da Cunha, R. C., Mogharbel, B. F., Simeoni, P. R. B., Naves, G., Napimoga, M. H., Noronha, L., Carvalho, K. A. T., Moreira, L. F. P., & Guarita-Souza, L. C. (2020). Acellular human amniotic membrane scaffold with 15d-PGJ2 nanoparticles in postinfarct rat model. Tissue Engineering - Part A, 26(21–22), 1128–1137. https://doi.org/10.1089/TEN.TEA.2019.0340
20. Frederick, J. R., Fitzpatrick, J. R., McCormick, R. C., Harris, D. A., Kim, A. Y., Muenzer, J. R., Marotta, N., Smith, M. J., Cohen, J. E., Hiesinger, W., Atluri, P., & Joseph Woo, Y. (2010). Stromal cell-derived factor-1α activation of tissue-engineered endothelial progenitor cell matrix enhances ventricular function after myocardial infarction by inducing neovasculogenesis. Circulation, 122(11 SUPPL. 1). https://doi.org/10.1161/CIRCULATIONAHA.109.930404
21. Gaetani, R., Feyen, D. A. M., Verhage, V., Slaats, R., Messina, E., Christman, K. L., Giacomello, A., Doevendans, P. A. F. M., & Sluijter, J. P. G. (2015). Epicardial application of cardiac progenitor cells in a 3D-printed gelatin/hyaluronic acid patch

preserves cardiac function after myocardial infarction. Biomaterials, 61, 339–348. https://doi.org/10.1016/J.BIOMATERIALS.2015.05.005
22. Gaffey, A. C., Chen, M. H., Venkataraman, C. M., Trubelja, A., Rodell, C. B., Dinh, P. V., Hung, G., Macarthur, J. W., Soopan, R. V., Burdick, J. A., & Atluri, P. (2015). Injectable shear-thinning hydrogels used to deliver endothelial progenitor cells, enhance cell engraftment, and improve ischemic myocardium. Journal of Thoracic and Cardiovascular Surgery, 150(5), 1268–1277. https://doi.org/10.1016/J.JTCVS.2015.07.035
23. Gálvez-Montón, C., Fernandez-Figueras, M. T., Martí, M., Soler-Botija, C., Roura, S., Perea-Gil, I., Prat-Vidal, C., Llucià-Valldeperas, A., Raya, Á., & Bayes-Genis, A. (2015). Neoinnervation and neovascularization of acellular pericardial-derived scaffolds in myocardial infarcts. Stem Cell Research and Therapy, 6(1). https://doi.org/10.1186/S13287-015-0101-6
24. Gao, L., Kupfer, M. E., Jung, J. P., Yang, L., Zhang, P., Da Sie, Y., Tran, Q., Ajeti, V., Freeman, B. T., Fast, V. G., Campagnola, P. J., Ogle, B. M., & Zhang, J. (2017). Myocardial Tissue Engineering with Cells Derived from Human-Induced Pluripotent Stem Cells and a Native-Like, High-Resolution, 3-Dimensionally Printed Scaffold. Circulation Research, 120(8), 1318–1325. https://doi.org/10.1161/CIRCRESAHA.116.310277
25. Guan, G., Huo, D., Li, Y., Zhao, X., Li, Y., Qin, Z., Sun, D., Yang, G., Yang, M., Tan, J., Zeng, W., & Zhu, C. (2021). Engineering hiPSC-CM and hiPSC-EC laden 3D nanofibrous splenic hydrogel for improving cardiac function through revascularization and remuscularization in infarcted heart. Bioactive Materials, 6(12), 4415–4429. https://doi.org/10.1016/J.BIOACTMAT.2021.04.010
26. Hao, D.-J., Qin, Y., Zhou, S.-J., Dong, B.-H., Yang, J.-S., Zou, P., Wang, L.-P., & Zhao, Y.-T. (2024). Hapln1 promotes dedifferentiation and proliferation of iPSC-derived cardiomyocytes by promoting versican-based GDF11 trapping. Journal of Pharmaceutical Analysis, 14(3), 335–347. https://doi.org/10.1016/j.jpha.2023.09.013
27. Hao, Y., Zhang, W., Qin, J., Tan, L., Luo, Y., & Chen, H. (2022). Biological Cardiac Patch Based on Extracellular Vesicles and Extracellular Matrix for Regulating Injury-Related Microenvironment and Promoting Cardiac Tissue Recovery. ACS Applied Bio Materials, 5(11), 5218–5230. https://doi.org/10.1021/acsabm.2c00659
28. Hayam, R., Ertracht, O., Zahran, S., Baruch, L., Atar, S., & Machluf, M. (2022). Electrospun extracellular matrix scaffold improves cardiac structure and function post-myocardial infarction. Polymers for Advanced Technologies, 33(11), 3822–3831. https://doi.org/10.1002/pat.5777
29. Henry, J. J. D., Delrosario, L., Fang, J., Wong, S. Y., Fang, Q., Sievers, R., Kotha, S., Wang, A., Farmer, D., Janaswamy, P., Lee, R. J., & Li, S. (2020). Development of Injectable Amniotic Membrane Matrix for Postmyocardial Infarction Tissue Repair. Advanced Healthcare Materials, 9(2). https://doi.org/10.1002/ADHM.201900544
30. Hosoyama, K., Ahumada, M., McTiernan, C. D., Davis, D. R., Variola, F., Ruel, M., Liang, W., Suuronen, E. J., & Alarcon, E. I. (2018). Nanoengineered Electroconductive Collagen-Based Cardiac Patch for Infarcted Myocardium Repair. ACS Applied Materials and Interfaces, 10(51), 44668–44677. https://doi.org/10.1021/ACSAMI.8B18844/SUPPL_FILE/AM8B18844_SI_002.PDF
31. Huang, K., Ozpinar, E. W., Su, T., Tang, J., Shen, D., Qiao, L., Hu, S., Li, Z., Liang, H., Mathews, K., Scharf, V., Freytes, D. O., & Cheng, K. (2020). An off-the-shelf artificial cardiac patch improves cardiac repair after myocardial infarction in rats and

pigs. Science Translational Medicine, 12(538). https://doi.org/10.1126/SCITRANSLMED.AAT9683
32. Huang, N. F., Yu, J., Sievers, R., Li, S., & Lee, R. J. (2005). Injectable biopolymers enhance angiogenesis after myocardial infarction. Tissue Engineering, 11(11–12), 1860–1866. https://doi.org/10.1089/TEN.2005.11.1860
33. Hume, R. D., Kanagalingam, S., Deshmukh, T., Chen, S., Mithieux, S. M., Rashid, F. N., Roohani, I., Lu, J., Doan, T., Graham, D., Clayton, Z. E., Slaughter, E., Kizana, E., Stempien-Otero, A. S., Brown, P., Thomas, L., Weiss, A. S., & Chong, J. J. H. (2023). Tropoelastin Improves Post-Infarct Cardiac Function. Circulation Research, 132(1), 72–86. https://doi.org/10.1161/CIRCRESAHA.122.321123
34. Jiang, Y., Zhang, L.-L., Zhang, F., Bi, W., Zhang, P., Yu, X.-J., Rao, S.-L., Wang, S.-H., Li, Q., Ding, C., Jin, Y., Liu, Z.-M., & Yang, H.-T. (2023). Dual human iPSC-derived cardiac lineage cell-seeding extracellular matrix patches promote regeneration and long-term repair of infarcted hearts. Bioactive Materials, 28, 206–226. https://doi.org/10.1016/j.bioactmat.2023.05.015
35. Kashiyama, N., Kormos, R. L., Matsumura, Y., D'Amore, A., Miyagawa, S., Sawa, Y., & Wagner, W. R. (2022). Adipose-derived stem cell sheet under an elastic patch improves cardiac function in rats after myocardial infarction. Journal of Thoracic and Cardiovascular Surgery, 163(4), e261–e272. https://doi.org/10.1016/J.JTCVS.2020.04.150
36. Kofidis, T., De Bruin, J. L., Hoyt, G., Ho, Y., Tanaka, M., Yamane, T., Lebl, D. R., Swijnenburg, R. J., Chang, C. P., Quertermous, T., & Robbins, R. C. (2005). Myocardial restoration with embryonic stem cell bioartificial tissue transplantation. Journal of Heart and Lung Transplantation, 24(6), 737–744. https://doi.org/10.1016/J.HEALUN.2004.03.023
37. Kofidis, T., Lebl, D. R., Martinez, E. C., Hoyt, G., Tanaka, M., & Robbins, R. C. (2005). Novel injectable bioartificial tissue facilitates targeted, less invasive, large-scale tissue restoration on the beating heart after myocardial injury. Circulation, 112(9 Suppl). https://doi.org/10.1161/CIRCULATIONAHA.104.526178
38. Le, L. V., Mohindra, P., Fang, Q., Sievers, R. E., Mkrtschjan, M. A., Solis, C., Safranek, C. W., Russell, B., Lee, R. J., & Desai, T. A. (2018). Injectable hyaluronic acid based microrods provide local micromechanical and biochemical cues to attenuate cardiac fibrosis after myocardial infarction. Biomaterials, 169, 11–21. https://doi.org/10.1016/J.BIOMATERIALS.2018.03.042
39. Lebedeva, A. I., Muslimov, S. A., Gareev, E. M., Popov, S. V., Afanas'ev, S. A., & Kondrat'eva, D. S. (2018). Experimental Cardiomyogenesis Under Conditions of Administration of Different Doses of the Allogeneic Biomaterial. Bulletin of Experimental Biology and Medicine, 165(6), 790–792. https://doi.org/10.1007/S10517-018-4266-8
40. Lebedeva, A. I., Muslimov, S. A., Musina, L. A., Gareev, E. M., Kadyrov, R. Z., Condratyeva, D. S., Afanasiev, S. A., & Popov, S. V. (2020). Effect of intramyocardial allogenic biomaterial injection on angiogenesis and postischemic scar remodeling in rats. Vestnik Transplantologii i Iskusstvennykh Organov, 22(3), 156–166. https://doi.org/10.15825/1995-1191-2020-3-156-166
41. Lee, J., Lee, S., Kim, B., Park, S., Sundaram, M. N., Kim, B., Kim, C., & Hwang, N. S. (2024). Paintable Decellularized-ECM Hydrogel for Preventing Cardiac Tissue Damage. Advanced Science, 11(21). https://doi.org/10.1002/advs.202307353
42. Lee, J., Song, M., Kim, J., & Park, Y. (2018). Comparison of Angiogenic Activities of Three Neuropeptides, Substance P, Secretoneurin, and Neuropeptide Y Using Myocardial Infarction. Tissue Engineering and Regenerative Medicine, 15(4), 493–502. https://doi.org/10.1007/S13770-018-0134-X

43. Lindsey, M. L., Iyer, R. P., Zamilpa, R., Yabluchanskiy, A., DeLeon-Pennell, K. Y., Hall, M. E., Kaplan, A., Zouein, F. A., Bratton, D., Flynn, E. R., Cannon, P. L., Tian, Y., Jin, Y. F., Lange, R. A., Tokmina-Roszyk, D., Fields, G. B., & De Castro Brás, L. E. (2015). A Novel Collagen Matricryptin Reduces Left Ventricular Dilation Post-Myocardial Infarction by Promoting Scar Formation and Angiogenesis. Journal of the American College of Cardiology, 66(12), 1364–1374. https://doi.org/10.1016/J.JACC.2015.07.035
44. Macarthur, J. W., Purcell, B. P., Shudo, Y., Cohen, J. E., Fairman, A., Trubelja, A., Patel, J., Hsiao, P., Yang, E., Lloyd, K., Hiesinger, W., Atluri, P., Burdick, J. A., & Woo, Y. J. (2013). Sustained release of engineered stromal cell-derived factor 1-α from injectable hydrogels effectively recruits endothelial progenitor cells and preserves ventricular function after myocardial infarction. Circulation, 128(11 Suppl 1). https://doi.org/10.1161/CIRCULATIONAHA.112.000343
45. McLaughlin, S., McNeill, B., Podrebarac, J., Hosoyama, K., Sedlakova, V., Cron, G., Smyth, D., Seymour, R., Goel, K., Liang, W., Rayner, K. J., Ruel, M., Suuronen, E. J., & Alarcon, E. I. (2019). Injectable human recombinant collagen matrices limit adverse remodeling and improve cardiac function after myocardial infarction. Nature Communications, 10(1). https://doi.org/10.1038/S41467-019-12748-8
46. Mewhort, H. E. M., Turnbull, J. D., Meijndert, H. C., Ngu, J. M. C., & Fedak, P. W. M. (2014). Epicardial infarct repair with basic fibroblast growth factor-enhanced CorMatrix-ECM biomaterial attenuates postischemic cardiac remodeling. Journal of Thoracic and Cardiovascular Surgery, 147(5), 1650–1659. https://doi.org/10.1016/J.JTCVS.2013.08.005
47. Mewhort, H. E. M., Turnbull, J. D., Satriano, A., Chow, K., Flewitt, J. A., Andrei, A. C., Guzzardi, D. G., Svystonyuk, D. A., White, J. A., & Fedak, P. W. M. (2016). Epicardial infarct repair with bioinductive extracellular matrix promotes vasculogenesis and myocardial recovery. Journal of Heart and Lung Transplantation, 35(5), 661–670. https://doi.org/10.1016/J.HEALUN.2016.01.012
48. Mohindra, P., Zhong, J. X., Fang, Q., Cuylear, D. L., Huynh, C., Qiu, H., Gao, D., Kharbikar, B. N., Huang, X., Springer, M. L., Lee, R. J., & Desai, T. A. (2023). Local decorin delivery via hyaluronic acid microrods improves cardiac performance, ventricular remodeling after myocardial infarction. Npj Regenerative Medicine, 8(1), 60. https://doi.org/10.1038/s41536-023-00336-w
49. Muscari, C., Bonafè, F., Martin-Suarez, S., Valgimigli, S., Valente, S., Fiumana, E., Fiorelli, F., Rubini, G., Guarnieri, C., Caldarera, C. M., Capitani, O., Arpesella, G., & Pasquinelli, G. (2013). Restored perfusion and reduced inflammation in the infarcted heart after grafting stem cells with a hyaluronan-based scaffold. Journal of Cellular and Molecular Medicine, 17(4), 518–530. https://doi.org/10.1111/JCMM.12039
50. Nair, R. S., Sobhan, P. K., Shenoy, S. J., Prabhu, M. A., Kumar, V., Ramachandran, S., & Anilkumar, T. V. (2023). Mitigation of Fibrosis after Myocardial Infarction in Rats by Using a Porcine Cholecyst Extracellular Matrix. Comparative Medicine, 73(4), 311–322. https://doi.org/10.30802/AALAS-CM-22-000097
51. Neuta, P. A., Rojas, D. M., Agredo, W., & Gutierrez, J. O. (2015). Evaluation of the repairing effect of collagen type I and MaxGel on the infarcted myocardium in an animal model. Annual International Conference of the IEEE Engineering in Medicine and Biology Society. IEEE Engineering in Medicine and Biology Society. Annual International Conference, 2015, 3529–3532. https://doi.org/10.1109/EMBC.2015.7319154

52. Noshadi, I., Hong, S., Sullivan, K. E., Shirzaei Sani, E., Portillo-Lara, R., Tamayol, A., Shin, S. R., Gao, A. E., Stoppel, W. L., Black, L. D., Khademhosseini, A., & Annabi, N. (2017). In vitro and in vivo analysis of visible light crosslinkable gelatin methacryloyl (GelMA) hydrogels. Biomaterials Science, 5(10), 2093–2105. https://doi.org/10.1039/C7BM00110J
53. Okada, M., Payne, T. R., Oshima, H., Momoi, N., Tobita, K., & Huard, J. (2010). Differential efficacy of gels derived from small intestinal submucosa as an injectable biomaterial for myocardial infarct repair. Biomaterials, 31(30), 7678–7683. https://doi.org/10.1016/J.BIOMATERIALS.2010.06.056
54. Perea-Gil, I., Gálvez-Montón, C., Prat-Vidal, C., Jorba, I., Segú-Vergés, C., Roura, S., Soler-Botija, C., Iborra-Egea, O., Revuelta-López, E., Fernández, M. A., Farré, R., Navajas, D., & Bayes-Genis, A. (2018). Head-to-head comparison of two engineered cardiac grafts for myocardial repair: From scaffold characterization to pre-clinical testing. Scientific Reports, 8(1). https://doi.org/10.1038/S41598-018-25115-2
55. Pupkaite, J., Sedlakova, V., Eren Cimenci, C., Bak, M., McLaughlin, S., Ruel, M., Alarcon, E. I., & Suuronen, E. J. (2020). Delivering More of an Injectable Human Recombinant Collagen III Hydrogel Does Not Improve Its Therapeutic Efficacy for Treating Myocardial Infarction. ACS Biomaterials Science and Engineering, 6(7), 4256–4265. https://doi.org/10.1021/ACSBIOMATERIALS.0C00418
56. Qiao, L., Kong, Y., Shi, Y., Sun, A., Ji, R., Huang, C., Li, Y., & Yang, X. (2019). Synergistic effects of adipose-derived stem cells combined with decellularized myocardial matrix on the treatment of myocardial infarction in rats. Life Sciences, 239. https://doi.org/10.1016/J.LFS.2019.116891
57. Ravi, S., Caves, J. M., Martinez, A. W., Xiao, J., Wen, J., Haller, C. A., Davis, M. E., & Chaikof, E. L. (2012). Effect of bone marrow-derived extracellular matrix on cardiac function after ischemic injury. Biomaterials, 33(31), 7736–7745. https://doi.org/10.1016/J.BIOMATERIALS.2012.07.010
58. Rotem, I., Konfino, T., Caller, T., Schary, Y., Shaihov-Teper, O., Palevski, D., Lewis, N., Lendengolts, D., Naftali-Shani, N., & Leor, J. (2022). Osteopontin promotes infarct repair. Basic Research in Cardiology, 117(1), 51. https://doi.org/10.1007/s00395-022-00957-0
59. Sarig, U., Sarig, H., de-Berardinis, E., Chaw, S. Y., Nguyen, E. B. V., Ramanujam, V. S., Thang, V. D., Al-Haddawi, M., Liao, S., Seliktar, D., Kofidis, T., Boey, F. Y. C., Venkatraman, S. S., & Machluf, M. (2016). Natural myocardial ECM patch drives cardiac progenitor based restoration even after scarring. Acta Biomaterialia, 44, 209–220. https://doi.org/10.1016/J.ACTBIO.2016.08.031
60. Seif-Naraghi, S. B., Horn, D., Schup-Magoffin, P. J., & Christman, K. L. (2012). Injectable extracellular matrix derived hydrogel provides a platform for enhanced retention and delivery of a heparin-binding growth factor. Acta Biomaterialia, 8(10), 3695–3703. https://doi.org/10.1016/J.ACTBIO.2012.06.030
61. Seif-Naraghi, S. B., Singelyn, J. M., Salvatore, M. A., Osborn, K. G., Wang, J. J., Sampat, U., Kwan, O. L., Strachan, G. M., Wong, J., Schup-Magoffin, P. J., Braden, R. L., Bartels, K., DeQuach, J. A., Preul, M., Kinsey, A. M., DeMaria, A. N., Dib, N., & Christman, K. L. (2013). Safety and efficacy of an injectable extracellular matrix hydrogel for treating myocardial infarction. Science Translational Medicine, 5(173). https://doi.org/10.1126/SCITRANSLMED.3005503
62. Serpooshan, V., Zhao, M., Metzler, S. A., Wei, K., Shah, P. B., Wang, A., Mahmoudi, M., Malkovskiy, A. V., Rajadas, J., Butte, M. J., Bernstein, D., & Ruiz-Lozano, P. (2013). The effect of bioengineered acellular collagen patch on cardiac

remodeling and ventricular function post myocardial infarction. Biomaterials, 34(36), 9048–9055. https://doi.org/10.1016/J.BIOMATERIALS.2013.08.017

63. Shen, D., Wang, X., Zhang, L., Zhao, X., Li, J., Cheng, K., & Zhang, J. (2011). The amelioration of cardiac dysfunction after myocardial infarction by the injection of keratin biomaterials derived from human hair. Biomaterials, 32(35), 9290–9299. https://doi.org/10.1016/J.BIOMATERIALS.2011.08.057
64. Shepherd, B. R., Hoying, J. B., & Williams, S. K. (2007). Microvascular transplantation after acute myocardial infarction. Tissue Engineering, 13(12), 2871–2879. https://doi.org/10.1089/TEN.2007.0025
65. Shi, J., Fan, C., Zhuang, Y., Sun, J., Hou, X., Chen, B., Xiao, Z., Chen, Y., Zhan, Z., Zhao, Y., & Dai, J. (2019). Heparan sulfate proteoglycan promotes fibroblast growth factor-2 function for ischemic heart repair. Biomaterials Science, 7(12), 5438–5450. https://doi.org/10.1039/C9BM01336A
66. Simpson, D., Liu, H., Fan, T.-H. M., Nerem, R., & Dudley, S. C. (2007). A Tissue Engineering Approach to Progenitor Cell Delivery Results in Significant Cell Engraftment and Improved Myocardial Remodeling. Stem Cells, 25(9), 2350–2357. https://doi.org/10.1634/STEMCELLS.2007-0132
67. So, J. Y., Yong, H. F., Choon, H. L., Bum, S. K., Ho, S. S., Park, Y., & Sun, K. (2009). Regeneration of ischemic heart using hyaluronic acid-based injectable hydrogel. Journal of Biomedical Materials Research - Part B Applied Biomaterials, 91(1), 163–171. https://doi.org/10.1002/JBM.B.31386
68. Spang, M. T., Middleton, R., Diaz, M., Hunter, J., Mesfin, J., Banka, A., Sullivan, H., Wang, R., Lazerson, T. S., Bhatia, S., Corbitt, J., D'Elia, G., Sandoval-Gomez, G., Kandell, R., Vratsanos, M. A., Gnanasekaran, K., Kato, T., Igata, S., Luo, C., … Christman, K. L. (2022). Intravascularly infused extracellular matrix as a biomaterial for targeting and treating inflamed tissues. Nature Biomedical Engineering, 7(2), 94–109. https://doi.org/10.1038/s41551-022-00964-5
69. Svystonyuk, D. A., Mewhort, H. E. M., Hassanabad, A. F., Heydari, B., Mikami, Y., Turnbull, J. D., Teng, G., Belke, D. D., Wagner, K. T., Tarraf, S. A., DiMartino, E. S., White, J. A., Flewitt, J. A., Cheung, M., Guzzardi, D. G., Kang, S., & Fedak, P. W. M. (2020). Acellular bioscaffolds redirect cardiac fibroblasts and promote functional tissue repair in rodents and humans with myocardial injury. Scientific Reports, 10(1). https://doi.org/10.1038/S41598-020-66327-9
70. Tang, J., Vandergriff, A., Wang, Z., Hensley, M. T., Cores, J., Allen, T. A., Dinh, P. U., Zhang, J., Caranasos, T. G., & Cheng, K. (2017). A Regenerative Cardiac Patch Formed by Spray Painting of Biomaterials onto the Heart. Tissue Engineering - Part C: Methods, 23(3), 146–155. https://doi.org/10.1089/TEN.TEC.2016.0492
71. Tashakori-Miyanroudi, M., Rakhshan, K., Ramez, M., Asgarian, S., Janzadeh, A., Azizi, Y., Seifalian, A., & Ramezani, F. (2020). Conductive carbon nanofibers incorporated into collagen bio-scaffold assists myocardial injury repair. International Journal of Biological Macromolecules, 163, 1136–1146. https://doi.org/10.1016/J.IJBIOMAC.2020.06.259
72. Toeg, H. D., Tiwari-Pandey, R., Seymour, R., Ahmadi, A., Crowe, S., Vulesevic, B., Suuronen, E. J., & Ruel, M. (2013). Injectable small intestine submucosal extracellular matrix in an acute myocardial infarction model. Annals of Thoracic Surgery, 96(5), 1686–1694. https://doi.org/10.1016/J.ATHORACSUR.2013.06.063
73. Vasanthan, V., Hassanabad, A. F., Belke, D., Teng, G., Isidoro, C. A., Dutta, D., Turnbull, J., Deniset, J. F., & Fedak, P. W. M. (2023). Micronized Acellular Matrix Biomaterial Leverages Eosinophils for Postinfarct Cardiac Repair. JACC: Basic to Translational Science, 8(8), 939–954. https://doi.org/10.1016/j.jacbts.2023.01.012

74. Vasanthan, V., Shim, H. B., Teng, G., Belke, D., Svystonyuk, D., Deniset, J. F., & Fedak, P. W. M. (2023). Acellular biomaterial modulates myocardial inflammation and promotes endogenous mechanisms of postinfarct cardiac repair. Journal of Thoracic and Cardiovascular Surgery, 165(3), e122–e140. https://doi.org/10.1016/J.JTCVS.2021.12.036
75. Vu, T. D., Pal, S. N., Ti, L. K., Martinez, E. C., Rufaihah, A. J., Ling, L. H., Lee, C. N., Richards, A. M., & Kofidis, T. (2015). An autologous platelet-rich plasma hydrogel compound restores left ventricular structure, function and ameliorates adverse remodeling in a minimally invasive large animal myocardial restoration model: A translational approach. Vu and Pal "Myocardial Repair: PRP, Hydrogel and Supplements." Biomaterials, 45, 27–35. https://doi.org/10.1016/J.BIOMATERIALS.2014.12.013
76. Wang, N., Liu, C., Wang, X., He, T., Li, L., Liang, X., Wang, L., Song, L., Wei, Y., Wu, Q., & Gong, C. (2019). Hyaluronic Acid Oligosaccharides Improve Myocardial Function Reconstruction and Angiogenesis against Myocardial Infarction by Regulation of Macrophages. Theranostics, 9(7), 1980–1992. https://doi.org/10.7150/THNO.31073
77. Wang, Q., Yang, H., Bai, A., Jiang, W., Li, X., Wang, X., Mao, Y., Lu, C., Qian, R., Guo, F., Ding, T., Chen, H., Chen, S., Zhang, J., Liu, C., & Sun, N. (2016). Functional engineered human cardiac patches prepared from nature's platform improve heart function after acute myocardial infarction. Biomaterials, 105, 52–65. https://doi.org/10.1016/J.BIOMATERIALS.2016.07.035
78. Wang, R. M., Mesfin, J. M., Hunter, J., Cattaneo, P., Guimarães-Camboa, N., Braden, R. L., Luo, C., Hill, R. C., Dzieciatkowska, M., Hansen, K. C., Evans, S., & Christman, K. L. (2022). Myocardial matrix hydrogel acts as a reactive oxygen species scavenger and supports a proliferative microenvironment for cardiomyocytes. Acta Biomaterialia, 152, 47–59. https://doi.org/10.1016/j.actbio.2022.08.050
79. Wang, Y., Wang, J., Liu, C., Li, J., Lu, K., Yu, Q., Zhang, Y., & Shen, Z. (2023). Injectable decellularized extracellular matrix hydrogel loaded with exosomes encapsulating curcumin for prevention of cardiac fibrosis after myocardial infarction. Journal of Materials Science & Technology, 167, 50–58. https://doi.org/10.1016/j.jmst.2023.06.005
80. Wang, Z., Long, D. W., Huang, Y., Chen, W. C. W., Kim, K., & Wang, Y. (2019). Decellularized neonatal cardiac extracellular matrix prevents widespread ventricular remodeling in adult mammals after myocardial infarction. Acta Biomaterialia, 87, 140–151. https://doi.org/10.1016/J.ACTBIO.2019.01.062
81. Wassenaar, J. W., Gaetani, R., Garcia, J. J., Braden, R. L., Luo, C. G., Huang, D., Demaria, A. N., Omens, J. H., & Christman, K. L. (2016). Evidence for mechanisms underlying the functional benefits of a myocardial matrix hydrogel for post-MI treatment. Journal of the American College of Cardiology, 67(9), 1074–1086. https://doi.org/10.1016/J.JACC.2015.12.035
82. Xiang, Z., Liao, R., Kelly, M. S., & Spector, M. (2006). Collagen-GAG scaffolds grafted onto myocardial infarcts in a rat model: a delivery vehicle for mesenchymal stem cells. Tissue Engineering, 12(9), 2467–2478. https://doi.org/10.1089/TEN.2006.12.2467
83. Xie, Y., Lampinen, M., Takala, J., Sikorski, V., Soliymani, R., Tarkia, M., Lalowski, M., Mervaala, E., Kupari, M., Zheng, Z., Hu, S., Harjula, A., & Kankuri, E. (2020). Epicardial transplantation of atrial appendage micrograft patch salvages myocardium after infarction. Journal of Heart and Lung Transplantation, 39(7), 707–718. https://doi.org/10.1016/J.HEALUN.2020.03.023

84. Yang, X., Chen, S., Chen, J., Liu, Y., Bai, Y., Yin, S., & Quan, D. (2021). The different effect of decellularized myocardial matrix hydrogel and decellularized small intestinal submucosa matrix hydrogel on cardiomyocytes and ischemic heart. Applied Sciences (Switzerland), 11(17). https://doi.org/10.3390/APP11177768
85. Yoon, S. J., Hong, S., Fang, Y. H., Song, M., Son, K. H., Son, H. S., Kim, S. K., Sun, K., & Park, Y. (2014). Differential regeneration of myocardial infarction depending on the progression of disease and the composition of biomimetic hydrogel. Journal of Bioscience and Bioengineering, 118(4), 461–468. https://doi.org/10.1016/J.JBIOSC.2014.04.001
86. Yu, J., Christman, K. L., Chin, E., Sievers, R. E., Saeed, M., & Lee, R. J. (2009). Restoration of left ventricular geometry and improvement of left ventricular function in a rodent model of chronic ischemic cardiomyopathy. Journal of Thoracic and Cardiovascular Surgery, 137(1), 180–187. https://doi.org/10.1016/J.JTCVS.2008.08.036
87. Zhang, L., Elkahal, J., Wang, T., Rimmer, R., Genzelinakh, A., Bassat, E., Wang, J., Perez, D., Kain, D., Lendengolts, D., Winkler, R., Bueno-levy, H., Umansky, K. B., Mishaly, D., Shakked, A., Miyara, S., Sarusi-Portuguez, A., Goldfinger, N., Prior, A., … Tzahor, E. (2024). Egr1 regulates regenerative senescence and cardiac repair. Nature Cardiovascular Research. https://doi.org/10.1038/s44161-024-00493-1
88. Zhang, Y., Zhu, D., Wei, Y., Wu, Y., Cui, W., Liuqin, L., Fan, G., Yang, Q., Wang, Z., Xu, Z., Kong, D., Zeng, L., & Zhao, Q. (2019). A collagen hydrogel loaded with HDAC7-derived peptide promotes the regeneration of infarcted myocardium with functional improvement in a rodent model. Acta Biomaterialia, 86, 223–234. https://doi.org/10.1016/J.ACTBIO.2019.01.022
