# Supplementary material for: Preclinical extracellular matrix-based treatment strategies for myocardial infarction: a systematic review and meta-analysis
Source: Commun Med (Lond). 2025 Mar 30;5:95. doi: 10.1038/s43856-025-00812-y (PMC11955565; doi:10.1038/s43856-025-00812-y)
Supplement: Supplementary file 2 — Description of Additional Supplementary Files [file 43856_2025_812_MOESM2_ESM.pdf]

Description of Additional Supplementary Files

File name: Supplementary Data 1

Description: Table of study characteristics of all 88 included studies in this systematic review.

File name: Supplementary Data 2:

Description: Table of risk of bias assessment of all 88 included studies in this systematic review.

File name: Supplementary Data 3

Description: Table of study characteristics all 60 studies which mentioned LVEF

File name: Supplementary Data 4

Description: Table of study characteristics of all 16 studies which mentioned animal survival

File name: Supplementary Data 5

Description: Table of study characteristics of all 39 studies which mentioned fractional shortening

File name: Supplementary Data 6

Description: Table of study characteristics of all 12 studies which mentioned stroke volume

File name: Supplementary Data 7

Description: Table of study characteristics of all 51 studies which mentioned infarct size

File name: Supplementary Data 8

Description: Table of study characteristics of all 30 studies which mentioned left ventricular wall thickening

File name: Supplementary Figure 1

Description: Publication Bias of the included studies.

File name: Supplementary Figure 2

Description: Forest plot showing the effect of ECM treatment on Left Ventricular Ejection Fraction.

File name: Supplementary Figure 3

Description: Forest plot showing the effect of ECM treatment on Fractional Shortening.

File name: Supplementary Figure 4

Description: Forest plot showing the effect of ECM treatment on Stroke Volume.

File name: Supplementary Figure 5

Description: Forest plot showing the effect of ECM treatment on Infarct Size.

File name: Supplementary Figure 6

Description: Forest plot showing the effect of ECM treatment on Wall Thickening.

File name: Supplementary File 1

Description: Updated search 28-06-2024 for PUBMED

File name: Supplementary File 2

Description: Updated search 28-06-2024 for SCOPUS

File name: Supplementary File 3

Description: Original search 06-07-2022 for PUBMED

File name: Supplementary File 4

Description: Original search 06-07-2022 for SCOPUS

File name: Supplementary References

Description: References of included studies in this systematic review and meta-analysis
