# Supplementary material for: Preclinical extracellular matrix-based treatment strategies for myocardial infarction: a systematic review and meta-analysis
Source: Commun Med (Lond). 2025 Mar 30;5:95. doi: 10.1038/s43856-025-00812-y (PMC11955565; doi:10.1038/s43856-025-00812-y)
Supplement: Supplementary file 4 — reporting summary [file 43856_2025_812_MOESM4_ESM.pdf]

nature portfolio

Corresponding author(s): Atze van der Pol
Carlijn V.C. Bouten

Last updated by author(s): Mar 4, 2025

Reporting Summary

Nature Portfolio wishes to improve the reproducibility of the work that we publish. This form provides structure for consistency and transparency in reporting. For further information on Nature Portfolio policies, see our Editorial Policies and the Editorial Policy Checklist.

Statistics

For all statistical analyses, confirm that the following items are present in the figure legend, table legend, main text, or Methods section.

n/a	Confirmed	
☐	☒	The exact sample size (n) for each experimental group/condition, given as a discrete number and unit of measurement
☐	☒	A statement on whether measurements were taken from distinct samples or whether the same sample was measured repeatedly
☐	☒	The statistical test(s) used AND whether they are one- or two-sided<br>*Only common tests should be described solely by name; describe more complex techniques in the Methods section.*
☐	☒	A description of all covariates tested
☐	☒	A description of any assumptions or corrections, such as tests of normality and adjustment for multiple comparisons
☐	☒	A full description of the statistical parameters including central tendency (e.g. means) or other basic estimates (e.g. regression coefficient) AND variation (e.g. standard deviation) or associated estimates of uncertainty (e.g. confidence intervals)
☐	☒	For null hypothesis testing, the test statistic (e.g. F, t, r) with confidence intervals, effect sizes, degrees of freedom and P value noted<br>*Give P values as exact values whenever suitable.*
☒	☐	For Bayesian analysis, information on the choice of priors and Markov chain Monte Carlo settings
☒	☐	For hierarchical and complex designs, identification of the appropriate level for tests and full reporting of outcomes
☒	☐	Estimates of effect sizes (e.g. Cohen's d, Pearson's r), indicating how they were calculated

Our web collection on statistics for biologists contains articles on many of the points above.

Software and code

Policy information about availability of computer code

Data collection	Data was extracted directly from included studies. If necessary, data were estimated from graphics and standard deviations were determined or recalculated from standard errors.
Data analysis	All analyses were conducted with R 4.2.3. For the meta-analysis itself and meta-regression analysis we used the metafor 4.2-0 package.

For manuscripts utilizing custom algorithms or software that are central to the research but not yet described in published literature, software must be made available to editors and reviewers. We strongly encourage code deposition in a community repository (e.g. GitHub). See the Nature Portfolio guidelines for submitting code & software for further information.

Data

Policy information about availability of data

All manuscripts must include a data availability statement. This statement should provide the following information, where applicable:

- Accession codes, unique identifiers, or web links for publicly available datasets
- A description of any restrictions on data availability
- For clinical datasets or third party data, please ensure that the statement adheres to our policy

All data used in this systematic review and meta-analysis is available from publicly available and herein referenced sources. All data generated or analyzed during this study are included in this published article and its supplementary information files.

Human research participants

Policy information about studies involving human research participants and Sex and Gender in Research.

Reporting on sex and gender	n/a
Population characteristics	n/a
Recruitment	n/a
Ethics oversight	n/a

Note that full information on the approval of the study protocol must also be provided in the manuscript.

Field-specific reporting

Please select the one below that is the best fit for your research. If you are not sure, read the appropriate sections before making your selection.

☒ Life sciences ☐ Behavioural & social sciences ☐ Ecological, evolutionary & environmental sciences

For a reference copy of the document with all sections, see nature.com/documents/nr-reporting-summary-flat.pdf

Life sciences study design

All studies must disclose on these points even when the disclosure is negative.

Sample size	After the systematic review, and application of the inclusion and exclusion criteria, a total of 88 studies were included in the systematic review and meta-analysis.
Data exclusions	We searched the PubMed and Scopus databases to idenify all original articles related to ECM related strategies in animals for cardiac regeneration following myocardial infarction. We used keywords for heart injury, repair, ECM and animal models, without time or language restrictions. References were allocated to two independent reviewers, who screened references for inclusion based on title and abstract according to the exclusion criteria (not in vivo animal studies, not myocardial infarction related studies, not ECM intervention related studies, use of additional regenerative material, and wrong publication type). Full-text copies of all publications eligible for inclusion were subsequently assessed by two independent reviewers, according to exclusion criteria (wrong publication type, not suitable controls, use of additional regenerative material, not ECM intervention related studies, not myocardial injury related studies, non-ischemic model of MI of heart failure, animals used younger than 7 days old, not in vivo animal studies, full text manuscript not retrievable).
Replication	Attempts at replication were not performed. Heterogeneity was estimated by using the I2 and Chi2 statistic.
Randomization	This is a systematic review and meta-analysis. Animals were not allocated into experimental groups
Blinding	This is s systematic review and meta-analysis. Animals or investigators were not blinded.

Reporting for specific materials, systems and methods

We require information from authors about some types of materials, experimental systems and methods used in many studies. Here, indicate whether each material, system or method listed is relevant to your study. If you are not sure if a list item applies to your research, read the appropriate section before selecting a response.

Materials & experimental systems

n/a	Involved in the study	
☒	☐	Antibodies
☒	☐	Eukaryotic cell lines
☒	☐	Palaeontology and archaeology
☐	☒	Animals and other organisms
☒	☐	Clinical data
☒	☐	Dual use research of concern

Methods

n/a	Involved in the study	
☒	☐	ChIP-seq
☒	☐	Flow cytometry
☒	☐	MRI-based neuroimaging

Animals and other research organisms

Policy information about studies involving animals; ARRIVE guidelines recommended for reporting animal research, and Sex and Gender in Research

Laboratory animals	Various laboratory animals were included within this systematic review (mice, rats, pigs), the precise species, strain, sex, and number of laboratory animals used within the various studies included in this systematic review and meta-analysis are described in Supplementary Table 1
Wild animals	n/a
Reporting on sex	Various laboratory animals were included within this systematic review (mice, rats, pigs), the precise species, strain, sex, and number of laboratory animals used within the various studies included in this systematic review and meta-analysis are described in Supplementary Table 1
Field-collected samples	n/a
Ethics oversight	This is a systematic review and meta-analysis. As laboratory animals were not directly used within this study, no ethical approval was required.

Note that full information on the approval of the study protocol must also be provided in the manuscript.
